# ﻿Revision of Neotropical Scythrididae moths and descriptions of 22 new species from Argentina, Chile, and Peru (Lepidoptera, Gelechioidea)

**DOI:** 10.3897/zookeys.1087.64382

**Published:** 2022-02-22

**Authors:** Kari Nupponen1, Pasi Sihvonen

**Affiliations:** 1 Merenneidontie 19 D, FI-02320 Espoo, Finland Unaffiliated Espoo Finland; 2 Finnish Museum of Natural History, P.O. Box 17, Pohjoinen Rautatiekatu 13, 00014 University of Helsinki, Finland University of Helsinki Helsinki Finland

**Keywords:** Biodiversity, COI phylogeny, DNA barcode, morphology, new species

## Abstract

The taxonomy of South American Scythrididae (Lepidoptera: Gelechioidea) is revised, based on external morphology, genitalia, male abdominal segment VIII, and DNA barcodes using genetic distances, BINs, and a tentative molecular phylogeny. Data include both historical and fresh specimens from Argentina, Brazil, Colombia, Chile, Ecuador, Paraguay, and Peru. Thirty-four species are recognised as valid, and the fauna classified in three genera. Type specimens and morphology of all species are described and figured in detail. DNA barcode sequences of the COI gene were successful for 22 species, the average genetic divergence between species being 5.1%. A key to Neotropical Scythrididae species is provided, based on the male genitalia and abdominal segment VIII, which show most and easily accessible interspecific differences.

Our study revealed that the Scythridae fauna of South America is more or less completely unknown. As a result, 22 new species are described, increasing the number of South American Scythrididae species from 13 to 34. All new species are authored by Kari Nupponen (incertae sedis means the genus combination is uncertain and needs further research, country of the type locality is given in parentheses): *Rhamphurasubdimota***sp. nov.** (Argentina), *R.pozohondaensis***sp. nov.** (Argentina), *R.spiniuncus***sp. nov.** (Argentina), *R.angulisociella***sp. nov.** incertae sedis (Argentina), *R.curvisociella***sp. nov.** incertae sedis (Argentina), *R.tetrafasciella***sp. nov.** incertae sedis (Argentina), *Landryiaankylosauroides***sp. nov.** incertae sedis (Argentina), *L.chilensis***sp. nov.** incertae sedis (Chile), *Scythrisdirectiphallella***sp. nov.** (Argentina), *S.furciphallella***sp. nov.** (Argentina), *S.manchaoensis***sp. nov.** (Argentina), *S.salinasgrandensis***sp. nov.** (Argentina), *S.angustivalvella***sp. nov.** (Argentina), *S.caimancitoensis***sp. nov.** (Argentina), *S.lequetepequensis***sp. nov.** (Peru), *S.sanfriscoensis***sp. nov.** (Argentina), *S.tigrensis***sp. nov.** (Argentina), *S.bicoloristrigella***sp. nov.** incertae sedis (Argentina), *S.saldaitisi***sp. nov.** incertae sedis (Argentina), *S.wikstromi***sp. nov.** incertae sedis (Argentina), *S.andensis***sp. nov.** incertae sedis (Argentina), *S.mendozaensis***sp. nov.** incertae sedis (Argentina).

The following new combinations are proposed: *Scythrisdepressa* Meyrick, 1931 and *Scythrisdimota* Meyrick, 1931 are transferred from *Scythris* Hübner, 1825 to *Rhamphura* Landry, 1991 **comb. nov.** Three species classified in *Scythris* earlier are now classified as *Scythris* (incertae sedis): *Scythrisdividua* Meyrick, 1916, *S.medullata* Meyrick, 1916 and *S.notorrhoa* Meyrick, 1921. The taxon *Syntetrernisneocompsa* Meyrick, 1933, recently classified in Scythrididae: *Scythris*, is excluded from Scythrididae and it is now classified in Cosmopterigidae incertae sedis.

## ﻿Introduction

The family Scythrididae has a world-wide distribution, excluding the Antarctic. Scythridids occur also on several isolated islands, such as Hawaii (Walsingham 1907), the Galapagos Islands (Bucheli 2005), and the Maldives (Nupponen and Saldaitis 2013). More than 850 species of Scythrididae are described, but the true diversity of the family is much higher: in various museum collections there are known to be several hundred taxa awaiting description (Landry 1991). Many large areas still remain more or less unexplored, e.g., China, Mongolia, South and South-East Asia, Australia, central parts of Africa, and the majority of South and Central America. The scythridid fauna of the Neotropical realm is poorly known. To date, only thirteen species of *Scythris* Hübner, 1825 have been described from continental South America, all by Edward Meyrick in his monumental works on exotic Microlepidoptera (Meyrick 1912–1916, 1916–1923, 1923–1930, 1930–1936, 1936–1937; Meyrick 1931), from Argentina (1 sp.), Brazil (2 spp.), Colombia (2 spp.), Ecuador (1 sp.), Paraguay (3 spp.), and Peru (6 spp.).

There are few characters discovered in Scythrididae that would unambiguously define the family (Landry 1991; Bengtsson 2014; Heikkilä et al. 2014). In several cases the external appearance of the moth gives the impression of a scythridid: they are more pronouncedly teardrop-shaped, with more pointed wing apices, have an abdomen that extends at least 2/3 of the forewing length, and have narrow head scales compared to Blastobasidae, Cosmopterigidae, and Momphidae (Landry 1991). Scythrididae and Stathmopodidae are considered sister taxa, which is supported by molecular and morphological data, particularly the similarly expanded ductus seminalis (Heikkilä et al. 2014: fig. 6). In the present work, we have followed Landry (1991) and included taxa in Scythrididae if the following diagnostic features were present: base of haustellum scaled; head scales appressed and very narrow; labial palps with article 3 shorter than article 2; R4 and R5 of forewing stalked, R4 extended to costa, R5 to termen; tarsomeres 1–4 with two subapical spurs; phallus ankylosed by juxta or manica; signum absent. All these characters are homoplastic within Gelechioidea if treated alone, but the combination seems unique for Scythrididae. Further, in the male, abdominal segment VIII tergum and sternum are typically modified. A narrow or very narrow ductus bursae in the female genitalia was considered a further diagnostic character of Scythrididae (Landry 1991), but later it was shown that this character is not ubiquitously present in Scythrididae (Kaila 2004). Heikkilä et al. (2014) found a unique synapomorphy in the larva: spiracle on A7 is smaller than other spiracles, and the shape of stipular setae of the larval spinneret being long and thin seems uniform in Scythrididae, and only occasionally observed in single species of other families. We did not include immature stages in our study due to lack of material.

The male genitalia of Scythrididae are notorious for their extraordinary morphological diversification, making interpretations of homology difficult (Landry 1991). Asymmetry is widespread, and among the Lepidoptera, the only known case of antisymmetry has been reported from a Spanish *Scythris* species (Nupponen 2009).

The genus-level classification of Scythrididae is in its infancy. It is estimated that undescribed taxa outnumber described ones by a factor of ten (Landry 1991). This, combined with the lack of a global view and extreme structural heterogeneity, has largely resulted in unsatisfactory dumping of more and more species into an undefined concept of *Scythris*. The generic name has been used in very broad sense and instead of describing new genera, the species group concept has been widely applied (e.g., Jäckh 1977; Bengtsson 2014). Landry (1991) provided a phylogenetic framework for the Nearctic Scythrididae, including descriptions of three new genera. He used informal supraspecific lineages and concluded that his (1991: 206) “initial proposal represents a working hypothesis to be tested by studying more taxa and characters”.

The present paper is based on examination of all of Meyrick’s described *Scythris* material from continental South America, housed in the Natural History Museum London, examination of Nearctic Scythrididae as presented in Landry (1991), and new materials of Scythrididae collected during 2017 and 2019 in the course of three Finnish–Estonian expeditions to Argentina, Chile, and Peru. The aim of the trips was to document the richness of the scythridid fauna at the foothills of the Andes before habitat loss causes fragmented distributional areas of many species, or possibly even extinctions.

## ﻿Materials and methods

### ﻿Material

The Finnish–Estonian expeditions to Argentina and Chile took place from 25 January–7 February 2017, and to Peru from 26 January–5 February 2019, and the Finnish expedition to Argentina during 13–25 September 2019. 30 collecting sites were sampled in areas of central Chile, NW Argentina, and the Andean and coastal regions of central Peru. A total of 145 scythridid specimens were collected during these expeditions.

The material was collected by light trapping at night. Four to five light traps were used every night, with various UV-tubes and LED-lamps, as well as 160 W incandescent lamps. Considerably effort was done to collect material during the day by sweeping vegetation by net.

All Meyrick’s *Scythris* type specimens from South America in The Natural History Museum London (**NHMUK**) were examined and photographed, including the adults and the genitalia mounted on permanent slides. Data of type specimens are detailed under each species below. Because Meyrick’s *Scythris* type specimens have been dissected by J. F. G. Clarke (details are available in Clarke (1965)), we did not do any further dissecting on this material. Adult photographs were arranged by NHMUK staff under the Digital Collections Programme. Landry’s revision (1991) on Nearctic Scythrididae formed the basis of our study with regard to species-level and genus-level taxonomy.

### ﻿Species delimitation and genus combinations

When making taxonomic decisions, we used all available information, including external features such as wing pattern, structural morphology, and new and existing knowledge on genetic variation in DNA barcodes of Scythrididae and the BIN system as implemented on BOLD (Ratnasingham and Hebert 2007, 2013). Sexes were associated based on wing patterns and DNA barcodes. To understand how the names were applied to the taxa described earlier, all *Scythris* species described by Meyrick during 1916–1933 that are stored in the NHMUK were examined.

Assigning species to Scythrididae genera was done as follows. The majority of the new taxa described in this article were DNA barcoded, and those barcodes were analysed in phylogenetic context using the maximum likelihood approach (see ‘DNA barcoding, genetic analyses, and phylogeny’). Our new DNA barcodes were analysed together with all public Scythrididae DNA barcodes available on the Barcode of Life Data System (BOLD v4 http://boldsystems.org/) from North and South America (data extracted in September 2021, *n* = 725, barcodes > 500 bp were included, search term “Scythrididae”). This tentative phylogeny gave a rough estimate on the systematic position of each species (see Suppl. material 2). We then compared our material against the morphological diagnoses and descriptions of relevant genera as in Landry (1991), and other literature as detailed under each species, to combine taxa in genera. We did not describe new genera, because the phylogenetic framework for Scythrididae is in its infancy (Landry 1991). If the genus combination was doubtful, we either classified those in incertae sedis, or in *Scythris*, following Meyrick (1916, 1921, 1928, 1931, 1933), who classified all South American species in *Scythris*. We highlighted the cases where further research is needed.

### ﻿Dissection and photography

The genitalia preparations were made following standard techniques (Robinson 1976). Genitalia were separated from the abdomen, and mostly mounted in ventral aspect, some also in lateral aspect to show structural details not clearly visible in ventral aspect. The abdomen was cut laterally and spread out.

Photographs of adult specimens were taken with a Canon EOS 7D Mark II, MP-E 65 mm EF 100 mm macro lens. Focus stacking was done with Cognisys StackShot and Zerene Stacker, and final image editing with Adobe Photoshop 2021. Images of Meyrick’s adult type specimens in the NHMUK were provided under the museum’s Digital Collection Programme. The genitalia in the research collection of Kari and Timo Nupponen (coll. **NUPP**) were photographed with a Leica DM1000 microscope and integrated Leica DF295 digital camera. The genitalia in coll. NHMUK were photographed in Sackler Imaging Suite using a Zeiss Axioskop. Most genitalia dissections in both coll. NHMUK and coll. NUPP were photographed in 2–6 images in different focal planes and combined into single images using image-stacking software as implemented in Photoshop 2021. Images were edited in Photoshop 2021 and plates were compiled in CorelDraw 2018. Genitalia figures are not in scale.

### ﻿DNA barcoding, genetic analyses, and phylogeny

Tissue samples (dried legs) of 87 specimens were sent to the Canadian Centre for DNA barcoding (**CCDB**, Biodiversity Institute of Ontario, University of Guelph). DNA extraction, PCR amplification and sequencing of the barcode region of the mitochondrial cytochrome oxidase I (COI) gene (658 base pair region near the 5’ terminus of the COI gene) were carried out following standard high-throughput protocols (deWaard et al. 2008). The taxonomic and collection data, voucher image, COI sequences, and other metadata including sex are provided on the BOLD database https://v4.boldsystems.org through the public dataset DS-SCYNEO “Scythrididae of South America”, https://dx.doi.org/10.5883/DS-SCYNEO. These data were compared with public DNA barcodes of all other Scythrididae material available on BOLD in September 2021. Suppl. material 1 contains GenBank accession numbers (MW564588–MW564622). Analytical tools on BOLD under taxon ID tree (Kimura 2-parameter model), barcode gap analysis and BIN were utilised for genetic analyses (Ratnasingham and Hebert 2007, 2013). Genetic distances between species are reported as minimum pairwise distances, while intraspecific variation is reported as maximum pairwise distances. Genetic distances of the barcodes developed for this article were visualised using the taxon ID tree tool on BOLD and finalised in CorelDraw 2021 (Fig. 81).

For phylogenetic analysis, COI sequences were aligned with MUSCLE implemented in MEGA6 (Tamura et al. 2013). Maximum likelihood (ML) analysis was carried out in the IQ-TREE web server (http://iqtree.cibiv.univie.ac.at; Trifinopoulos et al. 2016). The best substitution model was selected automatically by ModelFinder (Kalyaanamoorthy et al. 2017) as implemented in IQ-TREE. The best-fit model was identified as ‘GTR+F+I+G4’ for COI. To construct the phylogenetic tree, ML analysis with ultrafast bootstrap approximation model UFBoot (1,000 replicates) was applied (Minh et al. 2013). The tree was generated using FigTree v.1.4.2 (Rambaut 2015) and modified using Corel Draw 2021.

### ﻿Designation of types and terminology

When possible, holotypes of new species were chosen among dissected specimens with full-length barcodes. The material is deposited in the research collection of Kari and Timo Nupponen (coll. NUPP, Espoo, Finland), to be deposited in MZH. The coordinates are presented in degrees and decimal minutes.

The terminology used here mainly follows Klots (1970), Landry (1991), Kristensen (2003), and is applied as in Bengtsson (2014) and Nupponen (2018). When homologies were difficult to interpret, we used descriptive terms instead. Under descriptions the prefix “sub” means that the structure in question resembles, or is close to, the mentioned shape. For instance, subtriangular means that shape is close to triangular. The term ‘dirty white’ is used to describe the colour on the ventral side of the abdomen in many species. This colour is white mixed with various tones of grey, and is reminiscent of snow blanket at late spring at forests in southern Finland. Meyrick calls that colour as ‘cloudy white’, but the variation of cloud colour is wider than forest snow.

### ﻿Abbreviations

**JFGC** John Frederick Gates Clarke.

**MZH**Finnish Museum of Natural History, University of Helsinki, Finland.

**NHMUK**The Natural History Museum, London, UK.

**NUPP** research collection of Kari and Timo Nupponen, Espoo, Finland.

**ZMUC**Zoological Museum, Natural History Museum of Denmark, Copen­hagen, Denmark.

## ﻿Results

Altogether 145 specimens representing 25 species were recorded during the expeditions; 22 species/130 specimens in Argentina, 1 species/1 specimen in Chile, and 3 species/14 specimens in Peru. DNA barcodes were obtained for 35 specimens representing 22 species (Fig. 81, Suppl. material 2). Examination of our material against the earlier described fauna revealed that 22 of our species are undescribed. As a result, the described Neotropical Scythrididae fauna increases from 13 (Meyrick 1916, 1921, 1928, 1931, 1933; Landry 1991) to 34 species in continental South America, which is an increase of 162%. The expeditions rediscovered three species described by Meyrick, confirmed by morphology: *Scythrisdepressa* (classified here as *Rhamphuradepressa*, Meyrick recorded it from Paraguay, we report it from Argentina), *S.medullata* (classified here as *Scythris* (incertae sedis) *medulla*, Meyrick reported it from Peru, Colombia and Ecuador, we report it from Peru and Argentina) and *S.tibicina* (Meyrick reported it from Peru, we report it from Peru).

The examined Neotropical specimens are externally similar to their congeners elsewhere in the world. Forewings of many species have different shades of brown, beige and sand, with rather diffuse pale blotches or an elongate longitudinal streak along the fold. Hindwings are lanceolate with a sharp apex, and with long fringes. The male genitalia and abdominal segment VIII are extremely diverse, often asymmetrical, and homologies are often difficult to establish. In many species the phallus is reduced in size, often to the degree that it is difficult to identify.

In the explored South American areas, all observed Scythrididae species are nocturnal. Considerable effort was made to find moths during the day, but none were encountered, even when vegetation was swept with a net. Out of three different light models used, the UV light tubes proved to attract Scythrididae most effectively. Based on our experience, the night-active species on the lower slopes of the Andes are virtually impossible to detect without light traps because shrubs and many herbaceous plants are thorny and prickly.

Our COI maximum likelihood phylogeny is limited in terms of molecular data, but the tree is well-resolved and the support for the nodes is reasonable, judged by the UFBoot support values shown in Suppl. material 2. In our analysis, taxa named as *Arotrura* on BOLD forms the most basal Scythrididae lineage, agreeing with the cladistic hypothesis of Landry (1991: fig. 450). This is sister to *Rhamphura* and all other Scythrididae lineages, and *Landryia* is among the most apical lineages in both our COI maximum likelihood tree and in the cladistic analysis of Landry (1991). *Scythris* was recovered as a large monophyletic genus, but with several genetically distant lineages. Our Neotropical taxa are scattered throughout the tree with other American Scythrididae, but often the South American taxa cluster together within bigger clades. For instance, this is the case in *Rhamphura*.

Those South American taxa, which did not fit any of the genera diagnosed by Landry (1991), are now highlighted with their tentative phylogenetic position based on their barcodes (Suppl. material 2), waiting for further research. The average genetic distance between DNA barcoded species was 5.1% (min. 2.5%, max. 7.4%) according to the barcode gap analysis as implemented on BOLD.

### ﻿Key to Neotropical Scythrididae based on characters of the male genitalia and abdominal segment VIII

**Table:** 

1	Valvae asymmetrical	**2**
–	Valvae symmetrical	**12**
2	Valvae narrow, long (Figs 56–59)	**3**
–	Valvae wide, short or medium length (e.g., Figs 43, 51, 55, 60)	**6**
3	Gnathos sclerotised, straight, ventral margin tooth-like extensions (Fig. 59)	***Scythrisandensis***
–	Gnathos sclerotised, upcurved, ventral margin smooth (Figs 56–58)	**4**
4	Sternum VIII posterior extensions wide, bare (Fig. 58)	***Scythriswikstromi***
–	Sternum VIII posterior extensions narrow, setose (Figs 56, 57)	**5**
5	Sternum VIII posterior extensions without extended base (Fig. 56)	***Scythrisbicoloristrigella***
–	Sternum VIII posterior extensions with extended base (Fig. 57)	***Scythrissaldaitisi***
6	Sternum VIII with anterior apodemes, apex widened (Figs 42, 43)	**7**
–	Sternum VIII without anterior apodemes (e.g., Figs 57, 60)	**8**
7	Posterior margin of sternum VIII V-shaped, left arm setose (Fig. 42)	***Landryiaankylosauroides***
–	Posterior margin of sternum VIII U-shaped, both arms bare (Fig. 43)	***Landryiachilensis***
8	Valvae entirely setose, weakly sclerotised (Fig. 55)	***Scythristigrensis***
–	Valvae partly setose, strongly sclerotised (Figs 51, 60–62)	**9**
9	Valvae apex pointed, bare (Fig. 5151)	***Scythrisinanima***
–	Valvae apex rounded, with long setae (Figs 60–62)	**10**
10	Basal portion of sternum VIII bare (Fig. 62)	***Scythrisnotorrhoa***
–	Basal portion of sternum VIII with sclerotisations, either arched (Fig. 60) or V-shaped (Fig. 61)	**11**
11	Sternum VIII posteriorly with two bifurcate process (Fig. 60)	***Scythrisdividua***
–	Sternum VIII posteriorly with one bifurcate process (Fig. 61)	***Scythrismedullata***
12	Valvae pointing upwards, sternum VIII with triangular process at middle (Fig. 54)…………	***Scythrissanfranciscoensis***
–	Valvae pointing laterally or downwards, sternum VIII without triangular process (Figs 35–41, 44–50, 52, 53)	**13**
13	Valvae with sub-oval bristled extension (Fig. 53)	***Scythristibicina***
–	Valvae without sub-oval bristled extension (Figs 35–41, 44–50, 52)	**14**
14	Posterior margin of sternum VIII pointed (Fig. 50)	***Scythrisfluvialis***
–	Posterior margin of sternum VIII not pointed (Figs 35–41, 44–49, 52)	**15**
15	Posterior margin of tergum VIII folded, covered by minute spines (Fig. 52)	***Scythrislequetepequensis***
–	Posterior margin of tergum VIII not folded, not covered by minute spines (Figs 35–41, 44–49)	**16**
16	Sternum and tergum VIII simple (Fig. 40)	***Rhamphuraangulisociella***
–	Sternum and tergum modified (Figs 35–39, 41, 44–49)	**17**
17	Sternum VIII with anteriorly directed apodemes (Figs 35–39)	**18**
–	Sternum VIII without anteriorly directed apodemes (Figs 41, 44–49)	**22**
18	Tergum VIII 3-pronged (Fig. 37)	***Rhamphurasubdimota***
–	Sternum VIII not 3-pronged (Figs 35, 36, 38, 39)	**19**
19	Valvea with dorsal setose lobes (Fig. 38)	***Rhamphuraimmunis***
–	Valvae without dorsal lobes (Figs 35, 35, 39)	**20**
20	Uncus triangular (Fig. 36)	***Rhamphuradimota***
–	Uncus bifurcate (Figs 35, 39)	**21**
21	Valvae long, constant width, apex round (Fig. 35)	***Rhamphuradepressa***
–	Valvae long, tapering, apex pointed (Fig. 39)	***Rhamphuraspiniuncus***
22	Socii long, curved (Fig. 41)	***Rhamphuracurvisociella***
–	Socii absent (Figs 44–49)	**23**
23	Valvae very long, blade-like, sternum VIII anteriorly deeply indented (Fig. 49)	***Scythriscaimancitoensis***
–	Valvae not very long, not blade-like, sternum VIII anteriroly weakly concave (Figs 44–48).	**24**
24	Valvae narrow, inner margin evenly curved, apex thorn-like (Fig. 44)	***Scythrisdirectiphallella***
–	Valvae wide or narrow, inner margin with extension, apex round (Figs 45–48)	**25**
25	Uncus large, bilobed, valvae subapically with small triangular tooth (Fig. 48)	***Scythriszeugmatica***
–	Uncus small, bilobed, valvae subapically with large horn or lobe (Figs 45–47)	**26**
26	Valvae subapically with large dorsally directed lobe (Fig. 45)	***Scythrisfurciphallella***
–	Valvae subapically with ventrally directed horn (Figs 46, 47)	**27**
27	Valvae apex with distinctly enlarged lobe, posterior appendices of sternum VIII converging (Fig. 46)	***Scythrismanchaoensis***
–	Valvae apex with weakly enlarged lobe, posterior appendices of sternum VIII diverging (Fig. 47)	***Scythrisangustivalvella***

Males of the following species are unknown: *Scythrisejiciens* Meyrick, *Scythrismendozaensis* sp. nov., *Scythrisplocogastra* Meyrick, *Rhamphurapozohondaensis* sp. nov., *Scythrissalinasgrandensis* sp. nov., *Rhamphuratetrafasciella* sp. nov.

A key to the female genitalia is not given, as the female of only 11 of 35 recognised species is known.

### ﻿Taxonomy

The phylogenetic relationships of South American Scythrididae are currently inadequately resolved, making the genus classification difficult. Our approach to combine the DNA barcode phylogeny with morphology, mostly utilising the genitalia, abdominal segments VII and VIII and wing patterns and compared against the diagnoses in Landry (1991), gives an indicative first step in an iterative approach to solve the relationships of studied taxa. More genetic data are needed, and global taxon sampling on Scythrididae, to build a robust support for the evolutionary relationships.

We present the genera in the order roughly following our COI maximum likelihood phylogeny (Suppl. material 2) and the phylogenetic hypothesis of Landry (1991): *Rhamphura*, *Rhamphura* incertae sedis, *Landryia* incertae sedis, *Scythris*, *Scythris* incertae sedis. Within each genus we first present species groups (if any), arranged alphabetically by species, and then isolated species are presented alphabetically by species. We exclude one species from Scythrididae, and this is treated at the end.

#### Checklist of South American Scythrididae

##### *Rhamphura* Landry, 1991

**The *depressa* species group**

*Rhamphuradepressa* (Meyrick, 1931), comb. nov.

*Rhamphuradimota* (Meyrick, 1931), comb. nov.

*Rhamphurasubdimota* Nupponen, sp. nov.

**Not assigned to a species group**

*Rhamphuraimmunis* (Meyrick, 1916)

*Rhamphurapozohondaensis* Nupponen, sp. nov.

*Rhamphuraspiniuncus* Nupponen, sp. nov.

*Rhamphuraangulisociella* Nupponen, sp. nov., genus combination incertae sedis

*Rhamphuracurvisociella* Nupponen, sp. nov., genus combination incertae sedis

*Rhamphuratetrafasciella* Nupponen, sp. nov., genus combination incertae sedis

##### *Landryia* Kemal & Koçak, 2006

**The *ankylosauroides* species group**

*Landryiaankylosauroides* Nupponen, sp. nov., genus combination incertae sedis

*Landryiachilensis* Nupponen, sp. nov., genus combination incertae sedis

##### *Scythris* Hübner, 1825

**The *directiphallella* species group**

*Scythrisangustivalvella* Nupponen sp. nov.

*Scythrisdirectiphallella* Nupponen, sp. nov.

*Scythrisfurciphallella* Nupponen, sp. nov.

*Scythrismanchaoensis* Nupponen, sp. nov.

*Scythrissalinasgrandensis* Nupponen, sp. nov.

*Scythriszeugmatica* Meyrick, 1931

**Not assigned to a species group**

*Scythriscaimancitoensis* Nupponen, sp. nov.

*Scythrisejiciens* Meyrick, 1928

*Scythrisfluvialis* Meyrick, 1916

*Scythrisinanima* Meyrick, 1916

*Scythrislequetepequensis* Nupponen, sp. nov.

*Scythrisplocogastra* Meyrick, 1931

*Scythristibicina* Meyrick, 1916

*Scythrissanfranciscoensis* Nupponen, sp. nov.

*Scythristigrensis* Nupponen, sp. nov.

**The *bicoloristrigella* species group**

*Scythrisbicoloristrigella* Nupponen, sp. nov., genus combination incertae sedis

*Scythrissaldaitisi* Nupponen, sp. nov., genus combination incertae sedis

*Scythriswikstromi* Nupponen, sp. nov., genus combination incertae sedis

**The *andensis* species group**

*Scythrisandensis* Nupponen, sp. nov., genus combination incertae sedis

*Scythrismendozaensis* Nupponen, sp. nov., genus combination incertae sedis

**The *dividua* species group**

*Scythrisdividua* (Meyrick, 1916), genus combination incertae sedis

*Scythrismedullata* (Meyrick, 1916), genus combination incertae sedis

*Scythrisnotorrhoa* (Meyrick, 1921), genus combination incertae sedis

#### Taxonomic treatments

***Rhamphura* Landry, 1991**

##### The *depressa* species group

Valvae narrow and straight, distal 1/3 somewhat broadened dorsally. Male sternum VIII rectangular basally, lateral reinforcement extended anteriorly forming prongs. Phallus short and thick. Tegumen laterally with parallel and heavily sclerotised processes (absent in *depressa*). Anteriorly to tegumen attached a large formation, consisting of two parallel, basally fused sclerotised pouches (absent in *depressa*). Species included: *depressa*, *dimota*, *subdimota*.

###### ﻿ Rhamphura depressa

Taxon classificationAnimaliaLepidopteraScythrididae

(Meyrick, 1931), comb. nov.

CE18C4ED-A78D-56E0-9CA1-AAFE2CD3030D

Figs 1 , 35

Scythris depressa Meyrick, 1931. Zoological Journal of the Linnean Society 37: 282.

####### Material examined.

***Holotype*.** Paraguay • ♂; Chaco region, Makthlawaiya; GSC [G. S. Carter]; 11.26.; [genitalia slide] JFGC No. 8061; NHMUK ID 010922355; NHMUK slide ID 010316669; coll. NHMUK.

####### Other material.

Argentina • 2 ♂; prov. Santiago del Estero, Pozo Honda village S, by salt lake; 27°17.2'S, 64°28.0'W, 260 m a.s.l.; 19 Sep. 2017; K. Nupponen & R. Haverinen leg.; [BOLD sample ID] KN01044; [genitalia slide] K. Nupponen prep. no. 2/9 Dec. 2019; coll. NUPP (MZH).

####### Diagnosis.

Externally hardly separable from *R.dimota* and *R.subdimota*. Reliable determination can be achieved by genitalia examination (DNA barcode not available for *R.dimota* yet). Gnathos is labiate, short and sclerotised in *R.depressa*; gnathos base is triangular hood, distal arm is short and bent in *R.dimota*; absent in *R.subdimota*. Lateral processes of tegumen absent in *R.depressa*; triangular, granulate and heavily sclerotised in *R.dimota*; sub-oval, granulate, with longitudinal cleavage and heavily sclerotised in *R.subdimota*. Male tergum VIII trapezoid in *R.depressa*; rectangular with long diverging anterior apodemes in *R.dimota* ((note: structures shown are not in comparable position, potentially deformed during dissection); pentagonal and medioposteriorly extended in *R.dimota*).

####### Description.

The original description is quoted: “Wingspan 11 mm ♂. Head and thorax dark purplish-grey, sternum white. Palpi dark grey, basal joint and basal half of second white. Abdomen blackish, anal tuft grey segmental margins on ventral surface pale ochreous-grey. Forewings dark purplish-grey; a few whitish scales on fold towards middle: cilia grey. Hind wings 0.6, 4 and 5 separate; dark grey; cilia grey.”

***Male genitalia*.** Uncus large, bifurcate; united by transverse sclerotisation. Gnathos labiate, short and sclerotised. Anteriorly to tegumen attached a large formation, consisting of two parallel, curved, medially fused pouches. Phallus short and thick, vase-shaped. Valvae symmetrical, long and slender, of constant width, tip rounded and setose. Sternum VIII rectangular basally, posterior reinforcement extended laterally; lateral apodemes sclerotised and extended anteriorly forming prongs with spoon-shaped apices. Tergum VIII trapezoid plate, posterior margin with numerous minute setae.

####### Distribution.

Argentina, Paraguay.

####### Habitat.

In Argentina the species was collected in a dry bushy area near a salt lake shore (Fig. 78).

####### Genetic data.

BIN: BOLD:ADY6755 (*n* = 2 from Argentina). Maximum intraspecific variation 0%. Nearest neighbour: North American *Rhamphura* sp. (Scythrididae, BIN: BOLD:AAA9059, 2.89%).

####### Remarks.

New to Argentina. Female unknown. Based on COI maximum likelihood phylogeny, the South American taxa *subdimota*, *depressa*, *pozohondaensis*, *spiniuncus*, *angulisociella*, *tetrafasciella* and *curvisociella* group together, associating next to the North American taxa classified in *Rhamphura* on BOLD (Suppl. material 2). Structurally these taxa are heterogeneous and the external characters, male and/or female genitalia show varying degrees of similarities to North American *Rhamphura*, as diagnosed and illustrated in Landry (1991). With regard to *depressa*, it has male sternum VIII with long, anteriorly directed, free apodemes, which is diagnostic in *Rhamphura*. For these reasons, we reclassified *Scythrisdepressa* Meyrick, 1931 as *Rhamphuradepressa* (Meyrick, 1931), new combination.

###### ﻿ Rhamphura dimota

Taxon classificationAnimaliaLepidopteraScythrididae

(Meyrick, 1931), comb. nov.

C594AE41-22E2-5EA0-91FA-42BF49E94F69

Figs 2 , 36

Scythris dimota Meyrick, 1931. Zoological Journal of the Linnean Society 37: 282.

####### Material examined.

***Lectotype*.** Paraguay • ♂; Chaco region, Makthlawaiya; •; GSC [G. S. Carter]; 5.27.; [genitalia slide] JFGC No. 8062; NHMUK ID 010922356; NHMUK slide ID 010316670; coll. NHMUK.

***Paralectotype*.** Paraguay • 1 ♂; same data as for lectotype; coll. NHMUK.

####### Diagnosis.

Externally hardly separable from *R.dimota* and *R.subdimota*. Reliable determination can be achieved by genitalia examination (DNA barcode not available for *R.dimota* yet). Gnathos is labiate, short and sclerotised in *R.depressa*; gnathos base is triangular hood, distal arm is short and bent in *R.dimota*; absent in *R.subdimota*. Lateral processes of tegumen absent in *R.depressa*; triangular, granulate and heavily sclerotised in *R.dimota*; sub-oval, granulate, with longitudinal cleavage and heavily sclerotised in *R.subdimota*. Male tergum VIII trapezoid in *R.depressa*; rectangular with long diverging anterior apodemes in *R.dimota* ((note: structures shown are not in comparable position, potentially deformed during dissection); pentagonal and medioposteriorly extended in *R.dimota*).

####### Description.

The original description is quoted: “Wingspan 12 mm ♂, ♀. Head and thorax bronzy-fuscous, some white scales on posterior edge of thorax. Palpi dark fuscous, basal joint and base of second ochreous-white. Abdomen dark fuscous, ♂ beneath ochreous-white except last two segments. Forewings dark purplish-fuscous; a white streak along fold from base to near middle of wing, ♂ thicker and irregular, and its apex connected with dorsum by irregular white suffusion; some cloudy white suffusion about end of fold and tornus: cilia rather dark grey. Hindwings 0.66, 4 and 5 separate; dark fuscous; cilia rather dark grey.”

***Male genitalia*.** Uncus triangular. Gnathos base small triangular hood; distal arm short, bent, tip pointed. Tegumen hood-shaped, laterally broadly thickened, with two parallel triangular and heavily sclerotised processes. Between tegumen and valvae large formation, consisting of two parallel elongated, basally fused sclerotised pouches. Phallus short and thick, weakly sclerotised (illustrated in Clarke (1965: 472, fig. 4a)). Valvae ~ 1.5 × as long as tegumen and uncus together; narrow and straight, distal 1/3 somewhat broadened dorsally, apex slightly elongated and setose. Vinculum arched, short. Sternum VIII rectangular basally, posterior reinforcement extended laterally, lateral apodemes sclerotised and extended anteriorly forming prongs with spoon-shaped apices. Tergum VIII rectangular, ~ 2 × as wide as long, with long, diverging anterior apodemes.

####### Distribution.

Paraguay.

####### Remarks.

Female unknown. The original description states that one male and one female were available, but Clarke (1965) reported that both are males. The asymmetry in the male valvae (Fig. 36) is an artefact of preparation due to a partly folded left valva on the slide mount. *R.dimota* is morphologically similar to *R.depressa*, particularly the bronzy-fuscous wings, long and narrow valvae and free apodemes on sternum VIII. For these reasons, we reclassify *Scythrisdimota* Meyrick, 1931 as *Rhamphuradimota* (Meyrick, 1931) new combination.

###### ﻿ Rhamphura subdimota

Taxon classificationAnimaliaLepidopteraScythrididae

Nupponen sp. nov.

DAC4A2B9-6C22-5A56-94C9-512028B5753A

http://zoobank.org/18C0487C-633F-455C-83B9-CDDBC05A9004

Figs 3 , 37

####### Type material.

***Holotype*.** Argentina • ♂; prov. Santiago del Estero, Pozo Honda village S, by salt lake; 27°17.2'S, 64°28.0'W; 260 m a.s.l.; 19 Sep. 2017; K. Nupponen & R. Haverinen leg.; [BOLD sample ID] KN01046; [genitalia slide] K. Nupponen prep. no. 5/12 Dec. 2019; coll. NUPP (MZH).

####### Diagnosis.

Externally hardly separable from *R.depressa* and *R.dimota*. Reliable determination can be achieved by genitalia examination (DNA barcode not available for *R.dimota* yet). Gnathos is labiate, short and sclerotised in *R.depressa*; gnathos base is triangular hood, distal arm is short and bent in *R.dimota*; absent in *R.subdimota*. Lateral processes of tegumen is absent in *R.depressa*; triangular, granulate and heavily sclerotised in *R.dimota*; sub-oval, granulate, with longitudinal cleavage and heavily sclerotised in *R.subdimota*. Tergum VIII is trapezoid in *R.depressa*; rectangular with long diverging anterior apodemes in *R.dimota* ((note: structures shown are not in comparable position, potentially deformed during dissection); pentagonal and medioposteriorly extended in *R.dimota*).

####### Description.

Wingspan 10 mm. Head dark brown, laterally mixed with white. Neck tuft and haustellum white. Collar and tegula dark brown with scattered cream scales. Thorax dark brown. Scape dorsally dark brown, ventrally dirty white; pecten dirty white and a little longer than diameter of scape. Flagellum dark brown, 0.65 × length of forewing, ciliate, sensillae ~ 1/2 as long as diameter of flagellum. Labial palp white, except lower surface of palpomeres II and III dark brown. Legs: lower surfaces white, otherwise fuscous with scattered dirty white, except upper surface of forelegs dark brown. Abdomen dorsally fuscous, ventrally dirty white. Forewing dark brown; fold indistinctly cream from base to cell end; small blackish spot under fold at 0.25, 0.45, 0.6, and above tornus. Hindwing dark fuscous.

***Male genitalia*.** Uncus triangular, projected. Gnathos absent (not detected). Tegumen hood-shaped, anterior margin medially deeply concave with heavily sclerotised minute spine at left margin of incurvation; laterally two parallel sub-oval and heavily sclerotised processes with longitudinal cleavage, surface spinuliform. Anteriorly to tegumen attached a large formation, consists of two parallel round, basally fused sclerotised pouches; at base two small and heavily sclerotised triangular extensions. Phallus short, apex somewhat extended, tip pointed. Valvae symmetrical; 1.4 × longer than tegumen and uncus together; basal 0.65 of constant width, distal 1/3 dorsally slightly broadened, apex slightly lobate. Saccus arched, short. Sternum VIII rectangular basally, posterior reinforcement extended laterally; anterior apodemes with spoon-shaped apices. Tergum VIII pentagonal basally, anterior margin widely concave; medioposteriorly long and tapered extension.

####### Etymology.

A participle in nominative singular. The species name alludes to a close relationship with *S.dimota*, based on morphology of the male genitalia.

####### Distribution.

NW Argentina.

####### Habitat.

The collecting site is a dry shrubby area near a salt lake shore (Fig. 77).

####### Genetic data.

BIN: BOLD:ADZ0695 (*n* = 1 from Argentina). Nearest neighbour: An unidentified *Rhamphura* sp. (Scythrididae) from North America (BIN: BOLD:AAA9059, 2.57%).

####### Remarks.

Female unknown. Based on COI maximum likelihood phylogeny, the South American taxa *subdimota*, *depressa*, *pozohondaensis*, *spiniuncus*, *angulisociella*, *tetrafasciella*, and *curvisociella* group together, associating next to the North American taxa classified in *Rhamphura* on BOLD (Suppl. material 2). Structurally these taxa are heterogeneous and the external characters, male and/or female genitalia show varying degrhees of similarities to the North American *Rhamphura*, as diagnosed and illustrated in Landry (1991). With regard to *subdimota*, it has male sternum VIII with long, anteriorly directed, free apodemes and tergum VIII Y-shaped, both diagnostic in *Rhamphura*. We therefore classified this taxon as *Rhamphurasubdimota*.

###### ﻿ Rhamphura immunis

Taxon classificationAnimaliaLepidopteraScythrididae

(Meyrick, 1916)

47385145-4F13-5515-A2BB-6C3188208F37

Figs 4 , 38

Scythris immunis Meyrick, 1916. Exotic Microlepidoptera, vol. 2 (part 1): 13.

####### Material examined.

***Lectotype*.** Peru • ♂; Oroya; [11°31'S, 75°53'W]; 12200 feet a.s.l.; 5–14.; Parish leg.; [genitalia slide] JFGC No. 8056; NHMUK ID 010922360; NHMUK slide ID 010316668; coll. NHMUK.

***Paralectotype*.** Peru • 1 ♂; same data as for lectotype; coll. NHMUK.

####### Diagnosis.

A small (wingspan 9 mm), dark species externally similar to several other dark species, e.g., *S.inanima*, *S.depressa*, and less contrasting specimens of *S.medullata*. Genitalia dissection is required for confident determination. *Scythrisimmunis* is readily separated from the other described species by details in the male genitalia: long bifurcate teguminal processes with lateral setose extensions; tegumen with pair of beak-like processes dorsally; row of pegs ventrally; valvae with dorsal, setose lobes; sternum VIII with long, anteriorly directed free apodemes.

####### Description.

The original description is quoted: ”Wingspan 9 mm ♂, ♀. Head, palpi and thorax dark grey sprinkled with whitish. Antennal ciliations of ♂ 0.75. Abdomen stout in both sexes, bronzy-grey, beneath suffused and mixed with whitish. Forewings lanceolate; dark grey; two blackish longitudinal streaks from base, upper median, reaching to about 0.75, lower running to tornus, some slight whitish irroration on or between these; a similar less distinct streak above dorsum from base to middle: cilia grey. Hindwings with 4 and 5 separate; in ♂ pale grey, thinly scaled, in ♀ grey; cilia greyish, towards base ochreous-tinged.”

***Male genitalia*.** Tegumen with beak-like processes on posterior margin, row of pegs on ventral margin, apex bifurcate and setose. Phallus straight, short and thick, basal 1/2 tapered. Valva narrow, long, with dorsal setose lobes, freely articulated to vinculum. Sternum VIII rectangular, with long, anteriorly directed free apodemes. Tergum VIII medioposteriorly concave, with group of stout setae on both lateral sides.

####### Distribution.

Peru.

####### Remarks.

Female unknown. *Scythrisimmunis* was combined to *Rhamphura* by Landry (1991). We agree with the classification, because *immunis* has the diagnostic male tegumen with a pair of large beak-like processes extended from the posterior margin and with ventral rows of clusters or pegs. Further, male sternum VIII is sclerotised, with long, anteriorly directed, free apodemes. Meyrick (1916) described the species based on three specimens, stated to include both males and females. Clarke (1965) indicated that all three syntypes are actually males.

###### ﻿ Rhamphura pozohondaensis

Taxon classificationAnimaliaLepidopteraScythrididae

Nupponen sp. nov.

29C91EFF-B214-50EC-8AC0-1193C346F9F0

http://zoobank.org/F40CAB6D-DA8A-4C3F-A0C6-7ADB6861F6F1

Figs 5 , 63

####### Type material.

***Holotype*.** Argentina • ♀; prov. Santiago del Estero, Pozo Honda village S, by salt lake; 27°17.2'S, 64°28.0'W; 260 m a.s.l.; 19 Sep. 2017; K. Nupponen & R. Haverinen leg.; [BOLD sample ID] KN01047; [genitalia slide] K. Nupponen prep. no. 1/14 Dec. 2019; coll. NUPP (MZH).

####### Diagnosis.

Externally easily separated from other species treated herein by the blackish brown forewings with a distinct whitish dirty pale beige streak in fold, and blackish hindwings. In the female genitalia of *S.pozohondaensis*, sterigma resembles that of *S.ankylosauroides*, but differs by parallel triangular posterior flaps (trapezoid flap in *S.ankylosauroides*) and presence of cleavage at anterior tip.

####### Description.

Wingspan 11 mm. Head and thorax blackish brown, Few dirty white scales exist around eye. Collar, neck tuft and tegula dark fuscous, paler than head. Haustellum white. Scape dorsally dark brown, ventrally pale fuscous; pecten dirty cream and ca. as long as diameter of scape. Flagellum dark brown, 0.55 × length of forewing. Labial palp white, except lower surfaces of palpomere III and distal 1/2 of palpomere II dark brown. Legs dirty white, upper surfaces of foreleg and midleg mixed with fuscous. Abdomen dorsally fuscous, ventrally white. Forewing blackish brown, distinct whitish dirty pale beige streak in fold from base to 0.75; scattered dirty pale beige scales at apical 1/3. Hindwing blackish brown.

*Female genitalia*. Sterigma triangular, posterolateral corners laterally elongate; posteriorly two large parallel flaps; anterior tip with narrow cleavage. Ostium small, situated at anterior tip of sterigma. Sternum VII quadrangular; posterior margin shallowly concave. Apophyses anteriores 0.55 × length of apophyses posteriores.

####### Etymology.

Latinised adjective in the nominative singular. The species is named after the type locality, the village of Pozo Honda.

####### Distribution.

NW Argentina.

####### Habitat.

The habitat at the collecting site is a dry shrubby area near a salt lake shore (Fig. 77).

####### Genetic data.

BIN: BOLD:ADY8268 (*n* = 1 from Argentina). Nearest neighbour: *Rhamphuradepressa* (BIN: BOLD:ADY6755, 3.3%).

####### Remarks.

Male unknown. Based on COI maximum likelihood phylogeny, the South American taxa *subdimota*, *depressa*, *pozohondaensis*, *spiniuncus*, *angulisociella*, *tetrafasciella*, and *curvisociella* group together, associating next to the North American taxa classified in *Rhamphura* on BOLD (Suppl. material 2). Structurally these taxa are heterogeneous and the external characters, male and/or female genitalia show varying degrees of similarities to the North American *Rhamphura*, as diagnosed and illustrated in Landry (1991). With regard to *pozohondaensis*, it has female sterigma as triangular cone, projected anteriorly, which is diagnostic in *Rhamphura*. We therefore classified this taxon as *Rhamphurapozohondaensis*.

###### ﻿ Rhamphura spiniuncus

Taxon classificationAnimaliaLepidopteraScythrididae

Nupponen sp. nov.

0323F9C9-9A7E-5F5D-83FE-C80EEF6404DA

http://zoobank.org/06D285D0-42C0-4124-A983-8F2D7646B877

Figs 6 , 39

####### Type material.

***Holotype*.** Argentina • ♂; prov. San Juan, Andes Mts., salt lake by Cordillera del Tigre; 30°52.8'S, 68°52.4'W, 1620 m a.s.l.; 26 Jan. 2017; K. Nupponen & R. Haverinen leg.; [BOLD sample ID] KN01045; [genitalia slide] K. Nupponen prep. no. 4/12 Dec. 2019; coll. NUPP (MZH).

####### Diagnosis.

Wings mottled beige and brown forewing and an indistinct pale beige streak in fold. Genitalia examination is needed for a reliable identification. In the male genitalia, the bifurcate, robust uncus with heavily sclerotised ventral spines and falcate shape of the valvae are unmistakable.

####### Description.

Wingspan 10 mm. Head brown, few white scales around eye. Haustellum pale fuscous. Neck tuft white. Collar, tegula and thorax pale brown. Scape dorsally brown, ventrally pale fuscous; pecten dirty cream and longer than diameter of scape. Flagellum dark brown, 0.55 × length of forewing, ciliate, sensillae ~ 0.65 × as long as diameter of flagellum. Labial palp white, except palpomeres II and III brown at lower surface and terminally. Legs white, shallowly mixed with fuscous. Abdomen whitish fuscous, ventrally paler. Forewing brown of various tones, fold and terminal 1/3 mixed with dirty pale beige scales; indistinct dark brown dash in fold at 0.5; small blackish spot in cell end. Hindwing fuscous. Fringes darker than wings.

***Male genitalia*.** Uncus robust, 0.6 × as long as valva, bifurcate; at base of furcation ~ 15 heavily sclerotised minute spines. Phallus heavily melanised, short. Valva long and narrow, subbasally a little broadened, then evenly tapered, distal quarter bent downwards, tip pointed. Saccus short, semi-circular. Juxta narrow, 0.8 × length of phallus. Sternum VIII basally a rectangular plate; reinforcement continues posterolaterally at backwards directed extensions; long anterolateral apodemes with spatular tips. Tergum VIII trapezoid, 1.4 × wider than high.

####### Etymology.

A noun in apposition. The species name refers to the spinose uncus of the male genitalia.

####### Distribution.

NW Argentina.

####### Habitat.

The collecting site is a xerothermic habitat with sparse halophytic shrubs near a dry salt lake at medium altitude of the Andes (Fig. 78).

####### Genetic data.

BIN: BOLD:ADY6426 (*n* = 1 from Argentina). Nearest neighbour: An unidentified *Rhamphura* sp. (Scythrididae) from North America (BIN: BOLD:AAA9059, 3.05%).

**Figures 1–6.**
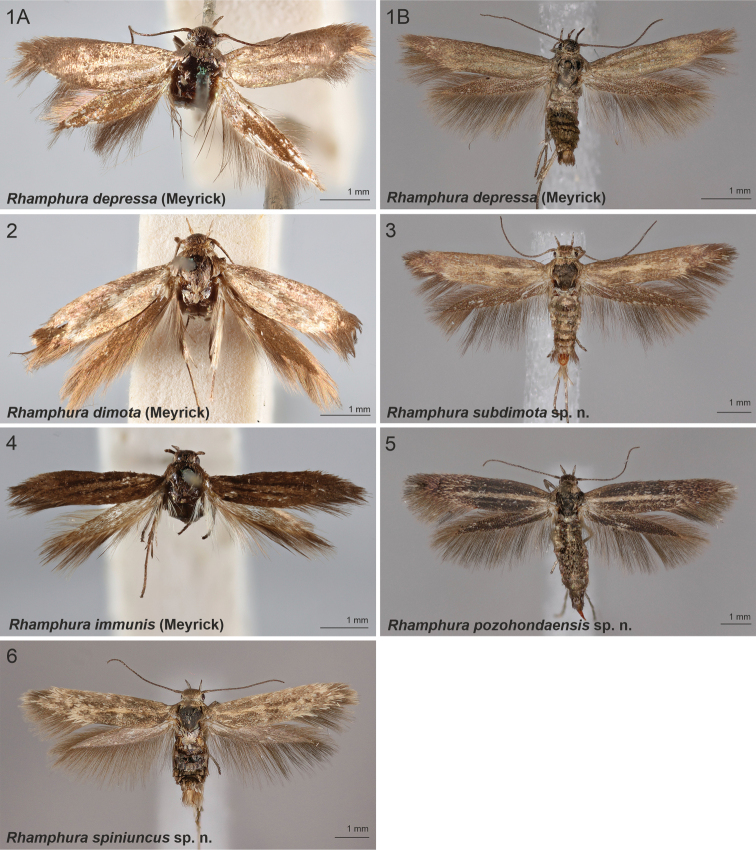
Scythrididae adults, genus *Rhamphura***1A***R.depressa* (Meyrick, 1931), male, holotype **1B***R.depressa* (Meyrick, 1931), male, holotype **2***R.dimota* (Meyrick, 1931), male, lectotype **3***R.subdimota* Nupponen, sp. nov., male, holotype **4***R.immunis* (Meyrick, 1916), male, lectotype **5***R.pozohondaensis* Nupponen, sp. nov., female, holotype **6***R.spiniuncus* Nupponen, sp. nov., male holotype.

####### Remarks.

Female unknown. Based on the COI maximum likelihood phylogeny, the South American taxa *subdimota*, *depressa*, *pozohondaensis*, *spiniuncus*, *angulisociella*, *tetrafasciella*, and *curvisociella* group together, associating next to the North American taxa classified in *Rhamphura* on BOLD (Suppl. material 2). Structurally these taxa are heterogeneous and the external characters, male and/or female genitalia show varying degrees of similarities to the North American *Rhamphura*, as diagnosed and illustrated in Landry (1991). With regard to *spiniuncus*, it has a bifurcate uncus with ventral spines, male sternum VIII with long, anteriorly directed, free apodemes, which are diagnostic for *Rhamphura*. We therefore classified this taxon as *Rhamphuraspiniuncus*.

###### ﻿ Rhamphura angulisociella

Taxon classificationAnimaliaLepidopteraScythrididae

Nupponen, sp. nov., genus combination incertae sedis

CDA43D77-4961-5DF1-B699-7544ADF03B80

http://zoobank.org/10DE3851-DA34-45F8-861B-D7EFF3D4390A

Figs 7 , 40

####### Type material.

***Holotype*.** Argentina • ♂; prov. Jujuy, Rio San Francisco, by Caimancito village; 23°43.8'S, 64°36.3'W; 400 m a.s.l.; 18 Sep. 2017; K. Nupponen & R. Haverinen leg.; [BOLD sample ID] KN01038; [genitalia slide] K. Nupponen prep. no. 1/10 Dec. 2019; coll. NUPP (MZH).

####### Diagnosis.

Externally may be separated from other described taxa by pale brown forewings with characteristic black patches at basal 1/2 of dorsum. In the male genitalia of *R.angulisociella*, anteriorly to tegumen is attached a large formation, which resembles that of *R.depressa*, but *R.angulisociella* has long and angled socii and narrower valvae.

####### Description.

Wingspan 13.5 mm. Head, collar, neck tuft, tegula and thorax pale brown; few white scales around eye and at posterior margin of thorax. Haustellum white with a little pale brown at middle. Scape dorsally dark brown, ventrally dirty cream; pecten dirty cream and longer than diameter of scape. Flagellum dark brown, 0.65 × length of forewing, ciliate, sensillae ~ 1/2 as long as diameter of flagellum. Labial palps white, except lower surface of palpomeres II and III dark brown. Legs with lower surfaces white, otherwise fuscous with scattered dirty white. Abdomen dorsally fuscous, ventrally white, anal tuft pale brown. Forewing pale brown, basal 1/2 between fold and dorsum paler than costal area; irregular black patches at dorsum at 0.2 and 0.5, dorsal and apical areas mixed with sparsely scattered white scales. Hindwing fuscous, darker than forewing.

***Male genitalia*.** Uncus small, semi-circular plate. Socii long and setose shanks, basal 0.75 straight, then bent 80°; anterolaterally bulged with very long setae. Tegumen arched, anterior margin concave, with tuft of long setae posterio-laterally. Note: the following structures are bent 180° (unrolled) ventrally during dissection, which explains why the valvae appear as a dorsal structure in Fig. 40. Anteriorly to tegumen attached a large formation, consists of two parallel sub-ovals, basally fused and posteriorly heavily sclerotised pouches. Phallus short, slightly tapered, tip bent and pointed. Valva longer than uncus and tegumen together, very slender, apical area setose. Sternum VIII rectangular, 1.5 × as wide as high. Tergum VIII trapezoid, anterior margin sclerotised.

####### Etymology.

Diminutive noun in apposition. The species name refers to angular socii in the male genitalia.

####### Distribution.

NW Argentina.

####### Habitat.

The collecting site is a dry river bed surrounded by forests and plantations. Plants of the family Amaranthaceae were frequent at the riverside (Fig. 79).

####### Genetic data.

BIN: BOLD:ADY9489 (*n* = 1 from Argentina). Nearest neighbour: a North American *Rhamphura* sp. (Scythrididae, BIN: BOLD:AAA9059, 4.82%).

####### Remarks.

Female unknown. Based on COI maximum likelihood phylogeny, the South American taxa *subdimota*, *depressa*, *pozohondaensis*, *spiniuncus*, *angulisociella*, *tetrafasciella*, and *curvisociella* group together, associating next to the North American taxa classified in *Rhamphura* on BOLD (Suppl. material 2). Structurally these taxa are heterogeneous and the external characters, male and/or female genitalia show varying degrees of similarities to the North American *Rhamphura*, as diagnosed and illustrated in Landry (1991). With regard to *angulisociella*, the structural differences are notable and we therefore took a conservative view and classified this taxon in *Rhamphura* (incertae sedis), highlighting the need for further research.

###### ﻿ Rhamphura curvisociella

Taxon classificationAnimaliaLepidopteraScythrididae

Nupponen, sp. nov., genus combination incertae sedis

F9B9B0E4-8443-5457-B895-6B39FEBDE1D6

http://zoobank.org/92CA4B67-3C76-4FF7-A771-820303B9CE0B

Figs 8 , 41

####### Type material.

***Holotype*.** Argentina • ♂; prov. Santiago del Estero, Pozo Honda village S, by salt lake; 27°17.2'S, 64°28.0'W; 260 m a.s.l.; 19 Sep. 2017; K. Nupponen & R. Haverinen leg.; [BOLD sample ID] KN01041; [genitalia slide] K. Nupponen prep. no. 1/12 Dec. 2019; coll. NUPP (MZH).

####### Diagnosis.

Beige forewings with dark brown costa do not allow unambiguous identification. In the male genitalia of *R.curvisociella*, a large, ventrally curved and distally split phallus is diagnostic, in addition to long curved socii, and triangular extensions near apex of the valvae. In *R.angulisociella* the socii are angled, and valvae are without triangular extensions near the apex.

####### Description.

Wingspan 12.5 mm. Head beige mixed with pale brown, frons paler. Collar, neck tuft, haustellum, tegula and thorax pale beige, neck tuft slightly paler than head. Scape dorsally dark brown, ventrally beige; pecten beige, as long as diameter of scape. Flagellum mixed with beige and dark brown, 0.7 × length of forewing, ciliate, sensillae ~ 1/2 as long as diameter of flagellum. Labial palp white, except lower surface of palpomere II from 0.5 to 0.8 and middle of palpomere III dark brown. Legs: femur and lower surfaces white, otherwise different shades of beige scattered with pale fuscous. Abdomen dorsally fuscous, ventrally dirty white. Forewing beige; costal belt densely covered by dark brown from base to 0.7, dorsal and apical areas with sparsely scattered dark brown scales; at cell end a small black spot. Hindwing dark fuscous, darker than forewing.

***Male genitalia*.** Uncus heart-shaped setose plate. Gnathos rectangular elongate plate. Socii long recurved processes. Tegumen with deep incision anteromedially. Phallus large, basally heavily sclerotised, slightly bent, apical quarter split and tapered. Valva longer than uncus and tegumen combined, narrow, apical quarter slightly broadened and setose; dorsally with subapical triangular extension. Sternum VIII rectangular, 2 × as wide as high, anterior margin concave, anterolateral margin elongated and somewhat sclerotised. Tergum VIII rectangular, anterior margin concave and reinforced; posterior margin with two parallel setose lobes with wrinkled surface.

####### Etymology.

Diminutive noun in apposition. The species name refers to the curved socii in the male genitalia.

####### Distribution.

NW Argentina.

####### Habitat.

The collecting site is a dry, shrubby area near a salt lake shore (Fig. 77).

####### Genetic data.

BIN: BOLD:ADY6339 (*n* = 1 from Argentina). Nearest neighbour: Unidentified *Scythris* from Argentina (Scythrididae, BIN: BOLD:ACW4357, 4.98%).

####### Remarks.

Female unknown. The ventral and dorsal aspects were difficult to interpret in the male genitalia because only a single male is known, and the structures are distorted under the cover glass. Based on COI maximum likelihood phylogeny, the South American taxa *subdimota*, *depressa*, *pozohondaensis*, *spiniuncus*, *angulisociella*, *tetrafasciella*, and *curvisociella* group together, associating next to the North American taxa classified in *Rhamphura* on BOLD (Suppl. material 2). Structurally these taxa are heterogeneous and the external characters, male and/or female genitalia show varying degrees of similarities to the North American *Rhamphura*, as diagnosed and illustrated in Landry (1991). With regard to *curvisociella*, the structural differences are notable and we therefore took a conservative view and classified this taxon in *Rhamphura* (incertae sedis), highlighting the need for further research.

###### ﻿ Rhamphura tetrafasciella

Taxon classificationAnimaliaLepidopteraScythrididae

Nupponen, sp. nov., genus combination incertae sedis

0F3BD420-F4E9-580D-9DC3-5B7F38C79539

http://zoobank.org/5E53FDE0-EF2C-4DCC-8656-F22115260D1D

Figs 9 , 64

####### Type material.

***Holotype*.** Argentina • ♀; prov. La Rioja, valley east of Sierra de Sanogasta; 29°51.7'S, 67°09.9'W; 670 m a.s.l.; 22 Sep. 2017; K. Nupponen & R. Haverinen leg.; [BOLD sample ID] KN01040; [genitalia slide] K. Nupponen prep. no. 3/13 Dec. 2019; coll. NUPP (MZH).

####### Diagnosis.

Externally distinctive species, readily recognised by four transverse dark brown fasciae on forewing. The female genitalia are characterised by the funnel-shaped sterigma attached anteriorly to an arched plate.

####### Description.

Wingspan 10 mm. Head dark brown, forehead mixed with white. White scales around eye. Neck tuft white. Collar, haustellum, tegula and thorax dark brown with scattered white. Scape dark brown, ventrally mixed with white; pecten as long as diameter of scape. Flagellum dark brown, 0.6 × length of forewing. Labial palp white, palpomere III mixed with dark brown. Legs: femur and tarsi white, each tarsus two dark brown patches at upper surface; tibiae mixed with fuscous. Abdomen anterior 1/2 of each segment dorsally dark brown, otherwise white. Forewing dirty white, cut by four irregular transverse dark brown fasciae belts subbasally, at 0.45, 0.7, and subapically. Hindwing pale fuscous.

***Female genitalia*.** Sterigma funnel-shaped, distally tapered, posterior 1/2 more sclerotised; anteriorly attached to arched sclerotisation. Ostium small, situated at tip of sterigma. Sternum VII trapezoid, 1.3 × wider than high. Apophyses anteriores 0.7 × length of apophyses posteriores.

####### Etymology.

Diminutive noun in apposition. The species name refers to the forewing patterning of the moth.

####### Distribution.

NW Argentina.

####### Habitat.

The collecting site is a xerothermic saline valley at foothills of the Andes, with rather sparse vegetation.

####### Genetic data.

BIN: BOLD:ADZ0119 (*n* = 1 from Argentina). Nearest neighbour: *Scythris* sp. (BIN: BOLD:ADZ0118, 5.65%).

####### Remarks.

Male unknown. Based on COI maximum likelihood phylogeny, the South American taxa *subdimota*, *depressa*, *pozohondaensis*, *spiniuncus*, *angulisociella*, *tetrafasciella*, and *curvisociella* group together, associating next to the North American taxa classified in *Rhamphura* on BOLD (Suppl. material 2). Structurally these taxa are heterogeneous and the external characters, male and/or female genitalia show varying degrees of similarities to the North American *Rhamphura*, as diagnosed and illustrated in Landry (1991). With regard to *tetrafasciella*, the structural differences are notable and we therefore took a conservative view and classified this taxon in *Rhamphura* (incertae sedis), highlighting the need for further research.

###### ﻿ Landryia

Taxon classificationAnimaliaLepidopteraScythrididae

Kemal & Koçak, 2006

33FDB4F4-ED02-54B2-832F-0669FC15FC7D

####### Nomenclatural note.

*Landryia* Kemal & Koçak, 2006 is a replacement name for *Asymmetrura* Landry, 1991 (Kemal and Koçak 2006).

##### The *ankylosauroides* species group

Distal arm of gnathos very long, sigmoid, and at tip round extension covered by minute thorns. Valvae asymmetrical with heavily sclerotised extensions. Male sternum VIII large plate with anterior apodemes. Male tergum VIII posteriorly with long and heavily sclerotised spines. Species included: *ankylosauroides*, *chilensis*.

###### ﻿ Landryia ankylosauroides

Taxon classificationAnimaliaLepidopteraScythrididae

Nupponen, sp. nov., genus combination incertae sedis

271E6A3F-1D8D-5B23-8872-4D971B4C6CE9

http://zoobank.org/5173B006-37BB-467F-A1CF-187F5B523FC2

Figs 10 , 42 , 65

####### Type material.

***Holotype*.** Argentina • ♂; prov. Santiago del Estero, Pozo Honda village S, by salt lake; 27°17.2'S, 64°28.0'W; 260 m a.s.l.; 20 Sep. 2017; K. Nupponen & R. Haverinen leg.; [BOLD sample ID] KN01059; [genitalia slide] K. Nupponen prep. No. 4/13 Jan. 2019; coll. NUPP (MZH).

***Paratypes*.** Argentina • 20 ♂, 9 ♀; same data as for holotype; [BOLD sample ID] KN01060; [genitalia slide] K. Nupponen prep. No. 2/13 Jan. 2019 ♂; coll. NUPP; • 21 ♂, 12 ♀; same data as for holotype except collecting date; 19 Sep. 2017; [BOLD sample IDs] KN01061, KN01062; [genitalia slide] K. Nupponen prep. No. 1/15 Dec. 2019 ♀; coll. NUPP; • 1 ♀; prov. La Rioja, valley east of Sierra de Sanogasta; 29°51.7'S, 67°09.9'W; 670 m a.s.l.; 22 Sep. 2017; K. Nupponen & R. Haverinen leg.; coll. NUPP.

####### Diagnosis.

A pale streak in forewing is diagnostic. In the male genitalia of *L.ankylosauroides*, the S-shaped distal arm of gnathos is distinctive, and similar structure is found only in *L.chilensis*. The two taxa are readily separated by several details in the male genitalia: in *L.ankylosauroides* the left valva is much shorter than the right one (in *L.chilensis* valvae ca. equal length) and the right valva is without large distal lobe (in *L.chilensis* a large distal lobe is present), tergum VIII has narrow lateral arms with melanised spikes (in *L.chilensis* spikes are absent and posterior margin is deeply concave). In the female genitalia of *L.ankylosauroides*, sterigma is an inverted cone, which resembles that of *R.pozohondaensis*, but differs by trapezoid posterior flap (parallel triangular flaps in *R.pozohondaensis*) and absence of cleavage at anterior tip.

####### Description.

Wingspan 10.5–12 mm. Head, collar, tegula and thorax pale fuscous; few white scales around eye, and small blotch of same colour at medioposterior margin of thorax. Neck tuft and haustellum white. Scape dorsally dark brown, ventrally dirty white, pecten longer than diameter of scape. Flagellum dark brown, 0.65 × length of forewing, in male ciliate, sensillae ~ 0.75 × as long as diameter of flagellum. Labial palps: palpomere I white; lower surface of posterior 1/2 of palpomere II and palpomere III dark brown, otherwise white. Legs cream, upper surfaces more or less mixed with different tones of brown. Abdomen dorsally fuscous, ventrally dirty white. Forewing grey, costal area slightly darker than dorsal one; more or less distinct white streak in forewing from base to termen, in dorsal margin edged by interrupted dark brown line; few white scales at apical area. Hindwing pale grey.

***Male genitalia*.** Uncus heavily sclerotised, subtriangular, basal part heart-shaped. Gnathos base small belt; distal arm long, strongly sigmoid (S-shaped), tip club-shaped covered by minute spines. Tegumen rectangular. Phallus 0.7 × length of right valva, straight, shaped as elongated bottle. Valvae asymmetrical, fused at basal 1/2, dorsal margins setose; left valva short, oval; right valva 1.4 × longer than left, of constant width, subapically with small extension, apex bent, heavily sclerotised, tip shallowly indented. Saccus as long as right valva, triangular. Sternum VIII large hexagonal plate, medioposteriorly deeply U-shaped; posterior margin with two asymmetrical and diverging extensions, longer one with numerous long and thin setae; latero-anterior corners with parallel long and narrow extensions, tips spatulate. Tergum VIII trapezoid basally, anterior margin concave; medioposteriorly with digitate extension; mediolaterally at both sides long and upwards directed extensions, distal 1/2 with ~ ten long and heavily sclerotised spiniform setae.

***Female genitalia*.** Sterigma triangular. Ostium small, situated at anterior tip of sterigma. Sternum VII trapezoid; lateroposteriorly small triangular flaps at both sides, anterior corners extended. Sternum VIII with two, suboval, sclerotised plates. Apophyses anteriores 0.35 × length of apophyses posteriores.

####### Etymology.

Latinised adjective in the nominative singular. The species name alludes to the shape of the gnathos arm, reminiscent of the tail of Ankylosauridae (Reptilia: Dinosauria).

####### Distribution.

NW Argentina.

####### Habitat.

The habitat at the type locality of Pozo Honda is a dry shrubby area near a salt lake shore (Fig. 78); the other collecting site is an open valley with halophytic vegetation.

####### Genetic data.

BIN: BOLD:ADZ2684 (*n* = 3 from Argentina). Genetically rather homogenous, maximum variation 0.32%. Nearest neighbour: North American *Landryiamatutella* (Clemens, 1860) (Scythrididae, BIN: BOLD:AAE6120, 1.25%).

####### Remarks.

Based on our COI maximum likelihood phylogeny, the South American taxa *ankylosauroides* and *chilensis* group inside a large clade, whose taxa are classified in *Landryia* on BOLD (Suppl. material 2). However, *ankylosauroides* and *chilensis* do not have the diagnostic morphological characters of *Landryia*, such as a greatly enlarged bulbus ejaculatorius (unless accidentally removed during dissection) in the male genitalia and the pincer-like projections on the caudal margin of female sternum VII (Landry 1991). Also, male sternum VIII of *ankylosauroides* and *chilensis* are distinct with their spiniform setae and long apodemes, but such are not present in North American *Landryia* (Landry 1991). Further, North American *L.matutella*, which is genetically the nearest neighbour to taxon *ankylosauroides*, is morphologically different. We therefore classified these two taxa in *Landryia* (incertae sedis), highlighting the need for further research.

###### ﻿ Landryia chilensis

Taxon classificationAnimaliaLepidopteraScythrididae

Nupponen, sp. nov., genus combination incertae sedis

7675931F-228F-56F0-B281-2EF1645C20B3

http://zoobank.org/F4FCD502-354B-4B1B-812B-DF54C443D4EC

Figs 11 , 43

####### Type material.

***Holotype*.** Chile • ♂; Coquimbo district, near Comparbala village; 30°52.4'S, 71°10.9'W; 660 m a.s.l.; 1 Feb. 2017; K. Nupponen & R. Haverinen leg.; [BOLD sample ID] KN01096; [genitalia slide] K. Nupponen prep. no. 3/18 Dec. 2019; coll. NUPP (MZH).

####### Diagnosis.

Wings without any distinct pattern, and may be confused with several patternless, similarly sized species, e,g., *S.tigrensis*. In the male genitalia of *L.chilensis*, the shape of distal arm of the gnathos is distinctive; a similarly shaped narrow and curved gnathos is found only in *L.ankylosauroides.* The two taxa are readily separated by several details in the male genitalia: in *L.chilensis*, the valvae are subequal in length (in *L.ankylosauroides* the left valva is much shorter), the right valva has large sclerotised lobe (absent in *L.ankylosauroides*) , and shape of both tergum VIII and sternum VIII are unique.

####### Description.

Wingspan 14.5 mm. Head, collar, haustellum, tegula and thorax fuscous mixed with dirty white. Few white scales exist around eye. Neck tuft white. Scape fuscous mixed with dirty white, pecten pale cream and longer than diameter of scape. Flagellum dark brown, 0.65 × length of forewing, ciliate, sensillae 1/2 as long as diameter of flagellum. Labial palps with palpomere I and base of palpomere II white, otherwise fuscous more or less mixed with white. Legs fuscous, lower surface suffused with dirty white. Abdomen dorsally lead grey, each segment posteriorly edged by greyish white; ventrally dirty white. Forewing narrow, grey; scattered with dirty white scales densely in fold and at apical area, and sparsely in costal area. Hindwing fuscous.

**Figures 7–11.**
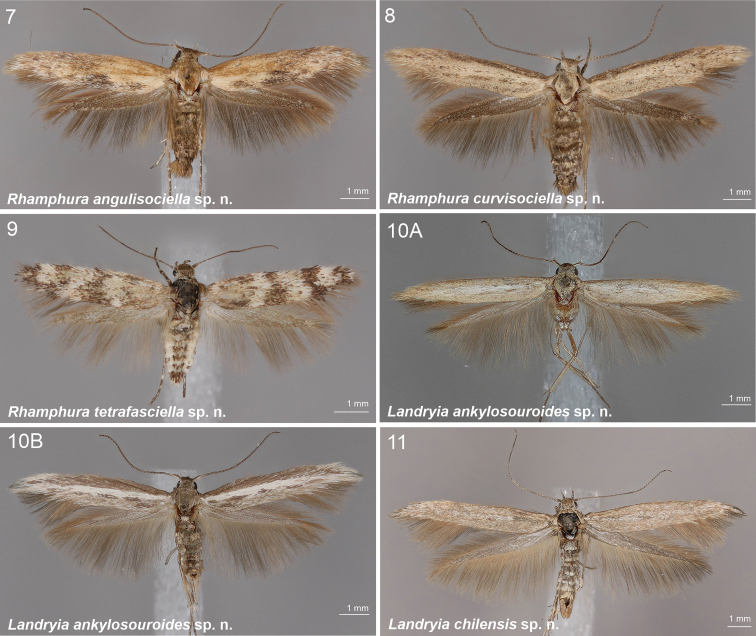
Scythrididae adults, genera *Rhamphura* and *Landryia***7***R.angulisociella* Nupponen, sp. nov., genus combination incertae sedis, male, holotype **8***R.curvisociella* Nupponen, sp. nov., genus combination incertae sedis, male, holotype **9***R.tetrafasciella* Nupponen, sp. nov., genus combination incertae sedis, female, holotype **10A***L.ankylosauroides* Nupponen sp. nov., genus combination incertae sedis, male, holotype **10B***L.ankylosauroides* Nupponen, sp. nov., genus combination incertae sedis, male, paratype **11***L.chilensis* Nupponen, sp. nov., genus combination incertae sedis, male, holotype.

***Male genitalia*.** Uncus small, heavily sclerotised rectangular plate. Gnathos base uneven plate; distal arm 1.65 longer than valva, sigmoid and somewhat unevenly thick, apex club-shaped, covered with microtrichia. Tegumen hood-shaped. Phallus short, drop-shaped, laterally with narrow extensions. Valvae asymmetrical, short and straight, dorsally with subbasal triangular lobes, subapically with small transverse flaps, distally setose; right valva basally with complex heavily sclerotised lobe. Saccus rectangular, broad. Sternum VIII hexagonal basally, medioposteriorly with large U-shaped depression, posterior shanks somewhat asymmetrical; mediolaterally extended as small flaps at both sides, attached to two long and narrow medio-anterior apodemes. Tergum VIII narrow, tongue-shaped, lateral and posterior margins folded and furnished with ~ twelve long heavily sclerotised spiniform setae; anteriorly with two long and narrow diverging apodemes.

####### Etymology.

Latinised adjective in the nominative singular. The species name refers to the country in which the taxon was discovered.

####### Distribution.

Central Chile.

####### Habitat.

The habitat is a shrubby riverside spot with sparse vegetation in the Andes foothills.

####### Genetic data.

BIN: BOLD:ADZ5419 (*n* = 1 from Chile). Nearest neighbour: *Landryia* JFL138 from USA: California (BIN: BOLD:AAE6120, 6.18%).

####### Remarks.

Female unknown. Based on COI maximum likelihood phylogeny, the South American taxa *ankylosauroides* and *chilensis* group inside a large clade, whose taxa are classified in *Landryia* on BOLD (Suppl. material 2). However, *ankylosauroides* and *chilensis* do not have the diagnostic morphological characters of *Landryia*, such as greatly enlarged bulbus ejaculatorius (unless accidentally removed during dissection) in the male genitalia and the pincer-like projections on caudal margin of female sternum VII (Landry 1991). Also, male sternum VIII of *ankylosauroides* and *chilensis* are distinct with their spiniform setae and long apodemes, but such are not present in North American *Landryia* (Landry 1991). We therefore classified these two taxa in *Landryia* (incertae sedis), highlighting the need for further research.

##### *Scythris* Hübner, 1825

**The *directiphallella* species group**

Distal arm of gnathos and phallus long and slim. Ventral margin of valva often with large extension. Male sternum VIII pentagonal with distinct and sharp posterior shanks. Species included: *directiphallella*, *furciphallella*, *manchaoensis*, *salinasgrandensis*, *angustivalvella*, *zeugmatica*.

Male genitalia of *directiphallella* species group resemble the African *Haploscythris*, particularly the bilobed uncus, divided valva in several species and V-shaped anterior margin of vinculum (compare against illustrations in Bengtsson 2014). More data are needed to confirm or reject potential *Haploscythris* association.

###### ﻿ Scythris directiphallella

Taxon classificationAnimaliaLepidopteraScythrididae

Nupponen sp. nov.

6ACCFE25-30C7-51BC-8426-6E5F96FC3F46

http://zoobank.org/24086F7E-BD7F-400B-B3D0-8D165AAEB9B6

Figs 12 , 44

####### Type material.

***Holotype*.** Argentina • ♂; prov. Santiago del Estero, Pozo Honda village S, by salt lake; 27°17.2'S, 64°28.0'W; 260 m a.s.l.; 19 Sep. 2017; K. Nupponen & R. Haverinen leg.; [BOLD sample ID] KN01052; [genitalia slide] K. Nupponen prep. no. 3/28 Dec. 2019; coll. NUPP (MZH).

***Paratype*.** Argentina • 1 ♂; same data as for holotype; coll. NUPP.

####### Diagnosis.

Wings grey, impossible to separate externally from *S.furciphallella*. The male genitalia of *S.directiphallella* are by having narrow valvae with a ventral thorn-like process apically, a straight phallus and pincer-like extensions on posterior margin of male sternite VIII.

####### Description.

Wingspan 9–10.5 mm. Head, collar, neck tuft, haustellum, tegula and thorax grey. Scape grey, ventrally mixed with dirty white; pecten grey and as long as diameter of scape. Flagellum fuscous, 0.7 × length of forewing, ciliate, sensillae ~ 1/2 as long as diameter of flagellum. Labial palp: palpomere I dirty white, palpomeres II and III fuscous mixed with dirty white. Legs fuscous, more or less suffused with dirty white. Abdomen dorsally pale grey, ventrally a little paler, anal tuft cream. Forewing grey, over the wing sparsely scattered dark fuscous scales. Hindwing pale fuscous.

***Male genitalia*.** Gnathos (homology interpretation of gnathos and uncus based on Landry (1991)) base broad, weakly sclerotised belt; distally long, slender and bent downwards, tip pointed. Uncus bilobed plate, posterior shanks subapically with small nipple-like extensions. Tegumen elongated hood, dorsally widely open. Phallus 0.75 × length of valva, straight, tip pointed. Valva long and narrow; apex setose and slightly incurved; apically with robust ventral thorn-like process. Saccus short, triangular. Juxta narrow, 0.8 × length of phallus. Sternum VIII pentagonal; posteriorly bifurcate, shanks short, bent inwards with tips pointed. Tergum VIII trapezoid, anterior margin widely concave and weakly sclerotised, posterior margin convex.

####### Etymology.

Diminutive noun in apposition. The species name refers to the straight phallus of the male, which is a diagnostic character of the species.

####### Distribution.

NW Argentina.

####### Habitat.

The collecting site is a dry, shrubby area near a salt lake shore (Fig. 77).

####### Genetic data.

BIN: BOLD:ADY7318 (*n* = 2 from Argentina). Nearest neighbour: *Scythrissalinasgrandensis* Nupponen, sp. nov. (BIN: BOLD:ADY7738, 4.49%).

####### Remarks.

Female unknown. Based on COI maximum likelihood phylogeny, the South American taxa *salinasgrandensis*, *furciphallella*, *manchaoensis*, *angustivalvella*, and *directiphallella* group together, associating within a clade, whose taxa are classified in apparently non-monophyletic *Scythris* on BOLD (Suppl. material 2). We classify these taxa in *Scythris*.

###### ﻿ Scythris furciphallella

Taxon classificationAnimaliaLepidopteraScythrididae

Nupponen sp. nov.

D509CCD6-232E-5BA5-86E0-648D4F4AD871

http://zoobank.org/D8D83C42-B864-4ECD-85A8-90E0F26587CA

Figs 13 , 45 , 66

####### Type material.

***Holotype*.** Argentina • ♂; prov. Cordoba, Salinas Grandes SE shore; 29°50.5'S, 64°40.2'W; 185 m a.s.l.; 24 Sep. 2017; K. Nupponen & R. Haverinen leg.; [BOLD sample ID] KN01053; [genitalia slide] K. Nupponen prep. no. 2/16 Dec. 2019; coll. NUPP (MZH).

***Paratypes*.** Argentina • 6 ♂, 2 ♀; same data as for holotype; [genitalia slides] K. Nupponen prep. no. 3/16-XII-2019 ♀, 3/13-I-2019 ♂; coll. NUPP; • 1 ♂; prov. La Rioja, Andes Mts., Sierra de Famatina, Famatina village 15 km NNW; 28°46.4'S, 67°35.0'W; 2085 m a.s.l.; 27 Jan. 2017; K. Nupponen & R. Haverinen leg.; [BOLD sample ID] KN01050; [genitalia slide] K. Nupponen prep. no. 1/17 Dec. 2019; coll. NUPP.

####### Diagnosis.

A grey species, externally indistinguishable from *S.directiphallella*. In the male genitalia, a posteriorly bifurcate phallus and large backwards directed ventral lobes of the valvae are diagnostic.

####### Description.

Wingspan 9.5–10.5 mm. Head, collar, neck tuft, haustellum, tegula and thorax grey. Scape grey, ventrally mixed with cream; pecten grey and longer than diameter of scape. Flagellum fuscous, 0.7 × length of forewing, ciliate, sensillae ~ 0.8 × as long as diameter of flagellum. Labial palp: palpomere I dirty white, palpomeres II and III fuscous mixed with dirty white. Legs fuscous, more or less suffused with dirty white. Abdomen fuscous, ventrally paler, anal tuft ventrally cream. Forewing grey, wing irrorated with black scales; in some specimens’ very indistinct whitish streak in fold at basal 1/3. Hindwing pale fuscous.

***Male genitalia*.** Gnathos basally semi-circular; distally long, slender and bent downwards. Uncus rectangular plate, medioposteriorly with small indentation. Tegumen hood-shaped. Phallus 0.65 × length of valva, bent, apex bifurcate, slender branch twice longer than the other. Valva long and narrow, distally spatular; ventral margins subapically with huge, anteriorly-directed, slightly asymmetrical lobes. Saccus short, labiate. Juxta narrow, 0.5 × length of phallus. Sternum VIII pentagonal, paired posterior projections diverging, straight, tips pointed; anterior margin slightly concave. Tergum VIII trapezoid, elongated, posteriorly round and setose, anteriorly incurved.

***Female genitalia*.** Sterigma long and straight, rather stout, at 0.65 a little broadened, terminal 1/3 sclerotised, tip blunt. Ostium round, margins sclerotised, situated at 0.65 of sterigma. Sternum VII rectangular, 1.35 × wider than high, posterior margin medially incurved, anterior margin concave and sclerotised. Apophyses anteriores 0.55 × length of apophyses posteriores.

####### Etymology.

Diminutive noun in apposition. The species name refers to a bifurcate phallus of the male.

####### Distribution.

NW Argentina.

####### Habitat.

The collecting site is a shore of a large salt lake, in the edge between dry bushy area and low saline vegetation (Fig. 75).

####### Genetic data.

BIN: BOLD:ADY9699 (*n* = 2 from Argentina). The two barcode sequences are 0.96% distant. Nearest neighbour: *Scythrissalinasgrandensis* Nupponen, sp. nov. (BIN: BOLD:ADY7738, 4.49%).

####### Remarks.

Based on COI maximum likelihood phylogeny, South American taxa *salinasgrandensis*, *furciphallella*, *manchaoensis*, *angustivalvella* and *directiphallella* group together, associating within a clade, whose taxa are classified in apparently non-monophyletic *Scythris* on BOLD (Suppl. material 2). We classify these taxa in *Scythris*.

###### ﻿ Scythris manchaoensis

Taxon classificationAnimaliaLepidopteraScythrididae

Nupponen sp. nov.

4A51A4CE-79A2-5623-8581-6571C3E4B86F

http://zoobank.org/F426C532-CE83-4009-9BE0-2946412EB563

Figs 14 , 46

####### Type material.

***Holotype*.** Argentina • ♂; prov. Catamarca, Sierra de Manchao; 28°43.6'S, 66°21.1'W; 1190 m a.s.l.; 23 Sep. 2017; K. Nupponen & R. Haverinen leg.; [BOLD sample ID] KN01032; [genitalia slide] K. Nupponen prep. no. 1/11 Dec. 2019; coll. NUPP (MZH).

####### Diagnosis.

A fuscous species, externally similar to *S.salinasgrandensis*, but distinguished by the fringes being the same colour as the forewing surface (distinctly darker in *S.salinasgrandensis*) and a small spot at cell end (lacking in *S.salinasgrandensis*). The male genitalia of *S.manchaoensis* resemble those of *S.angustivalvella*, but differ in the distally broader valva and the sigmoid phallus, short and converging appendices on posterior margin of sternum VIII (narrower valva, arched phallus, long and diverging appendices in *S.angustivalvella*).

####### Description.

Wingspan 15.5 mm. Head, collar, neck tuft, haustellum, tegula and thorax fuscous, same colour as forewing. Scape fuscous; pecten paler and longer than diameter of scape. Flagellum fuscous, 0.65 × length of forewing, in male ciliate, sensillae as long as diameter of flagellum. Labial palp: palpomere I pale fuscous white; palpomere II: inner surface dirty white fuscous, otherwise fuscous with faintly scattered dirty white; palpomere III pale fuscous with distal 1/2 suffused faintly darker. Legs fuscous, mixed with dirty white, more so in hind legs. Abdomen dorsally pale fuscous, ventrally dirty whitish fuscous. Forewing fuscous with sparsely scattered blackish scales, indistinct dark spot at cell end. Hindwing fuscous, slightly paler than forewing.

***Male genitalia*.** Uncus bilobed plate, tips of posterior lobes bent ventrad and pointed. Gnathos base hood-like, rather weakly sclerotised; distal arm long and slender, bent 90° at basal 1/2, tip bent downwards and pointed. Phallus slender, shallowly sigmoid, 0.8 × length of gnathos arm, tip pointed. Valva straight and broad at basal 0.6; distal 0.4 narrow and bent, tip widened, spatular and setose; ventrally at middle long, robust, incurved, horn-like process. Saccus short, triangular. Juxta narrow, elongate, 1.15 × length of phallus. Sternum VIII pentagonal, posteriorly bifurcate, shanks short and converging; anterior corners widened, anterior margin incurved and somewhat sclerotised. Tergum VIII triangular, posteriorly elongate with blunt tip, anterior margin wide, concave.

####### Etymology.

Latinised adjective in the nominative singular. The species is named after the type locality, in the Manchao range of the Andes.

####### Distribution.

NW Argentina.

####### Habitat.

The collecting site is a dry and xerothermic rocky slope with low vegetation and sparse shrubs (Fig. 76).

####### Genetic data.

BIN: BOLD:ADY8793 (*n* = 1 from Argentina). Nearest neighbour: *Scythrisangustivalvella* Nupponen, sp. nov. (BIN: BOLD:ADY8789, 2.75%). *Scythrissalinasgrandensis*, whose male is unknown, is externally similar, and its barcode differs by 5.62%.

####### Remarks.

Female unknown. Based on COI maximum likelihood phylogeny, South American taxa *salinasgrandensis*, *furciphallella*, *manchaoensis*, *angustivalvella*, and *directiphallella* group together, associating within a clade, whose taxa are classified in apparently non-monophyletic *Scythris* on BOLD (Suppl. material 2). We classify these taxa in *Scythris*. The male genitalia of *angustivalvella* and *manchaoensis* are similar to *S.zhakovi* Bidzilya & Budashkin, 2017 from Ukraine (Bidzilya et al. 2017).

###### ﻿ Scythris salinasgrandensis

Taxon classificationAnimaliaLepidopteraScythrididae

Nupponen sp. nov.

12FA4212-95E3-5340-8B67-842A223F066D

http://zoobank.org/92E7893B-FB97-4B21-8577-5626BB509745

Figs 15 , 67

####### Type material.

***Holotype*.** Argentina • ♀; prov. Cordoba, Salinas Grandes SE shore; 29°50.5'S, 64°40.2'W; 185 m a.s.l.; 24 Sep. 2017; K. Nupponen & R. Haverinen leg.; [BOLD sample ID] KN01033; [genitalia slide] K. Nupponen prep. no. 3/11 Dec. 2019; coll. NUPP (MZH).

***Paratypes*.** Argentina • 10 ♀; same data as for holotype; [BOLD sample ID] KN01034; coll. NUPP; • 1 ♀; same data as for holotype except collecting date; 13 Sep. 2017; coll. NUPP.

####### Diagnosis.

Wings pale grey, finely peppered with brown fuscous species, externally easily mixed with *S.manchaoensis*, but separated by fringes being distinctly darker than forewing (same colour as forewing in *S.manchaoensis*) and absence of small spot at cell end (present in *S.manchaoensis*). In the female genitalia, a large and distinctly defined oval sterigma is diagnostic.

####### Description.

Wingspan 15–18 mm. Head, collar, neck tuft, haustellum, tegula and thorax fuscous, same colour as forewing, head and haustellum mixed with dirty white. Scape fuscous except ventral surface and pecten whitish grey, pectin longer than diameter of scape. Flagellum fuscous, 0.6 × length of forewing. Labial palp: palpomere I pale dirty white; palpomeres II and III: upper surface dirty white, otherwise fuscous mixed with a few dirty white scales. Legs fuscous, mixed with dirty white. Abdomen dorsally pale fuscous, ventrally dirty white. Forewing pale grey, finely peppered with brown, fringes darker than wing. Hindwing slightly paler than forewing.

***Female genitalia*.** Sterigma oval ring with sclerotised margin, anteriorly with quadrangular sclerotised extension. Ostium situated anteriorly in ring. Sternum VII quadrangular; posterior margin medially incurved, anterior margin sclerotised. Apophyses anteriores 0.5 × length of apophyses posteriores.

####### Etymology.

Latinised adjective in the nominative singular. The species is named after the type locality, the Salinas Grandes salt lake.

####### Distribution.

NW Argentina.

####### Habitat.

The collecting site is the shore of a large salt lake, in the zone between a dry shrubby area and low halophytic vegetation.

####### Genetic data.

BIN: BOLD:ADY7738 (*n* = 2 from Argentina). Nearest neighbour: *Scythrisfurciphallella* Nupponen, sp. nov. (BIN: BOLD:ADY9699, 4.49%). *Scythrismanchaoensis* is externally similar, and its barcode differs by 5.62%.

####### Remarks.

Male unknown. Based on COI maximum likelihood phylogeny, South American taxa *salinasgrandensis*, *furciphallella*, *manchaoensis*, *angustivalvella* and *directiphallella* group together, associating within a clade, whose taxa are classified in apparently non-monophyletic *Scythris* on BOLD (Suppl. material 2). We classify these taxa in *Scythris*.

###### ﻿ Scythris angustivalvella

Taxon classificationAnimaliaLepidopteraScythrididae

Nupponen sp. nov.

0164964D-DEE7-5B91-91A1-58AA2C27B504

http://zoobank.org/A3BEA5B6-87AB-4846-B264-F06E0A51F110

Figs 16 , 47

####### Type material.

***Holotype*.** Argentina • ♂; prov. Santiago del Estero, Pozo Honda village S, by salt lake; 27°17.2'S, 64°28.0'W; 260 m a.s.l.; 19 Sep. 2017; K. Nupponen & R. Haverinen leg.; [BOLD sample ID] KN01051; [genitalia slide] K. Nupponen prep. no. 4/16 Dec. 2019; coll. NUPP (MZH).

####### Diagnosis.

Both fore- and hindwings are fuscous. The male genitalia of *S.angustivalvella* resemble those of *S.manchaoensis*, but differ by the distally narrower valva and arched phallus (sigmoid in *S.manchaoensis*), as well as in details of sternum VIII (posteromedial prongs long and diverging in *angustivalvella*, short with apices converging in *manchaoensis*) and details of tergum VIII (posterior margin indented in *angustivalvella*, with medial extension rounded in *manchaoensis*).

####### Description.

Wingspan 11 mm. Head, collar, neck tuft, haustellum, tegula and thorax fuscous, with scattered dirty white scales. Scape dorsally fuscous, ventrally dirty white, pecten dirty white and longer than diameter of scape. Flagellum brown, 0.7 × length of forewing, ciliate, sensillae as long as diameter of flagellum. Labial palp: palpomere I white; palpomeres II and III: lower surface dark brown, otherwise dirty white. Legs dirty white, tibiae and tarsi mixed with brown. Abdomen dorsally fuscous, ventrally dirty white. Forewing fuscous; narrow indistinct whitish streak in fold from base to midwing; small dark brown spot at cell end. Hindwing fuscous.

***Male genitalia*.** Uncus a bifurcate plate, tips of posterior lobes blunt. Gnathos base a broad belt; distal arm long, slightly upcurved, basal 1/2 tapered, posterior 1/2 slender, tip pointed. Tegumen broader than high, margins reinforced. Phallus 0.65 × length of valva, slim and arched, tip pointed. Valva long and narrow; basal 1/2 tapered, distal 1/2 slender and incurved, apex spatular; a robust, downcurved horn-like projection ventrally at 0.5. Saccus short, triangular. Juxta narrow, 0.55 × length of phallus. Sternum VIII pentagonal, posteromedial prongs divergent, straight, tips pointed; anterior margin slightly concave. Tergum VIII trapezoid, anterior margin widely incurved, posterior margin slightly indented.

####### Etymology.

Diminutive noun in apposition. The species name refers to the narrow valvae in the male genitalia.

####### Distribution.

NW Argentina.

####### Habitat.

The collecting site is a dry shrubby area near a salt lake shore (Fig. 77).

####### Genetic data.

BIN: BOLD:ADY8789 (*n* = 1 from Argentina). Nearest neighbour: *Scythrismanchaoensis* Nupponen, sp. nov. (BIN: BOLD:ADY8793, 2.57%).

####### Remarks.

Female unknown. Based on COI maximum likelihood phylogeny, South American taxa *salinasgrandensis*, *furciphallella*, *manchaoensis*, *angustivalvella*, and *directiphallella* group together, associating within a clade, whose taxa are classified in apparently non-monophyletic *Scythris* on BOLD (Suppl. material 2). We classify these taxa in *Scythris*. The male genitalia of *angustivalvella* and *manchaoensis* are similar to *S.zhakovi* Bidzilya & Budashkin, 2017 from Ukraine (Bidzilya et al. 2017).

###### ﻿ Scythris zeugmatica

Taxon classificationAnimaliaLepidopteraScythrididae

Meyrick, 1931

AE9DFDF7-4220-5DA0-ACBD-74C787FA2FA1

Figs 17 , 48

Scythris zeugmatica Meyrick, 1931. Exotic Microlepidoptera 4 (part 6): 179.

####### Material examined.

***Holotype*** (fixed by monotypy, Art. 73.1.2 (ICZN 2000). Brazil • ♂; Santarem; 8.19.; Parish leg.; [genitalia slide] JFGC No. 8050; NHMUK ID 010922366; NHMUK slide ID 010316662; coll. NHMUK.

####### Diagnosis.

A small species (10 mm), externally resembles to some extent *S.zeugmatica* with similar whitish streak on forewing. *Scythriszeugmatica* is readily separated from the other described species by characters in the male genitalia, particularly by bilobed uncus, a peculiar vertical sclerotisation with lateral expansion (homology unclear), and broad, symmetrical valvae with a small subapical ventral tooth.

####### Description.

The original description is quoted: “Wingspan ♂ 10 mm. Head whitish. Palpi whitish, terminal joint suffused grey. Thorax bronzy-grey. Abdomen dark grey, beneath whitish-ochreous. Forewings elongate-lanceolate; rather dark purple-grey; a rather broad suffused yellow-whitish streak along fold throughout, crossed at its middle by a fasciate bar reaching dorsum but not reaching costa, beyond this attenuated and indistinct, but expanded into an oval spot on tornus, a somewhat inwards-oblique spot on costa towards apex rather beyond this: cilia grey. Hindwings 0.75, grey; cilia grey.”

***Male genitalia*.** Uncus bilobed, basally fused by narrow transverse sclerotisation. Gnathos base U-shaped. Tegumen hood-shaped, anterior margin medially deeply cleft. Ventrad of tegumen are situated two sclerotised, vertical structures (homologies are unclear): other rather straight with sharp apexes (Fig. 48 on left), other slightly longer, at middle triangularly extended (Fig. 48 on right). Valva broad and straight, dorsal margin at basal quarter somewhat folded; ventrally slightly broadened beyond middle, subapically with small triangular tooth. Saccus short, triangular, at base with small digitate process. Sternum VIII trapezoid, anterolaterally with small lobes, posteriorly with pair of stout parallel horn-like projections. Tergum VIII subrectangular, laterally concave, anterior margin sclerotised.

####### Distribution.

Brazil.

####### Remarks.

Female unknown. DNA barcode is not available yet for *S.zeugmatica*. We place *S.zeugmatica* in the *directiphallella* species group based on morphology. It has a similar long and slim gnathos, long and slim phallus, and male sternum VIII has sharp posterior shanks.

###### ﻿ Scythris caimancitoensis

Taxon classificationAnimaliaLepidopteraScythrididae

Nupponen sp. nov.

C5FE131B-032E-56CB-82E8-99599A321B6E

http://zoobank.org/BC28429D-6162-4356-865E-6C5DA7B1F9E9

Figs 18 , 49

####### Type material.

***Holotype*.** Argentina • ♂; prov. Jujuy, Rio San Francisco, by Caimancito village; 23°43.8'S, 64°36.3'W, 400 m a.s.l.; 18 Sep. 2017; K. Nupponen & R. Haverinen leg.; [BOLD sample ID] KN01037; [genitalia slide] K. Nupponen prep. no. 3/9 Dec. 2019; coll. NUPP (MZH).

####### Diagnosis.

Externally somewhat resembling *S.tibicina*, but distinguished by the more contrasty pattern and cream colour of the forewings. In the male genitalia, the strikingly long and blade-like valvae (which sticks out from the abdomen, see Fig. 18) and an elongate sternum VIII are diagnostic.

####### Description.

Wingspan 13.5 mm. Head brown, laterally paler. Collar and neck tuft mixed with various shades of brown and dirty white, tegula pale brown. Haustellum dirty white. Thorax dark brown. Scape dorsally dark brown, ventrally dirty cream; pecten dirty cream and longer than diameter of scape. Flagellum dark brown, 0.7 × length of forewing, ciliate, sensillae ~ 1/2 as long as diameter of flagellum. Labial palps white, except lower surface of palpomeres II and III dark brown. Legs dirty white, except tibia and tarsus of foreleg brown, and upper surface of hindleg tarsus with pale brown hair. Abdomen dorsally dark fuscous, ventrally white. Forewing dark brown, blackish at basal 1/2 of wing at costal and widely at dorsal areas; fold broadly cream, connected to dash of same colour at cell end, the latter extended to subapical area. Hindwing dark fuscous.

***Male genitalia*.** Uncus small and labiate. Gnathos reduced to small transverse flap. Tegumen hood-shaped. Phallus short, basally indented, distal 1/2 tapered, tip extended, bent and pointed. Valva very long, straight and of constant width, apically tapered and bent inwards, tip pointed; costal and dorsal margins sclerotised and setose. Saccus U-shaped, ca. as long as tegumen. Sternum VIII subrectangular, strongly elongated and narrow, deeply indented both postero- and anteromedially; anterior margin with two parallel triangular lobes; at anterior 1/3 of plate two longitudinal setose ridges. Tergum VIII small, rectangular, posterior margin widely concave, anterior corners extended.

####### Etymology.

Latinised adjective in the nominative singular. The species is named after the type locality, the village of Caimancito.

####### Distribution.

NW Argentina.

####### Habitat.

The collecting site is a dry river bed surrounded by forests and plantations. Plants of the family Amaranthaceae were common along the river banks (Fig. 79).

####### Genetic data.

Not obtained (specimen submitted to barcode analysis but the sample failed).

####### Remarks.

Female unknown. The male genitalia do not show affinities to other described American Scythrididae. The very large male genitalia is diagnostic for *Arotrura* Walsingham 1888, but *caimancitoensis* does not have the other diagnostic characters of that genus (Landry 1991). We classify *caimancitoensis* in *Scythris*, but the genus combination needs more research.

**Figures 12–17.**
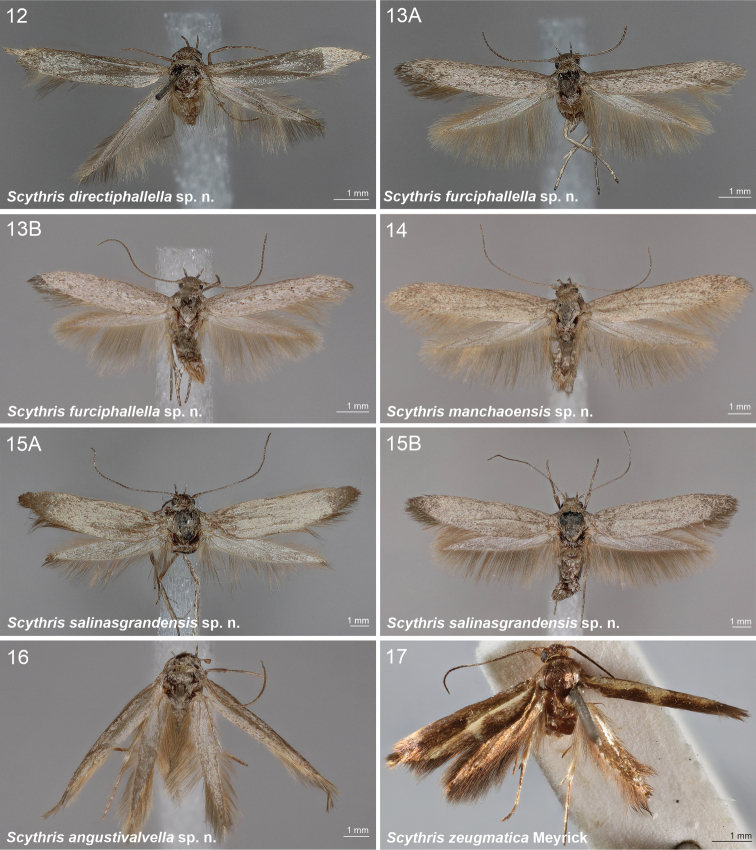
Scythrididae adults, genus *Scythris*. **12***S.directiphallella* Nupponen, sp. nov., male, holotype **13A***S.furciphallella* Nupponen sp. nov., male, holotype **13B***S.furciphallella* Nupponen sp. nov., male, paratype **14***S.manchaoensis* Nupponen, sp. nov., male holotype **15A***S.salinasgrandensis* Nupponen sp. nov., female, holotype **15B***S.salinasgrandensis* Nupponen sp. nov., female, paratype **16***S.angustivalvella* Nupponen sp. nov., male, holotype **17***S.zeugmatica* Meyrick, 1916, male, holotype.

###### ﻿ Scythris ejiciens

Taxon classificationAnimaliaLepidopteraScythrididae

Meyrick, 1928

4BD661FB-94C8-5515-9B31-C19C66F66716

Fig. 19

Scythris ejiciens Meyrick, 1928. Exotic Microlepidoptera, vol. 3 (part 13): 412.

####### Material examined.

***Holotype*** (fixed by monotypy, Art. 73.1.2 (ICZN 2000)). Peru • ♀; Cocapata; 12.000 feet a.s.l.; NHMUK ID 010922358; coll. NHMUK.

####### Diagnosis.

The abdomen of the type is missing. A small species (wingspan 9 mm). Externally *S.ejiciens* may be separated from the other described Neotropical *Scythris* by a distinct whitish-ochreous streak along the fold from base to the end of cell, followed by whitish-ochreous spot.

####### Description.

The original description is quoted: “Wingspan 9 mm ♀. Head, thorax rather dark purplish-fuscous. Palpi grey. Forewings rather dark purplish-fuscous; a whitish-ochreous streak along fold from base to beyond middle of wing; a roundish whitish-ochreous spot in disc at 0.75: cilia fuscous. Hindwings dark grey; cilia fuscous.”

####### Distribution.

Peru.

####### Remarks.

Male unknown. The type specimen of *S.ejiciens* lacks the abdomen and does not have a genital preparation label. Clarke (1965) reported “The abdomen is missing.”

###### ﻿ Scythris fluvialis

Taxon classificationAnimaliaLepidopteraScythrididae

Meyrick, 1916

D8C52B4C-0FB8-5B85-B58C-D8C3741364FD

Figs 20 , 50

Scythris fluvialis Meyrick, 1916. Exotic Microlepidoptera, vol. 2 (part 1): 15.

####### Material examined.

***Lectotype*.** Colombia • ♂; Cali; 500 feet a.s.l.; 5–14.; Parish leg.; [genitalia slide] JFGC No. 8052; NHMUK ID 010922359; NHMUK slide ID 010316664; coll. NHMUK.

***Paralectotype*.** Colombia • ♀; same data as for lectotype; coll. NHMUK.

####### Diagnosis.

*Scythrisfluvialis* and North American *S.trivinctella* (Zeller, 1873) and *S.ypsilon* Braun, 1920, in addition to five undescribed species, form a compact group, sharing twisted apex of the distal arm of the gnathos, terminating into a small, warped plate (Landry 1991). Posterior margin of male abdominal tergum VIII is elongated in *S.fluvialis*, bifurcate in *S.trivinctella* and distinctly concave with lateral setae in *S.ypsilon*. See Landry (1991) for further details.

####### Description.

The original description is quoted: “Wingspan 12–13 mm ♂, ♀. Head, palpi, and thorax dark bronzy-fuscous. Antennal ciliations of ♂ 0.5. Abdomen bronzy-fuscous, beneath in ♂ suffused with pale ochreous, in ♀ white except anal segment. Forewings lanceolate; dark violet-fuscous, towards costa and dorsum suffused with grey; a thick suffused ochreous-whitish streak from base of dorsum, curved upwards to above middle and returning to fold before middle of wing, where it joins an ochreous tinged patch extending along dorsum to tornus; a thick ochreous-whitish streak from 0.2 of costa to fold parallel to termen, with a , with a projection on posterior edge in middle, tending to connect with a whitish mark on termen above tornus; some ochreous tinge towards termen above this; in ♂ specimen an ochreous-whitish mark at apex: cilia rather dark violet-fuscous. Hindwings with 4 and 5 separate; dark fuscous; cilia dark grey.”

***Male genitalia*.** Uncus trapezoid plate. Gnathos base broad belt, dorsally a semi-circular extension covered by minute thorns; distal arm long, sigmoid, tip pointed with small flap. Tegumen hood-shaped. Phallus 1/2 length of valva, basal 2/3 straight and of constant width, distal 1/3 bent ventrally and tapered. Valva long and narrow, distal 1/2 weakly broadened dorsally, tip round and setose. Saccus 0.6 ×as long as valva, broad. Sternum VIII pentagonal plate basally, laterally broadened, apex elongated. Tergum VIII pentagonal plate, posterior extension long and digitate, anterior margin concave, U-shaped.

***Female genitalia*.** Not dissected.

####### Distribution.

Colombia.

####### Remarks.

We leave *fluvialis* in *Scythris*, more precisely next to *S.trivinctella* and *S.ypisilon*, following the diagnostic characters provided by Landry (1991).

###### ﻿ Scythris inanima

Taxon classificationAnimaliaLepidopteraScythrididae

Meyrick, 1916

81E1BCEB-B9BB-530F-BD00-9ED341F5FBBA

Figs 21 , 51

Scythris inanima Meyrick, 1916. Exotic Microlepidoptera, vol. 2 (part 1): 13.

####### Material examined.

***Holotype*.** Peru • ♂; Huancayo; 10650 feet a.s.l.; i.7.14.; Parish leg.; [genitalia slide] JFGC No. 8051; NHMUK ID 010922361; NHMUK slide ID 010316663M coll. NHMUK.

####### Diagnosis.

Forewings bronzy-grey without distinctive external features. Genitalia dissection is required for recognition. *Scythrisinanima* is readily separated from the other described species by details of the male genitalia: wide, inwards curved, pointed and asymmetrical valvae, a tubular phallus bent at 90° angle in basal 1/3, and “anchor-shaped” abdominal segment VIII with two curved projections are unique among the examined materials.

####### Description.

The original description is quoted: “Wingspan 10 mm ♂. Head, palpi, thorax, and abdomen light bronzy-grey. Antennal ciliations 1. Forewings lanceolate; bronzy-grey, somewhat darker-springled in disc: cilia greyish.”

***Male genitalia*.** Uncus trapezoid sclerotised plate. Gnathos base broad belt; distal arm short, robust, directed upwards and heavily sclerotised. Tegumen hood-shaped. Phallus slim, a little longer than valva, basal 1/3 bent at 90° angle, distally straight. Valvae asymmetrical, left broader and shorter; basal 2/3 broad, distal 1/3 tapered and bent inwards, tip more or less pointed. Saccus arched, short. Sternum VIII pentagonal, anterior margin broadly reinforced, mediolaterally somewhat extended. Tergum VIII consists of two laterally arched sclerotised belts; medioposterior portion triangular with backwards directed lateral extensions.

####### Distribution.

Peru.

####### Remarks.

Female unknown.

###### ﻿ Scythris lequetepequensis

Taxon classificationAnimaliaLepidopteraScythrididae

Nupponen sp. nov.

05FD0F08-5DD2-504A-A759-D7E187321014

http://zoobank.org/3062C052-50C6-441C-B222-0B19036C60EF

Figs 22 , 52

####### Type material.

***Holotype*.** Peru • ♂; prov. La Libertad, Lequetepeque River, near El Huabal village; 7°16.9'S, 79°18.2'W; 200 m a.s.l.; 1 Feb. 2019; K. Nupponen & R. Haverinen leg.; [BOLD sample ID] KN01074; [genitalia slide] K. Nupponen prep. no. 2/8 Dec. 2019; coll. NUPP (MZH).

***Paratype*.** Peru • ♂; prov. Cajamarca, Lequetepeque River, near Chilete village; 7°12.9'S, 78°45.3'W; 980 m a.s.l.; 4 Feb. 2019; K. Nupponen & R. Haverinen leg.; coll. NUPP.

####### Diagnosis.

Rather reliably determined externally by pale brown forewings with indefinite paler areas at midwing, a dark brown subapical spot and fringe under tornus being darker than those at apical area. In the male genitalia of *S.lequetepequensis*, the gnathos with massive base and dorsally expanded pouch, and a transverse and sclerotised arched sclerite at tergum VIII are diagnostic.

####### Description.

Wingspan 13 mm. Head, collar, neck tuft, tegula and thorax pale brown. Few whitish brown scales around eye; thorax posteriorly edged by white scales. Haustellum white. Scape dorsally pale brown, ventrally paler, pecten longer than diameter of scape. Flagellum dark brown, 0.75 × length of forewing, ciliate, sensillae ~ 1/2 as long as diameter of flagellum. Labial palp: palpomere I white; palpomeres II and III with lower surface brown, otherwise whitish brown. Legs pale cream, upper surfaces of tibiae and tarsi mixed with pale brown in mid- and hindlegs, and darker brown in foreleg. Abdomen dorsally fuscous, ventrally white. Forewing pale brown, middle part of wing indefinite paler than costal and dorsal areas; at cell end whitish cream blotch, subapically small dark brown spot; fringe under tornus darker than those at apical area. Hindwing dark fuscous, darker than forewing.

***Male genitalia*.** Uncus bifurcate plate, posterior lobes broad, rounded. Gnathos as long as phallus; base massive with dorsally expanded pouch; distal arm tapered, tip with T-shaped hook. Tegumen round hood. Phallus 0.7 × length of valva, medially bent, distal 1/2 tapered, tip pointed. Valva straight, distal 1/2 dorsally somewhat widened and setose, apex round. Saccus labiate, ~ 1/3 as long as valva. Sternum VIII trapezoid, medioposteriorly shallowly indented; anterior margin concave and reinforced. Tergum VIII tongue-shaped, lateral and posterior margins folded, anterior margin medially with V-shaped indentation; subposteriorly with transverse and arched ridge covered by minute spines.

####### Etymology.

Latinised adjective in the nominative singular. The species is named after the type locality, valley of the River Lequetepeque.

####### Distribution.

Peru.

####### Habitat.

The collecting locality is a moist riverside meadow (Fig. 80).

####### Genetic data.

Not obtained (specimen submitted to barcode analysis but the sample failed).

####### Remarks.

Female unknown. We classify taxon *lequetepequensis* in genus *Scythris*, based on the somewhat similar male genitalia between *S.lequetepequensis* and North American (Landry 1991) and African (Bengtsson 2014) species such as *S.mixaula* Meyrick, 1916 from South-West USA and *S.cretiflua* Meyrick, 1913 from South Africa. These include for instance massive gnathos, horizontally narrow point of articulation between tegumen and valva, and symmetrical and apically setose valva.

###### ﻿ Scythris plocogastra

Taxon classificationAnimaliaLepidopteraScythrididae

Meyrick, 1931

0C5A3ECD-5D94-5812-B2AF-338C50A7F8CB

Figs 23 , 68

Scythris plocogastra Meyrick, 1931. Zoological Journal of the Linnean Society 37: 282.

####### Material examined.

***Holotype*.** Paraguay • ♀; Chaco: Makthlawaiya; • GSC [G. S. Carter]; 5.27.; [genitalia slide] JFGC No. 8063; NHMUK ID 010922365; NHMUK slide ID 010316672; coll. NHMUK.

####### Diagnosis.

Wings rather uniform purplish-grey, speckled weakly with white, without distinguishing external features. Genitalia examination is necessary for confident determination. In the female genitalia, the candleflame-shaped sterigma is characteristic.

####### Description.

The original description is quoted: “Wingspan ♀ 12 mm. Head and thorax purplish-grey, irregularly mixed white. Palpi dark grey sprinkled white, base white. Abdomen blackish, thickly strewn with white hair-scales, anal segment whitish, ventral surface wholly suffused white, apex ochreous-yellow. Forewings purplish-grey speckled dark fuscous and sprinkled whitish: cilia pale grey; cilia grey.”

***Female genitalia*.** Sterigma distinct, candleflame-shaped plate; posterior apex melanised; anterior margin weakly concave. Sternum VII rectangular, 1.4 × as high as wide; posterior margin with medial incision. Apophyses anteriores 0.7 × length of apophyses posteriores.

####### Distribution.

Paraguay.

####### Remarks.

Male unknown.

###### ﻿ Scythris tibicina

Taxon classificationAnimaliaLepidopteraScythrididae

Meyrick, 1916

403821CD-7658-5DA2-8489-13DDC6E8F2D3

Figs 24 , 53 , 69

Scythris tibicina Meyrick, 1916. Exotic Microlepidoptera, vol. 2 (part 1): 12.

####### Material examined.

***Lectotype*.** Peru • ♂; Chosica; 2800 feet a.s.l.; 7.14.; Parish leg.; [genitalia slide] JFGC No. 8053; NHMUK ID 010922365; NHMUK slide ID 010316665; coll. NHMUK.

***Paralectotype*.** Peru • 11 exx.; same data as for lectotype; coll. NHMUK.

####### Other material.

Peru • 1 ♂; prov. Ancash, near Huanchay village; 10°30.4'S, 77°25.5'W; 1520 m a.s.l.; 5 Feb. 2019; K. Nupponen & R. Haverinen leg.; [BOLD sample ID] KN01075; [genitalia slide] K. Nupponen prep. no. 5/11 Dec. 2019; coll. NUPP. • 2 ♂, 2 ♀; prov. Ancash, Fortaleza River, Raquia village 13 km SW; 10°13.1'S, 77°33.6'W; 1180 m a.s.l.; 31 Jan. 2019; K. Nupponen & R. Haverinen leg. [BOLD sample IDs] KN01076, KN01077; [genitalia slides] K. Nupponen prep. no. 1/18-XII-2019 ♀, 4/17-XII-2019 ♂; coll. NUPP.

####### Diagnosis.

Forewings with whitish streak on brownish background. Genitalia dissection is required for confident determination. The male genitalia are unmistakable, particularly the narrow, ventrally curved, hook-shaped gnathos; and phallus that bends at 90° angle; and densely bristled valvae. In the female genitalia, a crater-shaped margin of sterigma, adjoined by needle-like sclerotisation, are diagnostic.

####### Description.

The original description is quoted: “Wingspan 12–13 mm ♂, ♀. Head ochreous-grey more or less mixed with white. Palpi grey, suffused with white internally and at apex of second joint. Antennal ciliations of ♂ 1. Thorax ochreous-grey partially mixed with whitish. Abdomen light grey, anal tuft pale ochreous, ventral surface whitish. Forewings lanceolate; light grey: a double finely separated or united median whitish streak, from base, upper portion extending to about middle, lower to 0.33, both more or less enlarged into suffused spots posteriorly; an irregular elongate undefined spot of whitish suffusion in disc at 0.66; each of these whitish markings followed by a few indistinct dark fuscous scales, representing the stigmata: cilia grey, base mixed with whitish. Hindwings with 4 and 5 separate; grey; cilia grey.”

***Male genitalia*.** Uncus posterolaterally extended trapezoid plate, margin concave medially. Gnathos asymmetrical, basally channel-like, apex spoon-shaped. Distal arm of gnathos thin, curved ventrally, hook-shaped. Tegumen hood-shaped. Phallus 0.6 × length of valva; basal 2/3 straight, then bent at 90° angle, distal 1/3 slender and straight, tip pointed. Valva long and narrow, bent at 0.4 length, distal portion straight and setose; ventrally at middle sub-oval bristled extension. Vinculum arched, short. Sternum VIII large trapezoid plate, medioposteriorly with small V-shaped indentation, laterally at 0.3 with anteriorly directed lobes. Tergum VIII small trapezoid plate.

***Female genitalia*.** Sterigma crater-shaped, twice as wide as high, adjoined by needle-like sclerotisation. Ostium situated at bottom of crater. Sternum VII semi-circular, medioposteriorly with small concave notch. Apophyses anteriores 0.25 × length of apophyses posteriores.

####### Distribution.

Peru.

####### Habitat.

Adults were collected in moist riverside meadows.

####### Genetic data.

BIN: BOLD:ADZ4797 (*n* = 3 from Peru). Genetically homogenous, variation 0%. Nearest neighbour: Unidentified Scythrididae from Argentina (BIN: BOLD:ACY3332, 6.54%), see Suppl. material 2.

####### Remarks.

Based on COI maximum likelihood phylogeny, taxa *tibicina* and *sanfranciscoensis* group together, associating with other Central and South American taxa, classified in apparently non-monophyletic *Scythris* on BOLD (Suppl. material 2). With regard to *tibicina*, the male genitalia are similar to *S.mixaula* Meyrick, 1916 from California, Texas and Montana, sharing for instance narrow and setose valva, spear-shaped uncus (termed distal arm of gnathos in Landry (1991)) and mediodorsally convex vinculum. We have interpreted the mediodorsal structure as uncus (gnathos in Landry (1991) and the sclerotised structure on its ventral side as gnathos. We justify this interpretation by the origin of the narrow and ventrally curved process, which originates from the cup-shaped apex of gnathos. See Fig. 53, which shows the origin of the structure in lateral view. We classify *tibicina* and *sanfranciscoensis* in *Scythris*.

**Figures 18–24A.**
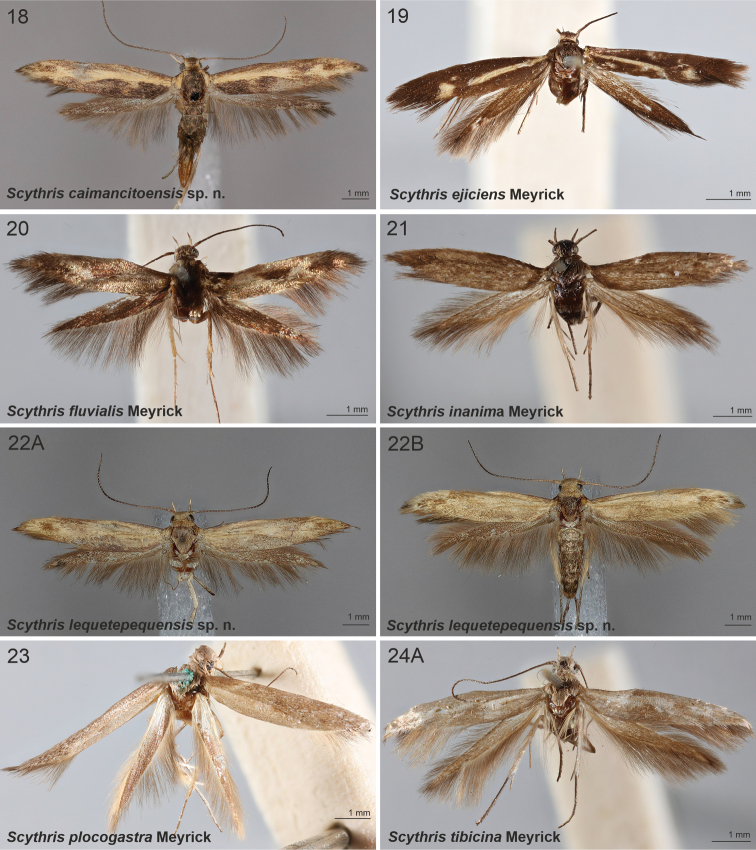
Scythrididae adults, genus *Scythris***18***S.caimancitoensis* Nupponen, sp. nov., male, holotype **19***S.ejiciens* Meyrick, 1928, male, holotype **20***S.fluvialis* Meyrick, 1916, male, lectotype **21***S.inanima* Meyrick, 1916, male, holotype **22A***S.lequetepequensis* Nupponen sp. nov., male, holotype **22B***S.lequetepequensis* Nupponen sp. nov., male, paratype **23***S.plocogastra* Meyrick, 1931, female, holotype **24A***S.tibicina* Meyrick, 1916, male, lectotype.

###### ﻿ Scythris sanfranciscoensis

Taxon classificationAnimaliaLepidopteraScythrididae

Nupponen sp. nov.

3D3EB34A-5355-51C3-8DDA-F7CE07CC4AD9

http://zoobank.org/BDCD7172-CA15-49CD-9A47-D8C2D3FB9527

Figs 25 , 54 , 70

####### Type material.

***Holotype*.** Argentina • ♂; prov. Jujuy, Rio San Francisco, by Caimancito village; 23°43.8'S, 64°36.3'W; 400 m a.s.l.; 18 Sep. 2017; K. Nupponen & R. Haverinen leg.; [BOLD sample ID] KN01036; [genitalia slide] K. Nupponen prep. no. 2/10 Dec. 2019; coll. NUPP (MZH).

**Figures 24B–29.**
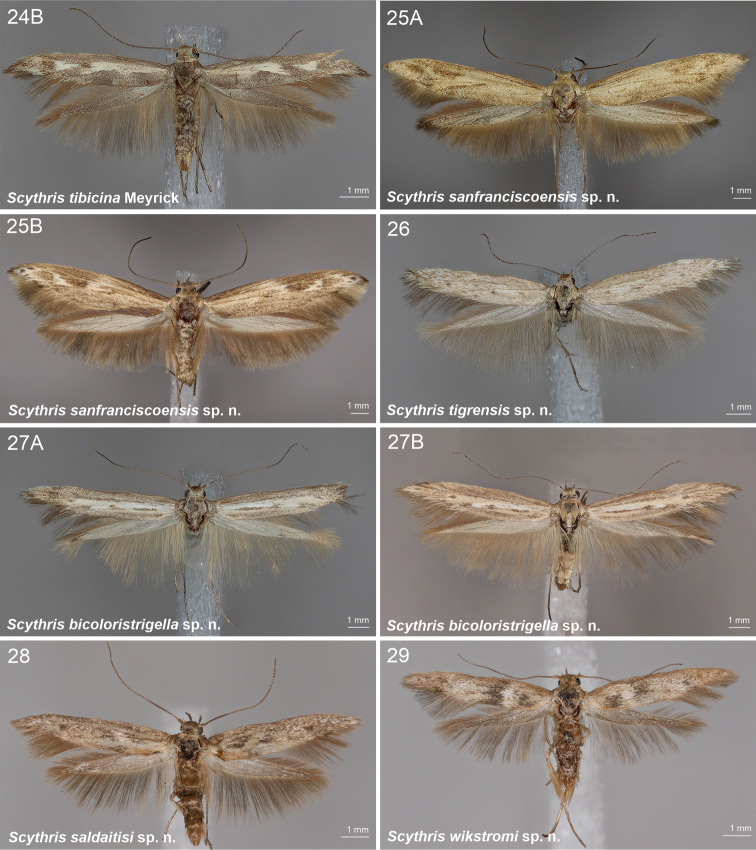
Scythrididae adults, genus *Scythris***24B***S.tibicina* Meyrick, 1916, male **25A***S.sanfranciscoensis* Nupponen sp. nov., male, holotype **25B***S.sanfriscoensis* Nupponen sp. nov., male, paratype **26***S.tigrensis* Nupponen, sp. nov., male, holotype **27A***S.bicoloristrigella* Nupponen sp. nov., genus combination incertae sedis, male, holotype **27B***S.bicoloristrigella* Nupponen sp. nov., genus combination incertae sedis, male, paratype **28***S.saldaitisi* Nupponen sp. nov., genus combination incertae sedis, male, holotype **29***S.wikstromi* Nupponen, sp. nov., genus combination incertae sedis, male, holotype.

***Paratypes*.** Argentina • 3 ♂, 2 ♀; same data as for holotype; [BOLD sample ID] KN01035; [genitalia slide] K. Nupponen prep. no. 3/14 Dec. 2019 ♀; coll. NUPP.

####### Diagnosis.

Large species (wingspan 20.5–22 mm), greyish brown species, forewing with 3–5 black spots apically near cilia. The weakly resembling “batman” appearance of the male genitalia is distinctive, as well as sternum VIII with a triangular process at middle, attached to transverse plate and enormous round, anterolateral projections. In the female genitalia, a large subtriangular sterigma is characteristic.

####### Description.

Wingspan 20.5–22 mm. Head, collar, neck tuft, haustellum, tegula and thorax unicoloured greyish brown. Scape dorsally beige, ventrally cream; pecten cream and longer than diameter of scape. Flagellum dark brown, 0.65 × length of forewing, in male ciliate, sensillae ~ 1/2 as long as diameter of flagellum. Labial palp: palpomere I white, palpomeres II and III fuscous with a few whitish scales. Legs: foreleg femur dirty white, tibia and tarsus dark brown; midleg and hindleg dirty white except tarsus pale fuscous. Abdomen pale brown, ventrally mixed with white. Forewing greyish brown with sparsely scattered blackish scales; middle part of wing widely but irregularly whitish cream, more whitish at apical area; at 0.7 and 0.85 blackish blotches at middle of wing; apically 3–5 black spots at row near cilia line. Hindwing pale fuscous, fringe slightly darker.

***Male genitalia*.** Uncus quadrangular plate with deep U-shaped medioposterior indentation; sublaterally with small setose flaps. Gnathos not detected. Phallus robust, longer than valva, distal portion tapered. Anterior part of valva wide with round margin, posterior part pointing upwards, incurved, with acute apex. Sternum VIII with large round anterolateral projections, anterior margin widely concave; posterior margin folded forming large transverse bent plate with projected posterolateral corners, and heavily sclerotised triangular process in middle of plate. Tergum VIII trapezoid, posterior portion quadrangular, anterolateral corners broad with small marginal fold.

***Female genitalia*.** Sterigma large subtriangular plate, posterior portion hood-like and heavily sclerotised, tip blunt. Ostium situated in squared sclerotisation at medioposterior margin of sterigma. Sternum VII rectangular. Apophyses anteriores 0.3 × length of apophyses posteriores.

####### Etymology.

Latinised adjective in the nominative singular. The species is named after the type locality, the River San Francisco.

####### Distribution.

NW Argentina.

####### Habitat.

The collecting site is a dry river bed surrounded by forests and plantations. Plants of the family Amaranthaceae were common at riverside (Fig. 79).

####### Genetic data.

BIN: BOLD:ADZ5418 (*n* = 2 from Argentina). Nearest neighbour: *Scythristibicina* Meyrick, 1916 (BIN: BOLD:ADZ4797, 6.68%).

####### Remarks.

Based on COI maximum likelihood phylogeny, taxa *tibicina* and *sanfranciscoensis* group together, associating with other Central and South American taxa, classified in apparently non-monophyletic *Scythris* on BOLD (Suppl. material 2). We classify *tibicina* and *sanfranciscoensis* in *Scythris*.

###### ﻿ Scythris tigrensis

Taxon classificationAnimaliaLepidopteraScythrididae

Nupponen sp. nov.

7F221ADE-7C57-56CF-9C90-55F4909AB705

http://zoobank.org/A186B8F5-710B-4CD6-8838-5C1C27C6E9FB

Figs 26 , 55

####### Type material.

***Holotype*.** Argentina • ♂; prov. Mendoza, Andes Mts., Cordillera del Tigre, Mendoza River valley near Uspallata village; 32°35.9'S, 69°22.9'W; 1900 m a.s.l.; 25 Jan. 2017; K. Nupponen & R. Haverinen leg.; [BOLD sample ID] KN01042; [genitalia slide] K. Nupponen prep. no. 1/8 Dec. 2019; coll. NUPP (MZH).

***Paratype*.** Argentina • 1 ♂; same data as for holotype; coll. NUPP.

####### Diagnosis.

Wings elongated without any distinct pattern, and genitalia examination is indispensable for reliable determination. In the male genitalia of *S.tigrensis*, a narrow distal arm of the gnathos, broad valvae and a conspicuous bifurcate formation attached anteriorly to tegumen are distinctive.

####### Description.

Wingspan 14 mm. Head, haustellum, tegula and thorax beige mixed with cream. Neck tuft white, collar pale beige. Scape dark brown, ventrally with few paler scales; pecten brown and longer than diameter of scape. Flagellum dark brown, 0.65 × length of forewing. Labial palp: palpomere I and base of palpomere II white, otherwise brown more or less mixed with white. Legs: femur and tibia pale beige mixed with fuscous, tarsi fuscous. Abdomen grey, dorsally each segment paler grey scales at posterior margin. Forewing pale beige; indistinct blackish spot in fold at 0.4, and small fuscous spot at cell end; greyish white scales densely scattered in apical area. Hindwing pale fuscous.

***Male genitalia*.** Uncus narrow, digitate, slightly bent downwards. Gnathos base rectangular hood; distal arm narrow, downcurved. Phallus as long as width of valva, bent at middle. Valvae broad and straight, slightly asymmetrical: left one basally with round flap and distally more tapered. Anteriorly to tegumen large bifurcate structure of uncertain homology is attached; left furca (when viewed ventrally) funnel-shaped, longer than valva; right furca 1/2 × shorter, cylindrical, tip pointed, apex with very long and thick seta. Sternum VIII rectangular, 1.7 × higher than wide; posteriorly sclerotised with two narrow and curved projections. Tergum VIII asymmetrical, semi-trapezoid plate.

####### Etymology.

Latinised adjective in the nominative singular. The species is named after the type locality, the mountain range of the Tigre in the Andes.

####### Distribution.

NW Argentina.

####### Habitat.

The collecting site is a dry and xerothermic valley of the River Mendoza at medium altitude of the Andes, surrounded by rocky slopes with sparse and low vegetation.

####### Genetic data.

BIN: BOLD:ADZ5721 (*n* = 1 from Argentina). Nearest neighbour: North American *Neoscythris* sp. (BIN: BOLD:ABA1135, 6.57%).

####### Remarks.

Female unknown. Based on the COI maximum likelihood phylogeny, taxon *tigrensis* belongs to an isolated lineage, being sister to a large lineage containing taxa classified in *Scythris* or in Scythrididae on BOLD (Suppl. material 2). Its morphology does not resemble any other species covered in the study, and even though the barcode gap analysis suggests *Neoscythris* as the nearest neighbour, it does not have the diagnostic characters of that genus (Landry 1991). For practical reasons, we classify *tigrensis* in *Scythris*, but more research is needed.

In our COI maximum likelihood analysis, there are five species, which are structurally heterogenous from each other, and which are distributed in different lineages in the middle-part of the tree (Suppl. material 2, marked with red vertical bar). These are difficult to combine with any North American Scythrididae genus as diagnosed in Landry (1991). We classify these five species, and morphologically similar species without DNA barcodes, to *Scythris* (incertae sedis), highlighting the need for further research. Potentially these taxa should be classified in several genera. These taxa are *S.bicoloristrigella* species group (*bicoloristrigella*, *saldaitisi*, *wikstromi*), *S.andensis* species group (*andensis*, *mendozaensis*) and *S.dividua* species group (*dividua*, *medullata*, *notorrhoa*).

##### The *bicoloristrigella* species group

Distal arm of gnathos upcurved, robust and heavily sclerotised. Valvae asymmetrical. Male sternum VIII elongated, lateromedially with pair of obliquely backwards directed extensions. Species included: *bicoloristrigella*, *saldaitisi*, *wikstromi*.

###### ﻿ Scythris bicoloristrigella

Taxon classificationAnimaliaLepidopteraScythrididae

Nupponen, sp. nov., genus combination incertae sedis

66DEBDA8-3E6F-59E1-82F7-16E0FB64973A

http://zoobank.org/DE27AA3E-8F19-4576-AFF5-AE6C56175C14

Figs 27 , 56

####### Type material.

***Holotype*.** Argentina • ♂; prov. Mendoza, Andes Mts., Cordillera del Tigre, Mendoza River valley near Uspallata village; 32°35.9'S, 69°22.9'W; 1900 m a.s.l.; 25 Jan. 2017; K. Nupponen & R. Haverinen leg.; [BOLD sample ID] KN01056; [genitalia slide] K. Nupponen prep. no. 1/9 Dec. 2019; coll. NUPP (MZH).

***Paratype*.** Argentina • 1 ♂; prov. San Juan, Andes Mts., salt lake by Cordillera del Tigre; 30°52.8'S, 68°52.4'W; 1620 m a.s.l.; 26 Jan. 2017; K. Nupponen & R. Haverinen leg.; coll. NUPP.

####### Diagnosis.

Externally similar to *L.ankylosauroides*, sharing broad white streak in fold of forewing (dorsally and basally white, costally and terminally cream). Examination of the male genitalia is required to safely identify between *S.bicoloristrigella*, *S.saldaitisi* and *S.wikstromi*. In the male genitalia of *S.saldaitisi*, shape of asymmetrical valvae (left one very short) and anterior margin of sternum VIII straight are distinctive (valvae ca. equal length with incurved apexes and anterior margin of sternum VIII concave in *S.wikstromi*, valvae ca. equal length, right valva setose and anterior margin of sternum VIII concave in *S.bicoloristrigella*).

####### Description.

Wingspan 15–16.5 mm. Head, collar, haustellum, tegula and thorax pale beige with scattered cream scales; posterior 1/2 of thorax with longitudinal cream line. Neck tuft white. Scape dorsally fuscous, ventrally pale beige, pecten longer than diameter of scape. Flagellum dark brown, 0.75 × length of forewing, ciliate, sensillae ~ 1.1 × as long as diameter of flagellum. Labial palps: palpomere I white; lower surface of posterior 1/2 of palpomere II and palpomere III dark brown, otherwise greyish white. Legs beige, tibiae darker. Abdomen dorsally beige, ventrally dirty white. Forewing pale fuscous; in fold broad streak from base to cell end: dorsally white from base to 0.6, edged by dark brown line dorsally, costally, and terminally cream; dark brown spots in midwing at 0.5, 0.65 and 0.7; in apical area few dark brown scales. Hindwing pale fuscous.

***Male genitalia*.** Uncus heavily sclerotised hood distally, medioposteriorly indented. Gnathos massive, upturned 90° at basal 1/3, distal portion robust and heavily sclerotised, distally tapered, tip pointed. Tegumen elongated hood, dorsally widely open. Phallus ca. as long as uncus, rather broad, beyond middle bent and chute-shaped. Valvae asymmetrical, left valva 1.3 × as long as right; left valva with semi-circular indentation ventrally at 0.3, distal 0.7 tapered, setose, apically bent, tip pointed; right valva with large triangular lobe ventrally at base, distal 1/2 with numerous thin spiniform setae, dorsally folded, subapically tapered, apex with few minute spines and dense setae. Sternum VIII rectangular, elongated, 3 × longer than wide; posteromedially with very deep U-shaped depression, posterior shanks long, setose; lateromedially margin sclerotised, forming tapered extensions; anterior margin with two short, parallel apodemes. Tergum VIII hexagonal, anterior margin widely concave.

####### Etymology.

Diminuitive noun in apposition. The species name alludes to the bicolored streak in the fold of the forewing.

####### Distribution.

NW Argentina.

####### Habitat.

The habitat is a dry and xerothermic valley of the River Mendoza at medium altitude of the Andes, surrounded by rocky slopes with sparse and low vegetation. The paratype was collected at a xerothermic locality in the middle of a dry lake with sparse halophytic shrubs (Fig. 79).

####### Genetic data.

BIN: BOLD:ADY8267 (*n* = 1 from Argentina). Nearest species: *Scythrissaldaitisi* Nupponen, sp. nov. (BIN: BOLD:ADZ5132, 5.3%).

####### Remarks.

Female unknown. Based on COI maximum likelihood phylogeny and morphology, South American taxa *bicoloristrigella*, *saldaitisi*, and *wikstromi* group together, associating next to the North American taxa classified in *Scythris*, *Rhamphura*, or *Neoscythris* on BOLD (Suppl. material 2). We are unable to classify these with certainty to any Scythrididae genus as diagnosed and illustrated in Landry (1991) and Bengtsson (2014). We therefore took a conservative view and classified these taxa in *Scythris* (incertae sedis), highlighting the need for further research.

###### ﻿ Scythris saldaitisi

Taxon classificationAnimaliaLepidopteraScythrididae

Nupponen, sp. nov., genus combination incertae sedis

44EA2D5D-36F3-59DD-B290-4A1063237F1E

http://zoobank.org/AC91FD2A-C150-4280-B406-CF2057BB5E97

Figs 28 , 57

####### Type material.

***Holotype*.** Argentina • ♂; prov. Catamarca, Sierra de Manchao; 28°47.9'S, 66°23.2'W; 970 m a.s.l.; 21 Sep. 2017; K. Nupponen & R. Haverinen leg.; [BOLD sample ID] KN01049; [genitalia slide] K. Nupponen prep. no. 4/11 Dec. 2019; coll. NUPP (MZH).

####### Diagnosis.

Identification requires examination of the genitalia. In the male genitalia of *S.saldaitisi*, shape of asymmetrical valvae (left one very short) and anterior margin of sternum VIII straight are distinctive (valvae ca. equal length with incurved apexes and anterior margin of sternum VIII concave in *S.wikstromi*, valvae ca. equal length, right valva setose and anterior margin of sternum VIII concave in *S.bicoloristrigella*).

####### Description.

Wingspan 13 mm. Head, collar, neck tuft, haustellum, tegula and thorax dark brown. Few dirty white scales exist around eye and laterally at thorax. Scape dorsally dark brown, ventrally pale beige, pecten longer than diameter of scape. Flagellum dark brown, 0.7 × length of forewing, ciliate, sensillae ~ 1/2 as long as diameter of flagellum. Labial palp: palpomere I dirty white, palpomeres II and III dark brown mixed with a few dirty white scales. Legs: lower surfaces dirty white, otherwise foreleg and midleg dark brown and hindleg fuscous. Abdomen dorsally fuscous, ventrally dirty white. Forewing with costal and apical areas dark brown, dorsal area at basal 1/2 slightly paler; black blotches in fold at 0.2, 0.35, 0.55, and at cell end, between two basal ones pale beige dash; scattered with white scales, more pronounced at apical area. Hindwing fuscous, slightly paler than forewing.

***Male genitalia*.** Uncus elongated hood, posterior 1/2 heavily sclerotised. Gnathos base broad; distal arm upcurved, robust, heavily sclerotised and of constant width, tip blunt. Tegumen oval hood, dorsally widely open. Phallus 1/2 as long as left valva, rather slim, slightly bent. Valvae asymmetrical; left valva short, almost straight; right valva twice longer and wider, basal 0.7 straight, apical quarter somewhat twisted and curved ventrad, tip heavily sclerotised with hook-shaped process. Saccus labiate, ~ 1/2 length of left valva. Juxta narrow, 1.4 × length of phallus. Sternum VIII rectangular, elongated, 3.5 × longer than wide; posteromedially with two pronged projections with pointed tips; laterobasally with a pair of tapered extensions, directed obliquely anteriorly. Tergum VIII trapezoid, anterior margin concave and sclerotised.

####### Etymology.

Noun in the genitive case. The species is dedicated to Aidas Saldaitis, a Lithuanian lepidopterist, to acknowledge his contributions to Scythrididae systematics.

####### Distribution.

NW Argentina.

####### Habitat.

Habitat is a dry and xerothermic rocky slope with low vegetation and rather densely occuring bushes.

####### Genetic data.

BIN: BOLD:ADZ5132 (*n* = 1 from Argentina). Nearest neighbour: *S.bicoloristrigella* (BIN: BOLD:ADY8267, 5.3%).

####### Remarks.

Female unknown. Based on COI maximum likelihood phylogeny and morphology, South American taxa *bicoloristrigella*, *saldaitisi*, and *wikstromi* group together, associating next to the North American taxa classified in *Scythris*, *Rhamphura*, or *Neoscythris* on BOLD (Suppl. material 2). We are unable to classify these with certainty to any Scythrididae genus as diagnosed and illustrated in Landry (1991) and Bengtsson (2014). We therefore took a conservative view and classified these taxa in *Scythris* (incertae sedis), highlighting the need for fur­ther research.

###### ﻿ Scythris wikstromi

Taxon classificationAnimaliaLepidopteraScythrididae

Nupponen, sp. nov., genus combination incertae sedis

93E1D08D-FC82-5EA2-9412-8FA86A00CE51

http://zoobank.org/A3D5F3E0-DC48-4A99-B453-A20C1D2A51CB

Figs 29 , 58

####### Type material.

***Holotype*.** Argentina • ♂; prov. Cordoba, Salinas Grandes SE shore; 29°50.5'S, 64°40.2'W; 185 m a.s.l.; 24 Sep. 2017; K. Nupponen & R. Haverinen leg.; [BOLD sample ID] KN01067; [genitalia slide] K. Nupponen prep. no. 2/12 Dec. 2019; coll. NUPP (MZH).

####### Diagnosis.

Safely determined only by dissecting the genitalia. In the male genitalia of *S.wikstromi*, the valvae are asymmetrical and sickle-shaped (right arm short and setose in *S.bicoloristrigella*, left arm short in *S.saldaitisi*), and sternum VIII posteriorly with wide indentation and wide projections (narrower indentation and long projections in *S.bicoloristrigella* and *S.saldaitisi*).

####### Description.

Wingspan 11.5 mm. Head, collar, neck tuft, haustellum, tegula and thorax brown with scattered cream scales. Scape dorsally brown, ventrally somewhat paler, pecten longer than diameter of scape. Flagellum dark brown, 0.7 × length of forewing, ciliate, sensillae ~ 0.65 × as long as diameter of flagellum. Labial palp: palpomere I pale cream, palpomeres II and III beige of various tones. Legs: foreleg dark brown, midleg and hindleg pale brown; lower surface pale beige. Abdomen dorsally fuscous, ventrally whitish grey. Forewing brown; black blotches in dorsum at 0.1, midwing at 0.35, and small ones above tornus and at cell end; white blotch in midwing at 0.25. Hindwing fuscous.

***Male genitalia*.** Uncus elongate hood, posterior 1/2 heavily sclerotised with small semi-circular flap at middle. Gnathos base broad bel, attached to tegumen by membrane; distal arm upcurved, robust, heavily sclerotised and of constant width, tip blunt. Tegumen oval hood, dorsally widely open. Phallus ca. as long as uncus, rather slim, medially little constricted. Valvae asymmetrical, long and slender, basally fused by hood-like formation; basal 1/2 of both valvae rather broad, dorsally with small triangular lobes at 0.4; distal 1/2 tapered and incurved (sickle-shaped), tips heavily sclerotised and pointed, right valva 1.2 × longer. Juxta narrow, 1.1 × length of phallus. Sternum VIII hexagonal basally, medioposteriorly wide and deep U-shaped indentation; medio-anteriorly with sclerotised semicircle. Tergum VIII trapezoid, anterior margin concave and sclerotised.

####### Etymology.

Noun in the genitive case. The diacritic mark “ö” is deleted, following ICZN (2000) paragraph 32.5.2.1.The species is dedicated to Bo Wikström, a Finnish lepidopterist.

####### Distribution.

NW Argentina.

####### Habitat.

The collecting site is the shore of a large salt lake, in the zone between a dry shrubby area and low halophytic vegetation.

####### Genetic data.

Not obtained (specimen submitted to barcode analysis but the sample failed).

####### Remarks.

Female unknown. Based on COI maximum likelihood phylogeny and morphology, South American taxa *bicoloristrigella*, *saldaitisi*, and *wikstromi* group together, associating next to the North American taxa classified in *Scythris*, *Rhamphura*, or *Neoscythris* on BOLD (Suppl. material 2). We are unable to classify these with certainty to any Scythrididae genus, as diagnosed and illustrated in Landry (1991) and Bengtsson (2014). We therefore took a conservative view and classified these taxa in *Scythris* (incertae sedis), highlighting the need for further research.

##### The *andensis* species group

Sterigma rocket-shaped (pentagonal) in the female genitalia. Gnathos of *S.andensis* with large, tooth-like extensions on ventral margin. Male of *S.mendozaensis* is unknown. Species included: *andensis*, *mendozaensis*.

###### ﻿ Scythris andensis

Taxon classificationAnimaliaLepidopteraScythrididae

Nupponen, sp. nov., genus combination incertae sedis

DE63C7BA-6B71-553D-9683-E8E157257E03

http://zoobank.org/7515D97A-17AB-43C9-9603-4DD29DCAD6C5

Figs 30 , 59 , 71

####### Type material.

***Holotype*.** Argentina • ♂; prov. La Rioja, Andes Mts., Sierra de Famatina, Famatina village 15 km NNW; 28°46.4'S, 67°35.0'W; 2085 m a.s.l.; 27 Jan. 2017; K. Nupponen & R. Haverinen leg.; BOLD sample ID KN01064; [genitalia slide] K. Nupponen prep. no. 2/15 Dec. 2019; coll. NUPP (MZH).

***Paratypes*.** Argentina • 14 ♂, 2 ♀; same data as for holotype; [BOLD sample IDs] KN01063, KN01065, KN01066; [genitalia slide] K. Nupponen prep. no. 1/13 Jan. 2019 ♂; coll. NUPP; • 1 ♀; prov. San Juan, Andes Mts., salt lake by Cordillera del Tigre; 30°52.8'S, 68°52.4'W; 1620 m a.s.l.; 26 Jan. 2017; K. Nupponen & R. Haverinen leg.; [genitalia slide] K. Nupponen prep. no. 4/14 Dec. 2019; coll. NUPP.

####### Diagnosis.

Externally resembling *S.saldaitisi* and *S.wikstromi*, sharing with those whitish blotches on forewings, and reliable determination can be achieved by genitalia examination. In the male genitalia of *S.andensis*, gnathos with tooth-like extensions on ventral margin, valvae are slim and asymmetrical, phallus is very short, and sclerites on segment VIII are asymmetrical and elongated. In the female genitalia the sclerotised, rocket-shaped sterigma is characteristic.

####### Description.

Wingspan 12.5–13 mm. Head, collar, tegula and thorax pale fuscous; thorax laterally and collar with few white scales. Neck tuft white. Haustellum base cream. Scape dorsally dark brown, ventrally white with pecten of same colour. Flagellum dark brown, 0.7 × length of forewing, in male ciliate, sensillae ~ 1/2 as long as diameter of flagellum. Labial palp pale brown, palpomere I and upper surface mixed with white. Legs white, upper surfaces more or less mixed with different tones of brown. Abdomen dorsally fuscous, ventrally dirty white in male and white in female. Forewing pale fuscous, more or less densely scattered by white scales; large white blotches at midwing subbasally, at 0.35 between fold and dorsum, and above tornus; large dark brown blotches between fold and dorsum at 0.2 and 0.45, and spot of same colour at cell end. Hindwing pale fuscous.

**Figures 30–34.**
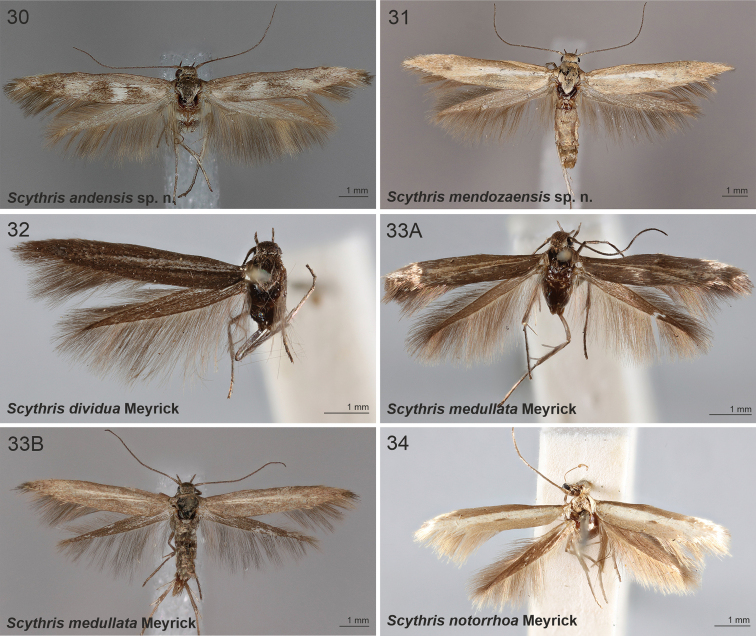
Scythrididae adults, genus *Scythris***30***S.andensis* Nupponen sp. nov., genus combination incertae sedis, male, holotype **31***S.mendozaensis* Nupponen sp. nov., genus combination incertae sedis, female, holotype **32***S.dividua* Meyrick, 1916, genus combination incertae sedis, male, lectotype **33A***S.medullata* Meyrick, genus combination incertae sedis, 1916, male, lectotype **33B***S.medullata* Meyrick, 1916, genus combination incertae sedis, male **34***S.notorrhoa* Meyrick, 1921, genus combination incertae sedis, male, lectotype.

***Male genitalia*.** Uncus as long as gnathos and tegumen together, basally subquadrangular, distally narrow and shallowly upcurved, tip pointed. Gnathos long and robust, tip bifurcate, at base large asymmetrical extension; ventral edges with heavily sclerotised tooth-like extensions, four on right side and five on left side; dorsal surface subapically long and slender with weakly sclerotised extension, with two small basal thorns (potentially anal tube). Tegumen hood-shaped. Phallus thick, straight and very short. Valvae long and narrow, asymmetrical; left valva tapered distally, right distally spatulate. Saccus short, labiate. Sternum VIII large, elongated, triangular basally but asymmetrical, posteriorly digitate. Tergum VIII narrower and little longer than sternum VIII, otherwise similar. Segment VIII is somewhat twisted *in situ*.

***Female genitalia*.** Sterigma rocket-shaped, thick and robust. Ostium small, situated at tip of sterigma. Sternum VII rectangular, 1.4 × wider than high. Apophyses anteriores 1/2 length of apophyses posteriores.

**Figures 35–36.**
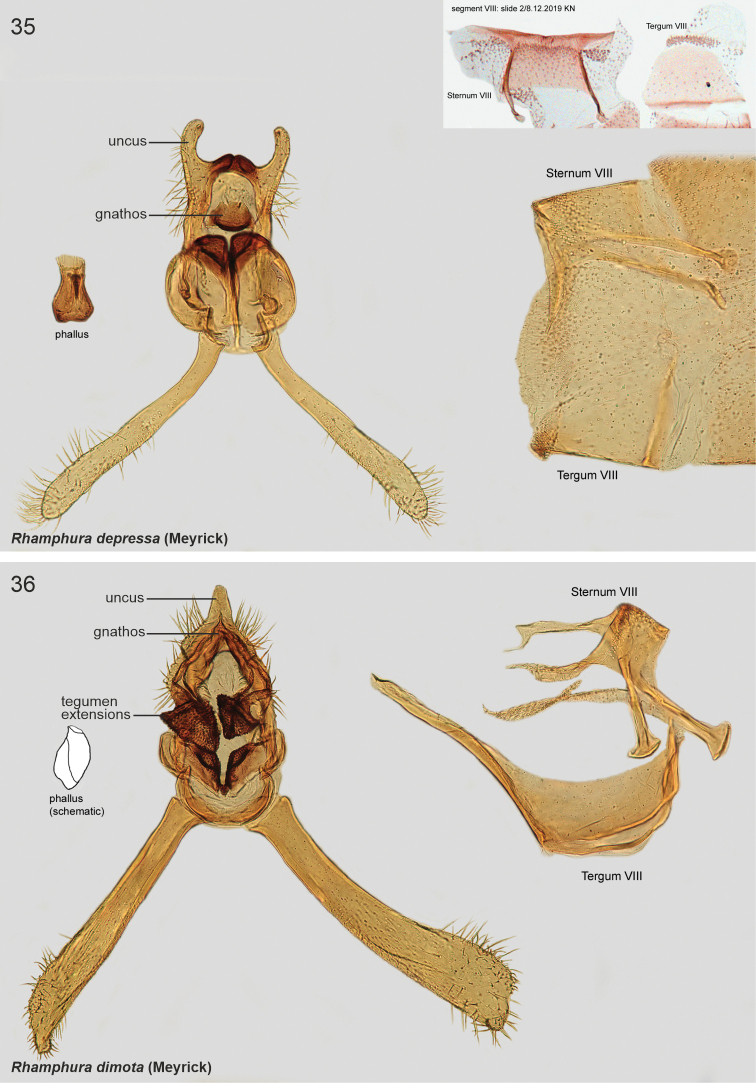
Male genitalia of *Rhamphura***35***R.depressa* (Meyrick, 1931), holotype, slide JFGC No. 8061 **36***R.dimota* (Meyrick, 1931), lectotype, slide JFGC No. 8062.

**Figures 37–38.**
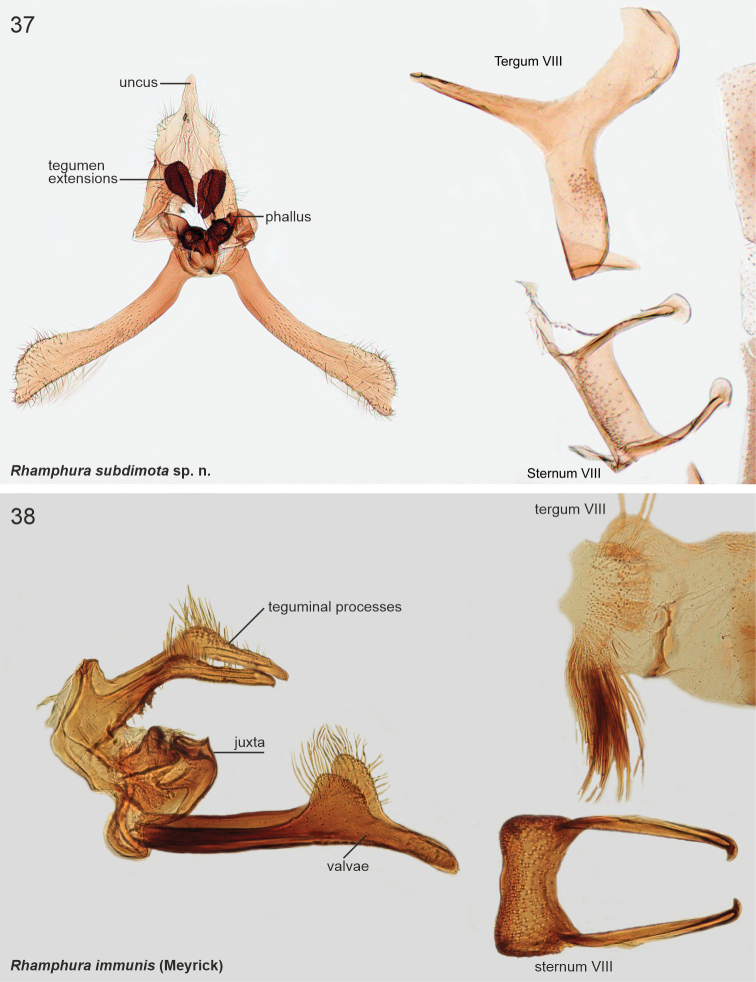
Male genitalia of *Rhamphura***37***R.subdimota* Nupponen sp. nov., holotype, slide 5/12 Dec. 2019 KN **38***R.immunis* (Meyrick, 1916), lectotype, slide JFGC No. 8056, genitalia shown in lateral view.

**Figures 39–40.**
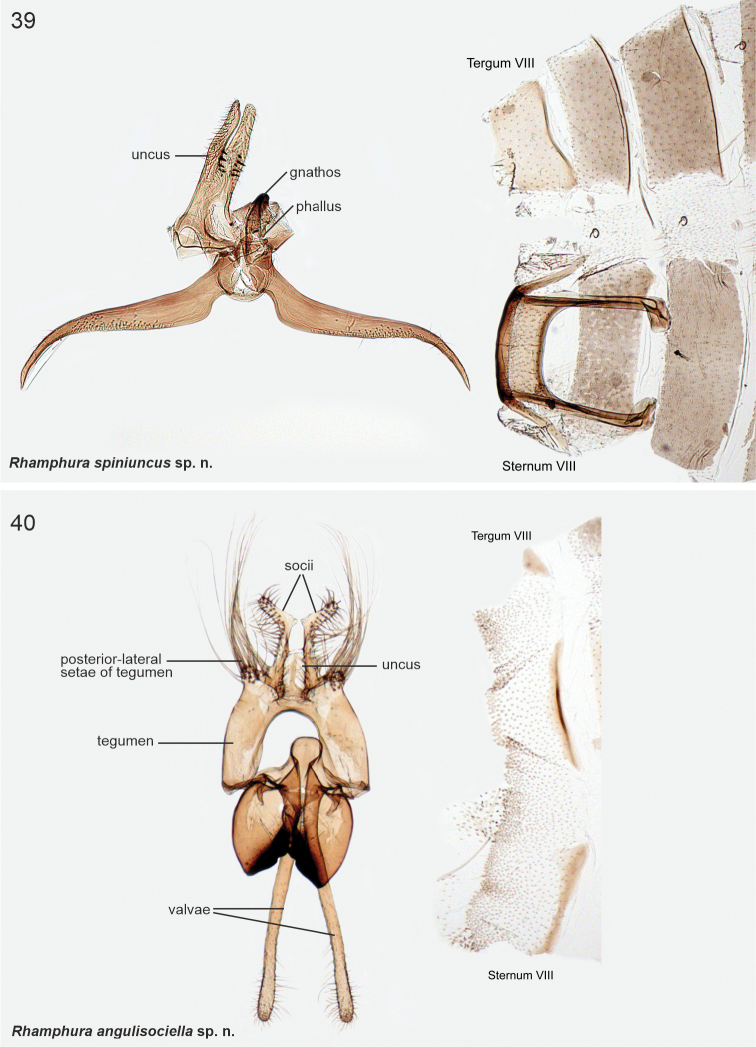
Male genitalia of *Rhamphura***39***R.spiniuncus* Nupponen sp. nov., holotype, slide 4/12 Dec. 2019 KN) **40***R.angulisociella* Nupponen sp. nov., genus combination incertae sedis, holotype, slide 1/10 Dec. 2019 KN.

**Figures 41–42.**
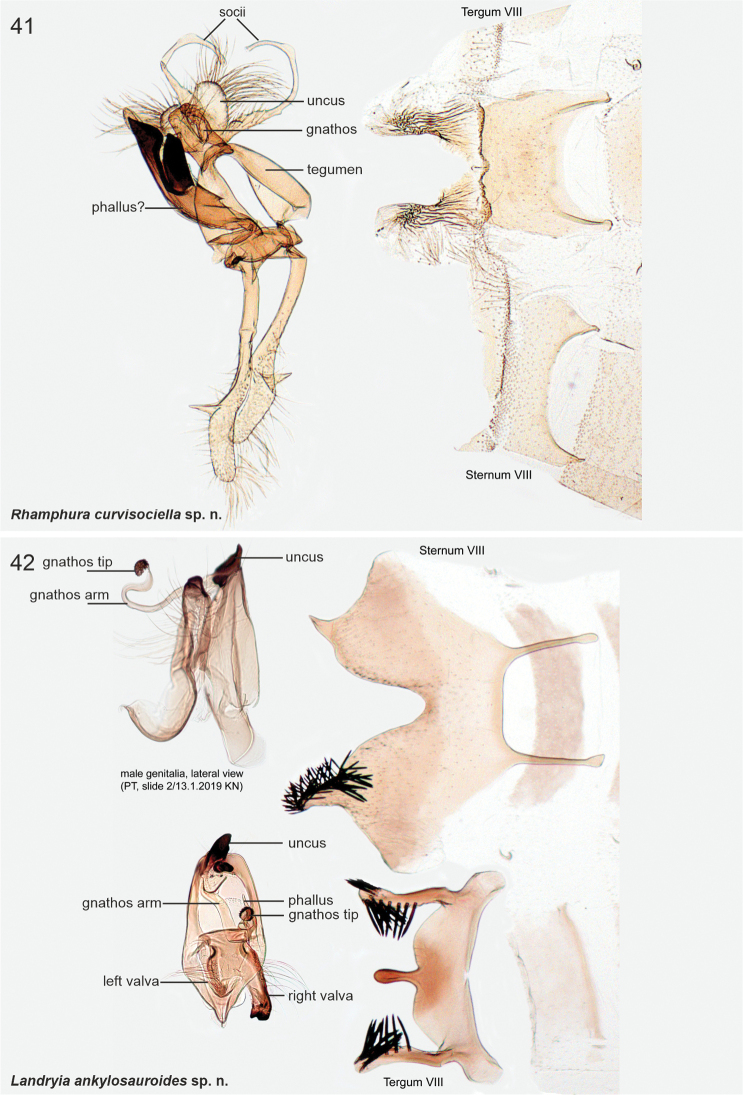
Male genitalia of *Rhamphura* and *Landryia***41***R.curvisociella* Nupponen sp. nov., genus combination incertae sedis, holotype, slide 1/12 Dec. 2019 KN **42***L.ankylosauroides* Nupponen, sp. nov., genus combination incertae sedis, holotype, above (lateral view): slide 4/13 Dec.2019 KN, below (ventral view): slide 2/13 Dec. 2019 KN (paratype).

**Figures 43–44.**
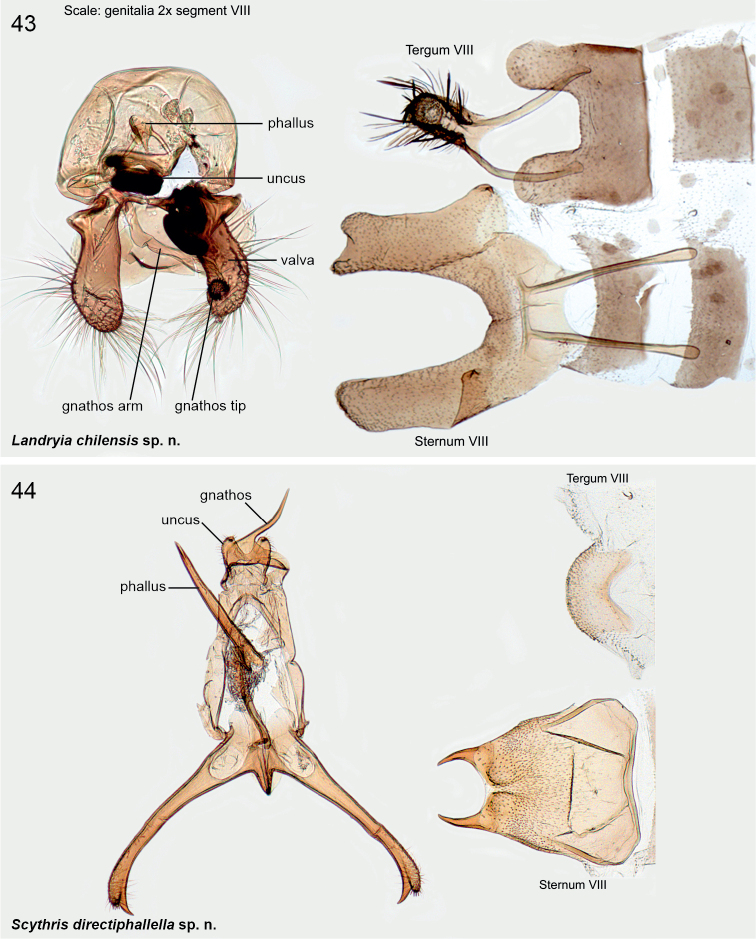
Male genitalia of *Landryia* and *Scythris***43***L.chilensis* Nupponen sp. nov., genus combination incertae sedis, holotype, slide 3/18 Dec. 2019 KN **44***S.directiphallella* Nupponen sp. nov., holotype; slide 3/28 Dec.2019 KN.

**Figures 45–46.**
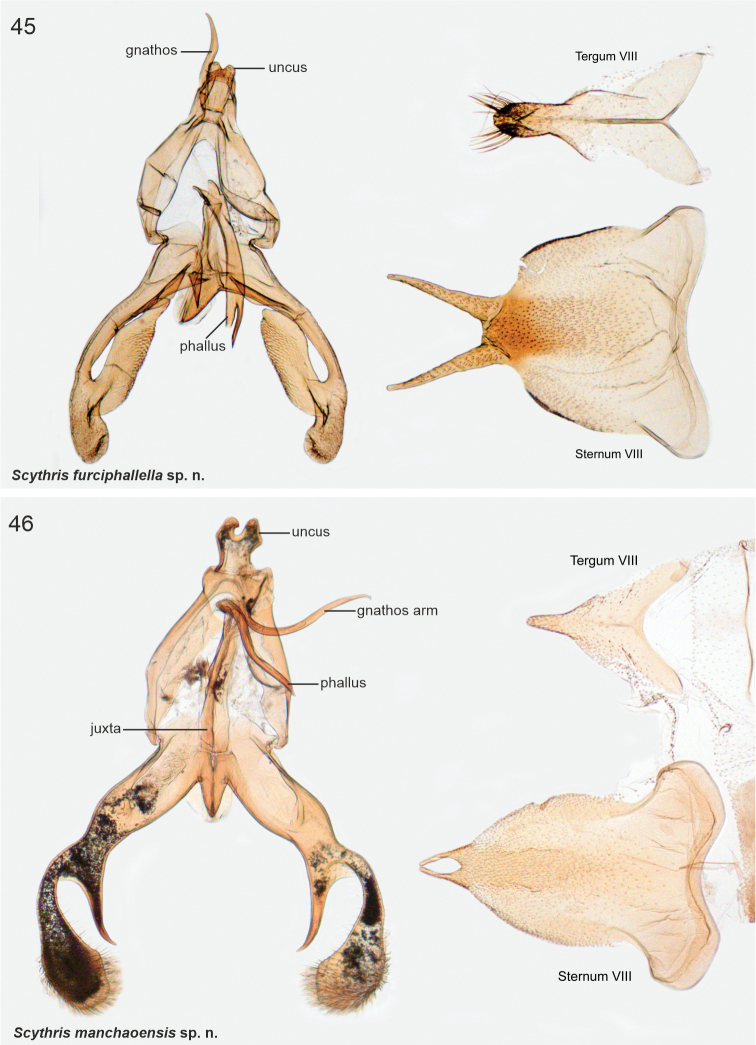
Male genitalia of *Scythris***45***S.furciphallella* Nupponen sp. nov., holotype, slide 2/16 Dec. 2019 KN **46***S.manchaoensis* Nupponen sp. nov., holotype, slide 1/11 Dec. 2019 KN.

**Figures 47–48.**
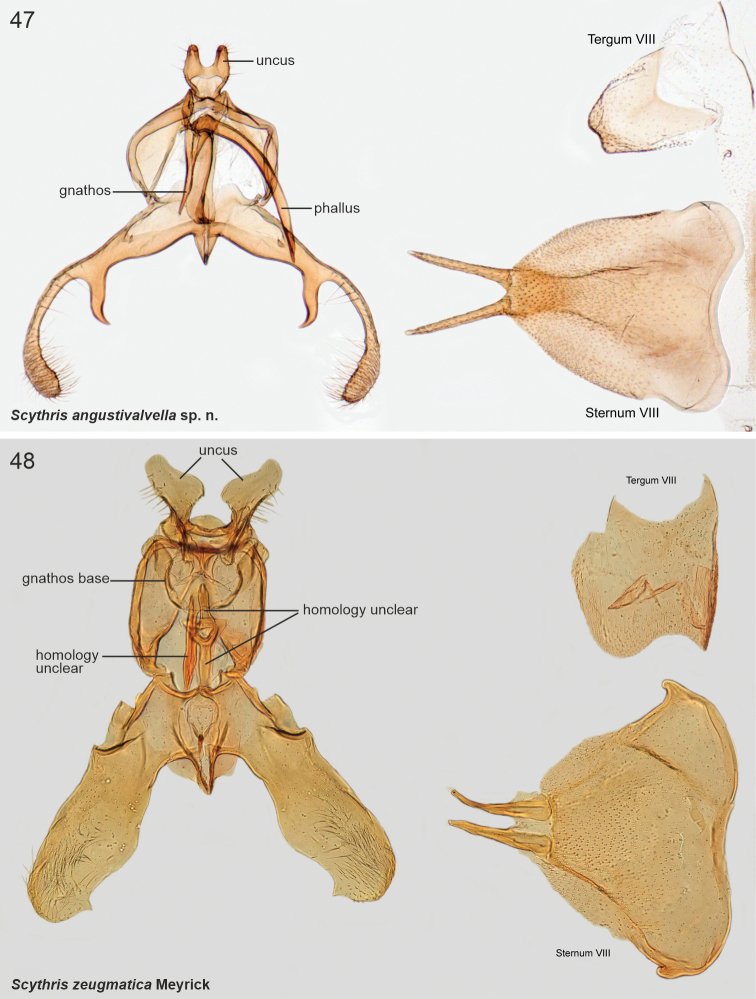
Male genitalia of *Scythris***47***S.angustivalvella* Nupponen sp. nov., holotype, slide 4/16 Dec. 2019 KN **48***S.zeugmatica* Meyrick, 1931, holotype, slide JFGC No. 8050.

**Figures 49–50.**
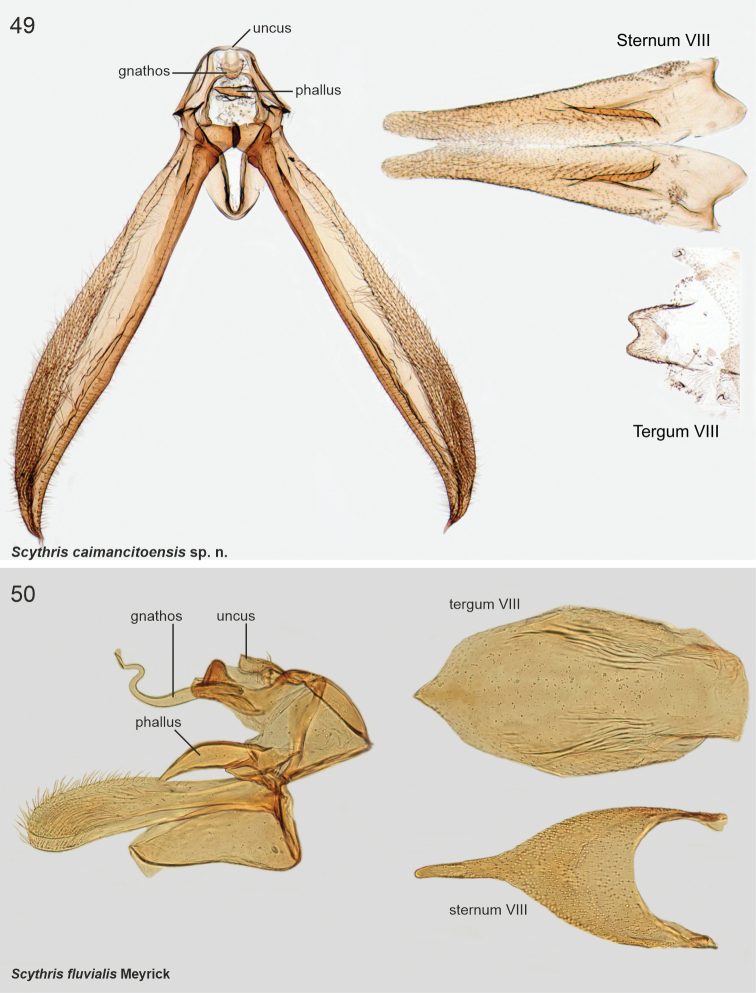
Male genitalia of *Scythris***49***S.caimancitoensis* Nupponen sp. nov., holotype, slide 3/9 Dec. 2019 KN **50***S.fluvialis* Meyrick, 1916, lectotype, slide JFGC No. 8052 (genitalia shown in lateral view).

**Figures 51–52.**
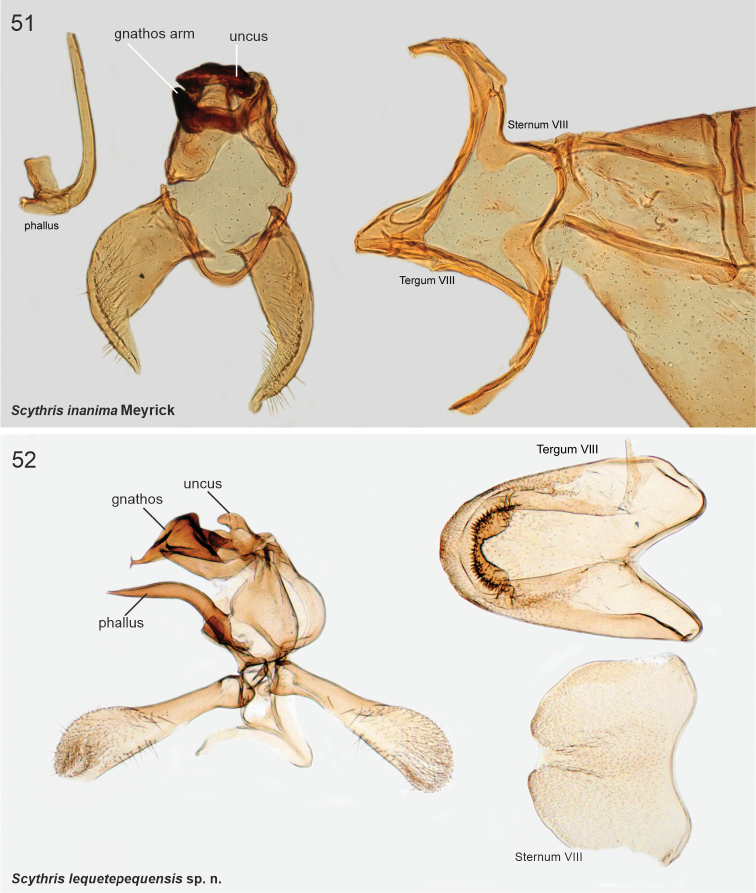
Male genitalia of *Scythris***51***S.inanima* Meyrick, 1916, holotype, slide JFGC No. 8051 **52***S.lequetepequensis* Nupponen sp. nov., holotype, slide 2/8 Dec. 2019 KN.

**Figures 53–54.**
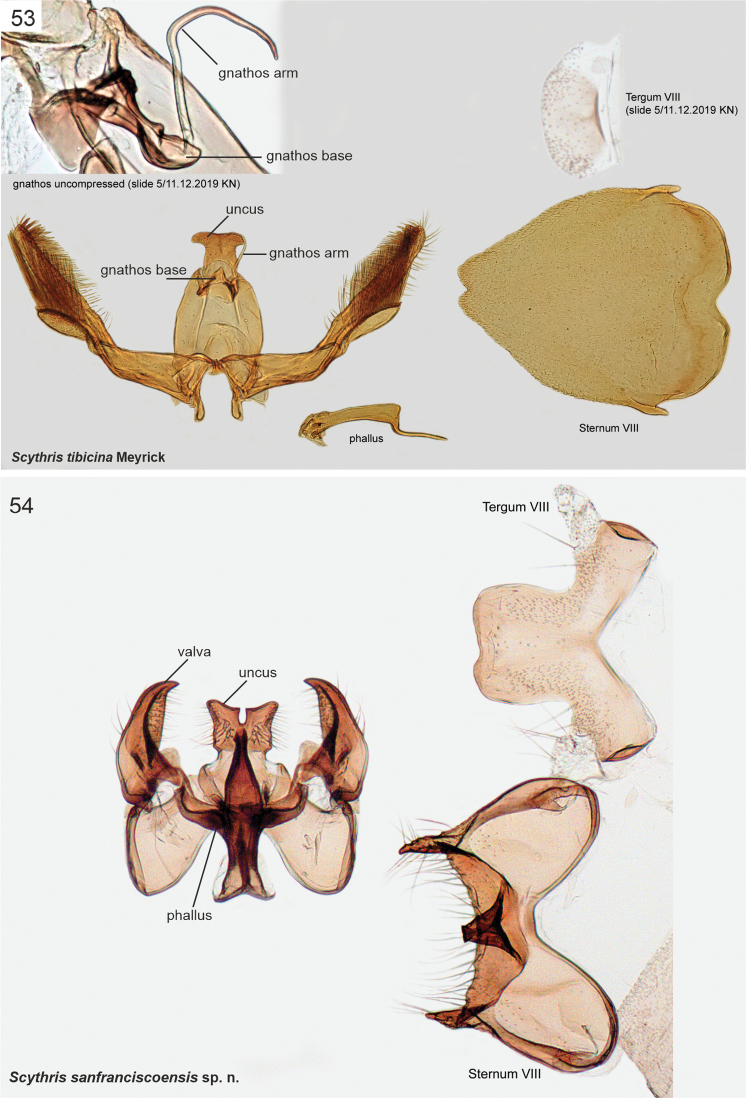
Male genitalia of *Scythris***53***S.tibicina* Meyrick, 1916, lectotype, slide JFGC No. 8053 (top right corner: tergum VIII and gnathos uncompressed, slide 5/11 Dec. 2019 KN **54***S.sanfranciscoensis* Nupponen sp. nov., holotype, slide 2/10 Dec. 2019 KN.

**Figures 55–56.**
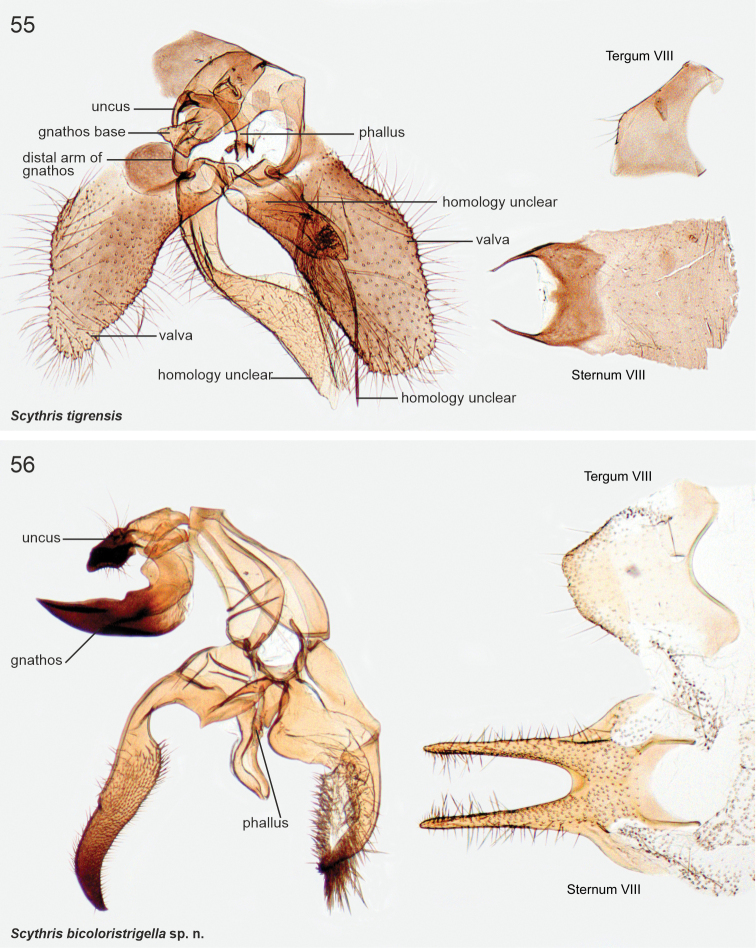
Male genitalia of *Scythris***55***S.tigrensis* Nupponen sp. nov., holotype, slide 1/08 Dec. 2019 KN **56***S.bicoloristrigella* Nupponen sp. nov., genus combination incertae sedis, holotype, slide 1/9 Dec. 2019 KN.

**Figures 57–58.**
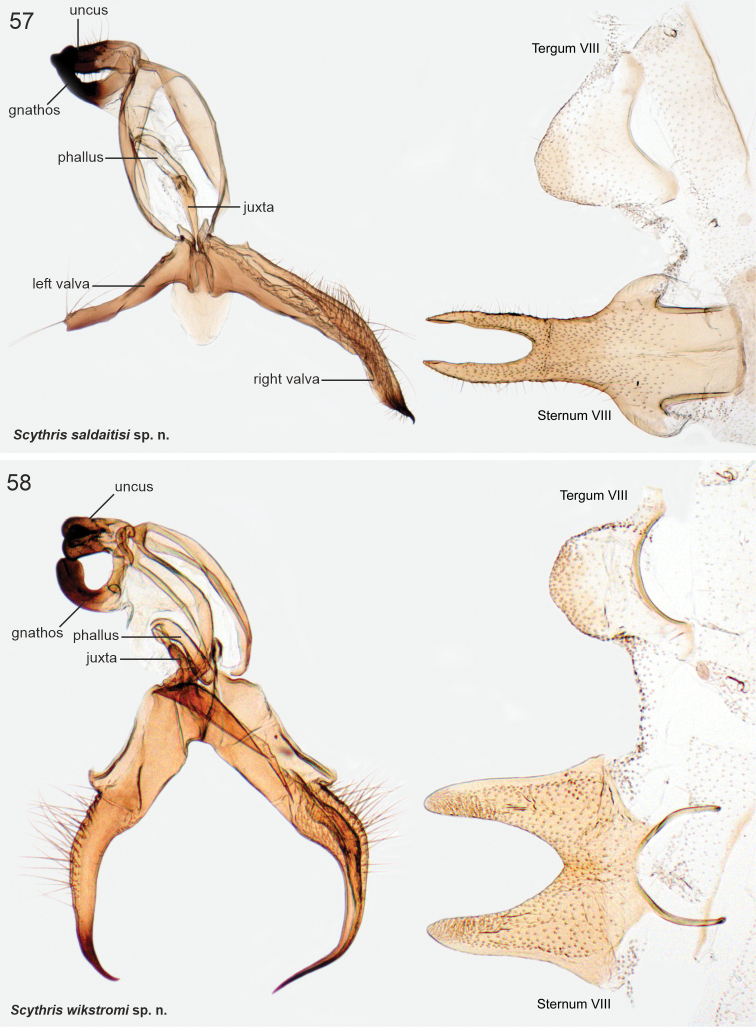
Male genitalia of *Scythris***57***S.saldaitisi* Nupponen sp. nov., genus combination incertae sedis, holotype, slide 4/11 Dec. 2019 KN **58***S.wikstromi* Nupponen, sp. nov., genus combination incertae sedis, holotype, slide 2/12 Dec. 2019 KN.

**Figures 59–60.**
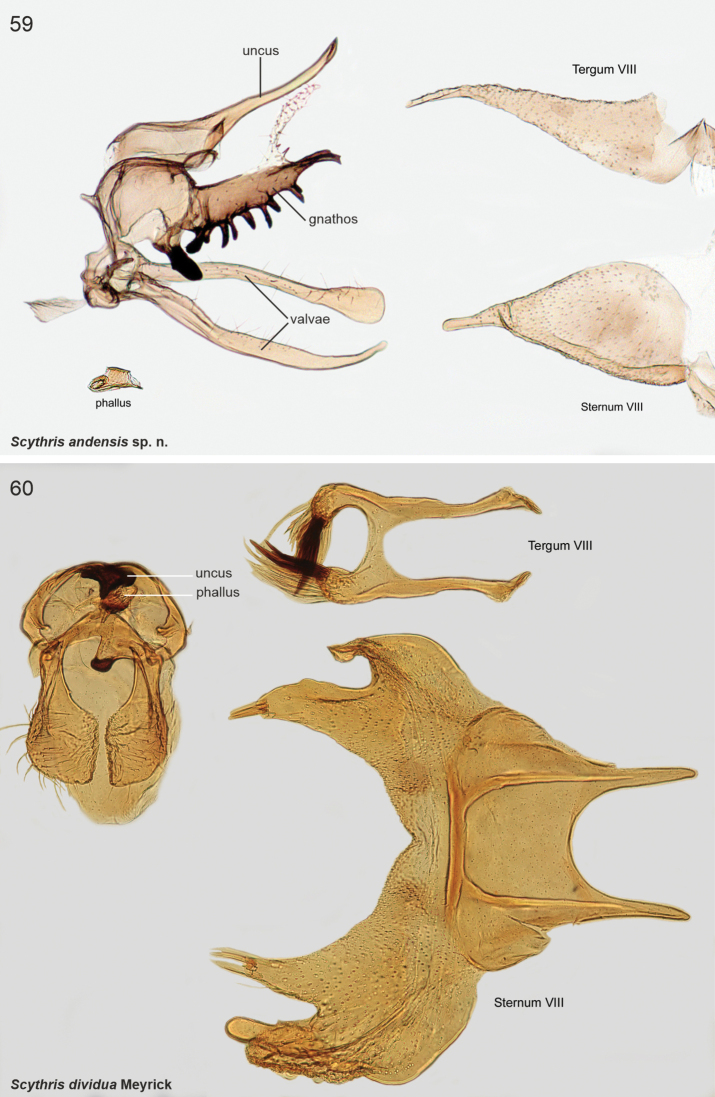
Male genitalia of *Scythris***59***S.andensis* Nupponen sp. nov., genus combination incertae sedis, holotype, slide 2/15 Dec. 2019 KN (genitalia shown in lateral view) **60***S.dividua* Meyrick, 1916, genus combination incertae sedis, lectotype, slide JFGC No. 8054.

**Figures 61–62.**
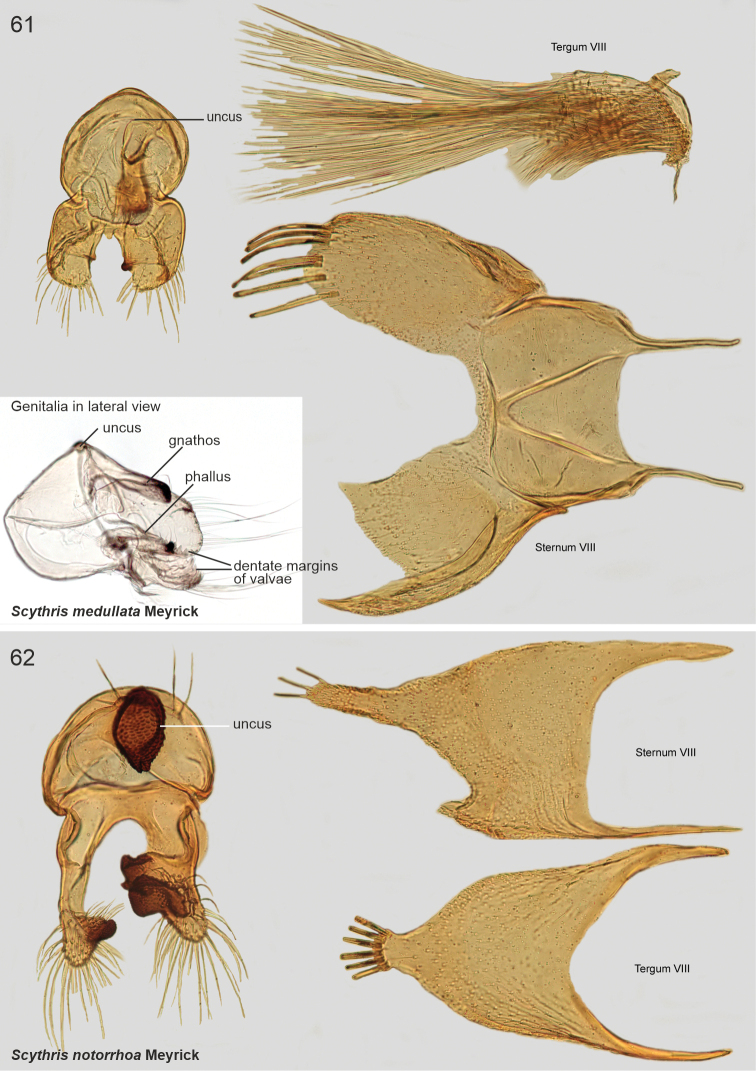
Male genitalia of *Scythris***61***S.medullata* Meyrick, 1916, genus combination incertae sedis, lectotype, slide JFGC No. 8055 **62***S.notorrhoa* Meyrick, 1921, genus combination incertae sedis, lectotype, slide JFGC No. 8065.

**Figure 63.**
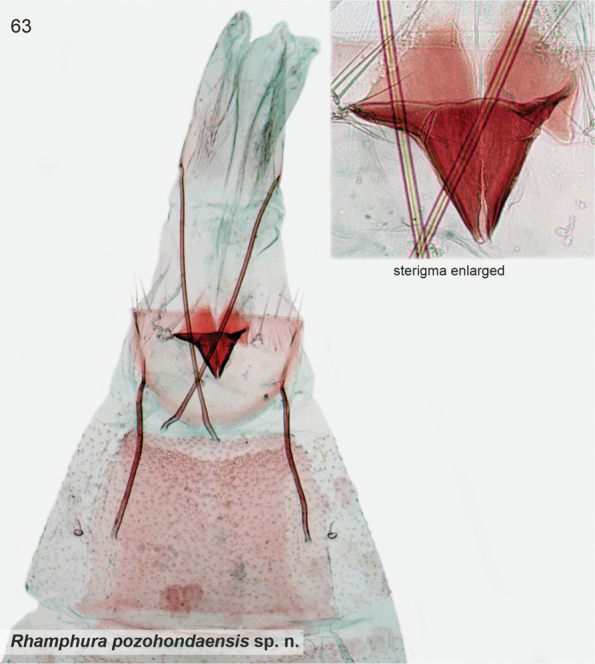
Female genitalia of *Rhamphurapozohondaensis* Nupponen sp. nov., holotype, slide 1/14 Dec. 2019 KN.

**Figure 64.**
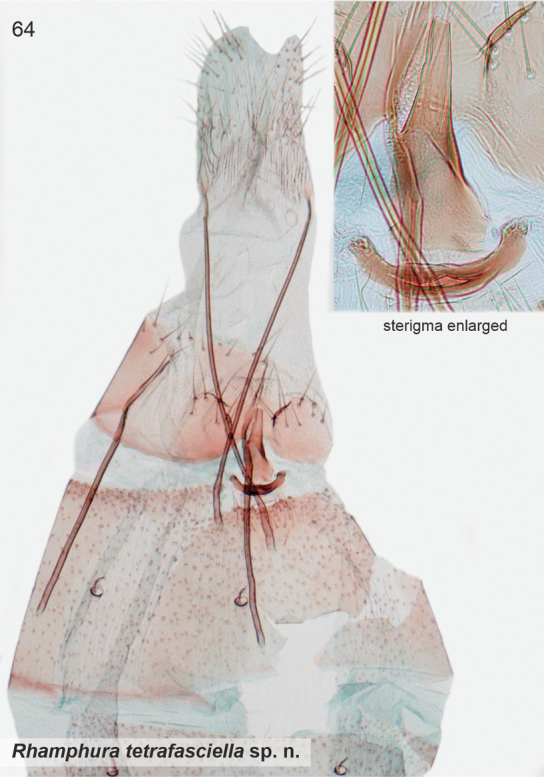
Female genitalia of *Rhamphuratetrafasciella* Nupponen sp. nov., genus combination incertae sedis, holotype, slide 3/13 Dec. 2019 KN.

**Figure 65.**
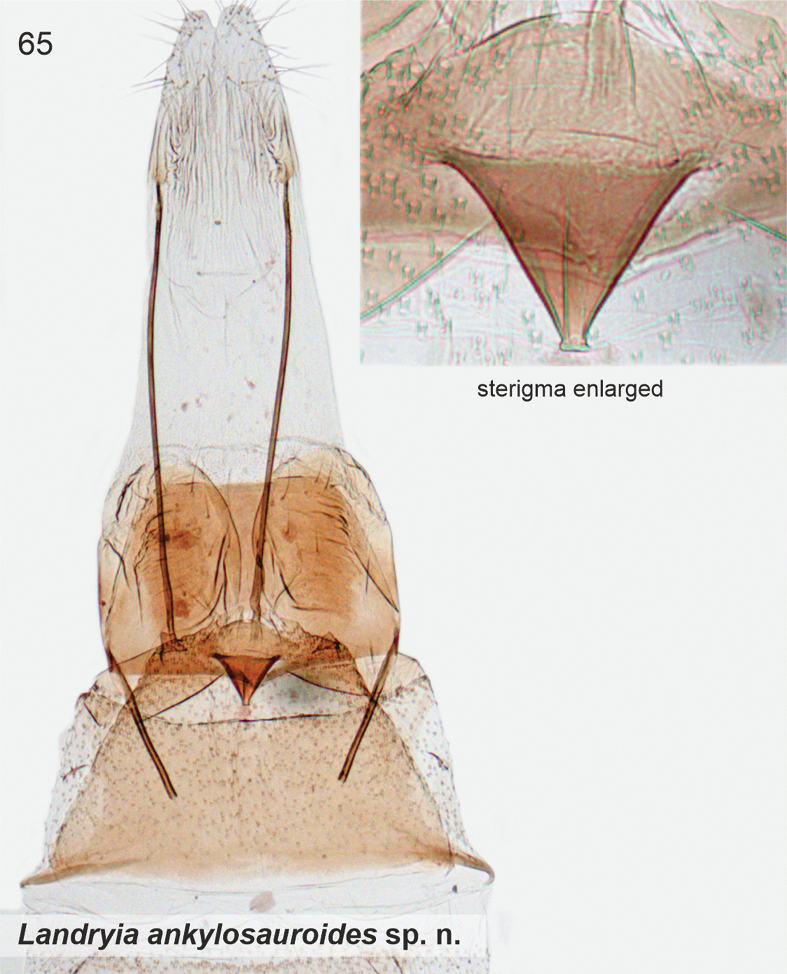
Female genitalia of *Landryiaankylosauroides* Nupponen sp. nov., genus combination incertae sedis, paratype, slide 1/15 Dec.2019 KN.

**Figure 66.**
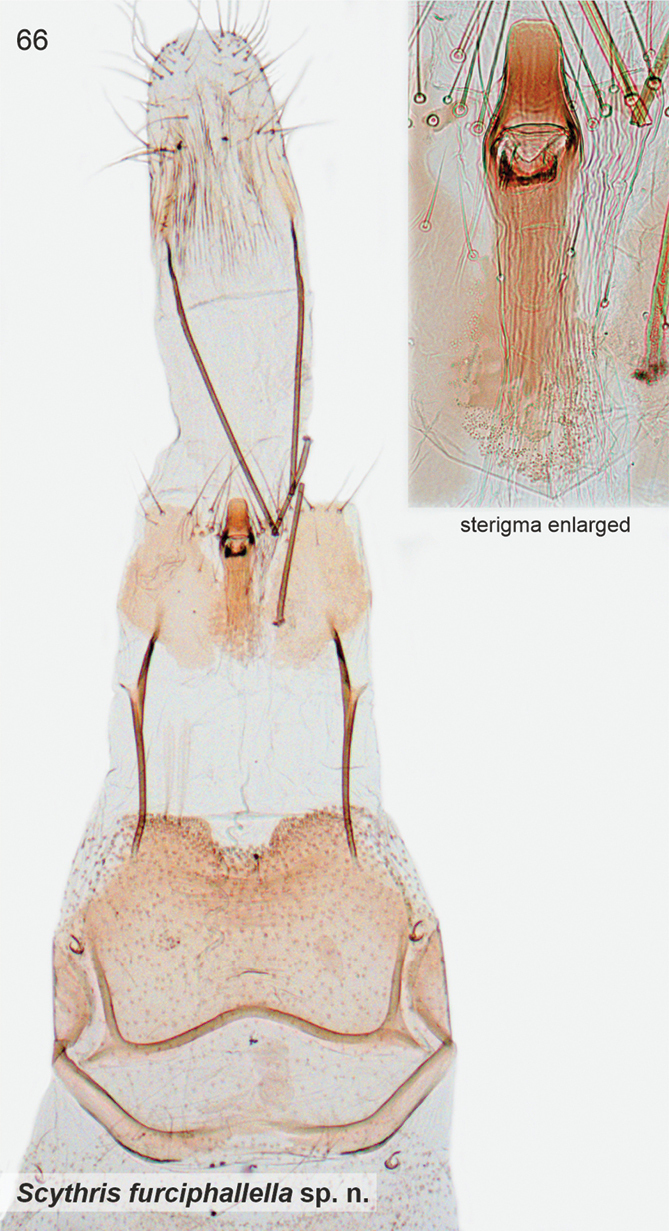
Female genitalia of *Scythrisfurciphallella* Nupponen sp. nov., paratype, slide 3/16 Dec. 2019 KN.

**Figure 67.**
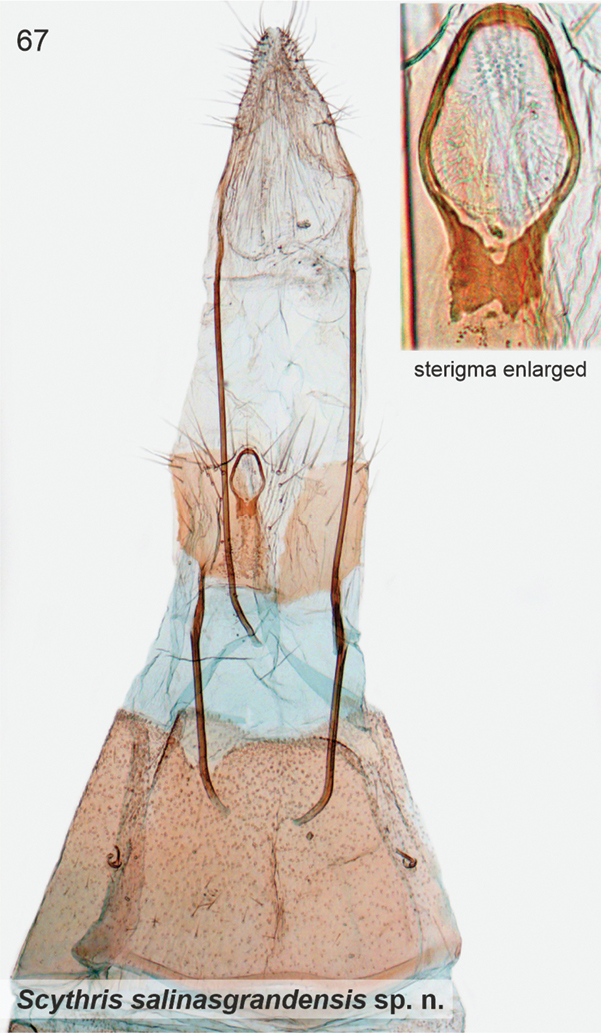
Female genitalia of *Scythrissalinasgrandensis* Nupponen sp. nov., holotype, slide 3/11 Dec. 2019 KN.

**Figure 68.**
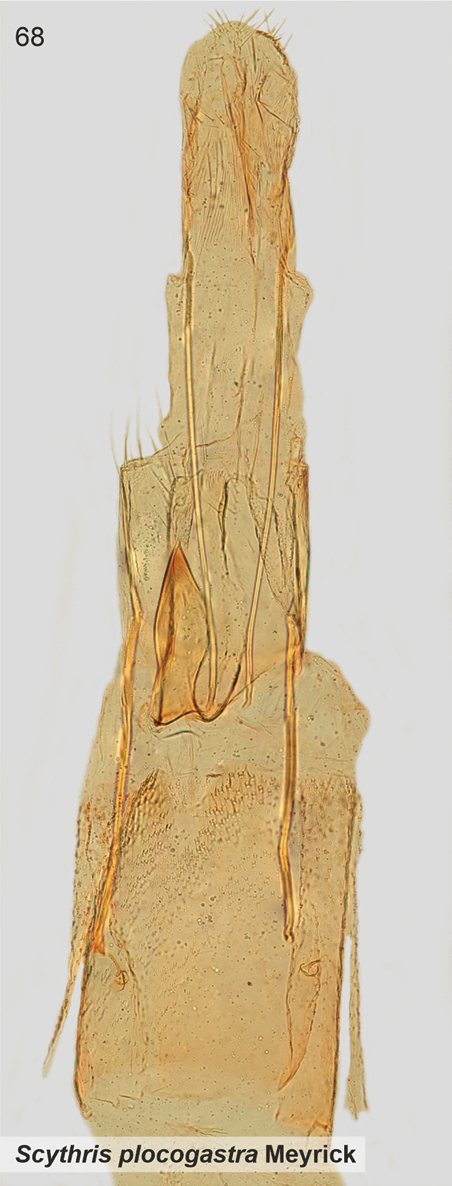
Female genitalia of *Scythrisplocogastra* Meyrick, 1931, holotype, slide J.F.G.C. No. 8063.

**Figure 69.**
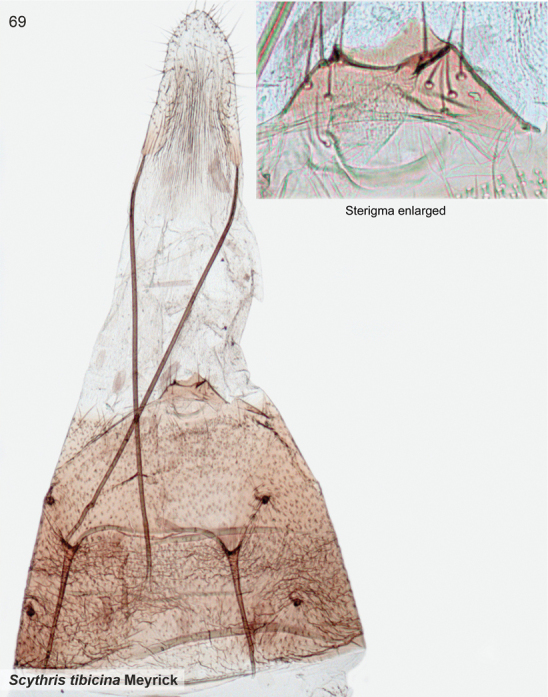
Female genitalia of *Scythristibicina* Meyrick, 1916, slide 1/18 Dec. 2019 KN.

**Figure 70.**
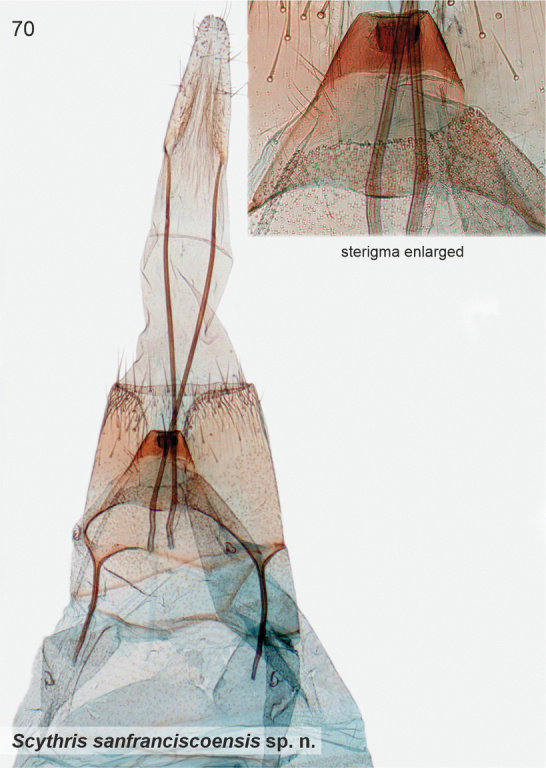
Female genitalia of *Scythrissanfranciscoensis* Nupponen sp. nov., paratype, slide 3/14 Dec. 2019 KN.

**Figure 71.**
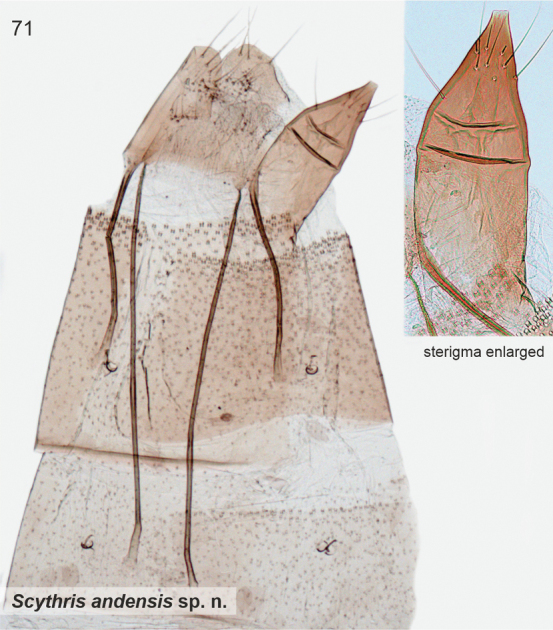
Female genitalia of *Scythrisandensis* Nupponen sp. nov., genus combination incertae sedis, paratype, slide 4/14 Dec. 2019 KN.

**Figure 72.**
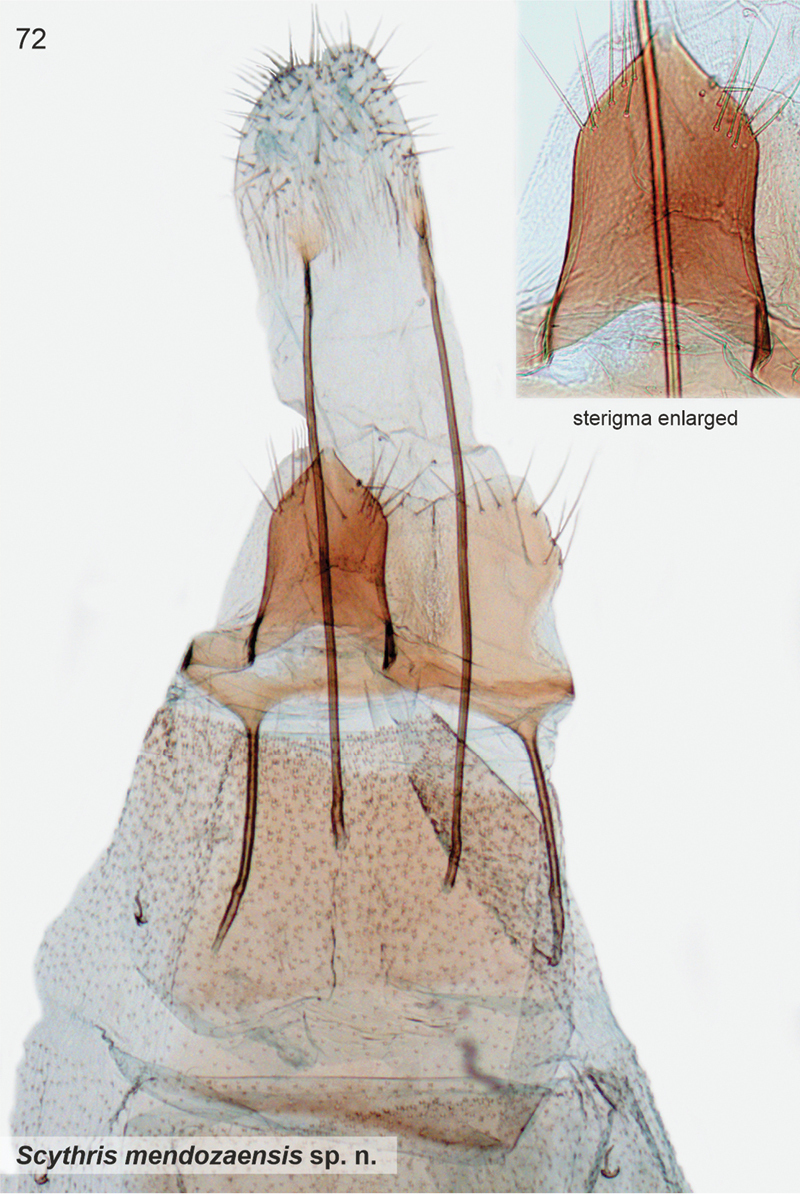
Female genitalia of *Scythrismendozaensis* Nupponen sp. nov., genus combination incertae sedis, holotype, slide 2/14 Dec. 2019 KN.

**Figure 73.**
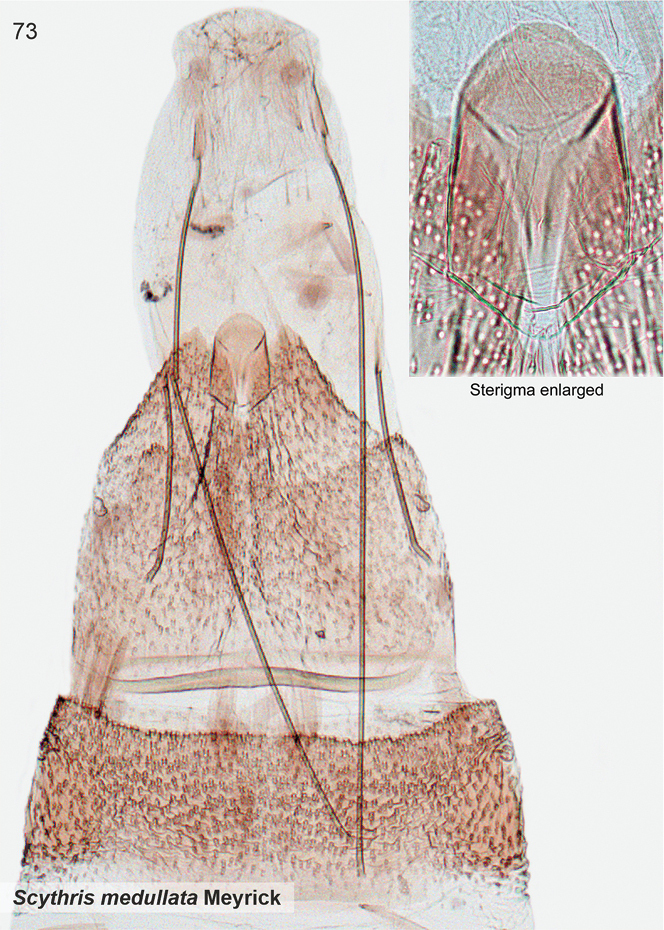
Female genitalia of *Scythrismedullata* Meyrick, 1916, genus combination incertae sedis, slide 2/17 Dec. 2019 KN.

**Figure 74.**
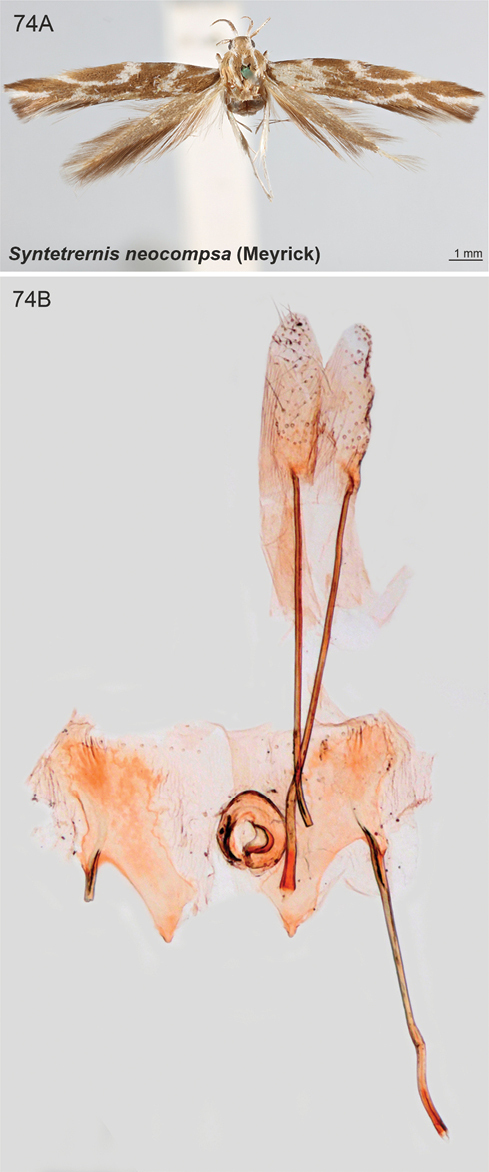
Holotype female of *Syntetrernisneocompsa* Meyrick, 1933, transferred from Scythrididae (Hodges 1997) to Cosmopterigidae incertae sedis (revised classification) **A** adult, Argentina: Alta Grazia (coll. NHMUK) **B** genitalia, slide JFGC No. 6153.

**Figure 75.**
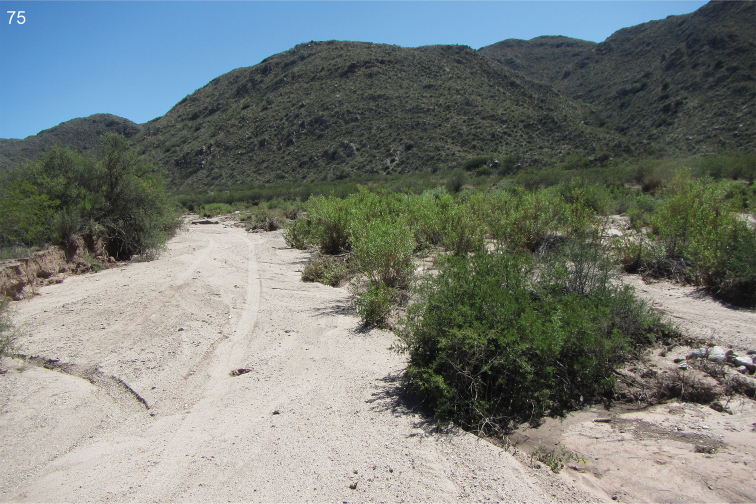
Collecting site of *Scythrisandensis* sp. nov. and *S.furciphallella* sp. nov.: Argentina, Andes Mts. (2085 m), Sierra de Famatina, 27 Jan. 2017.

**Figure 76.**
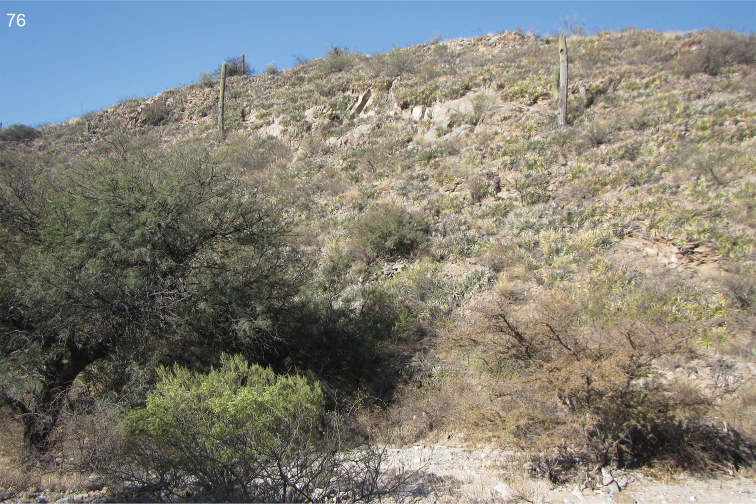
Collecting site of *Scythrismanchaoensis* sp. nov.: Argentina, Andes Mts. (1185 m), Sierra de Manchao, 23 Sep. 2017.

**Figure 77.**
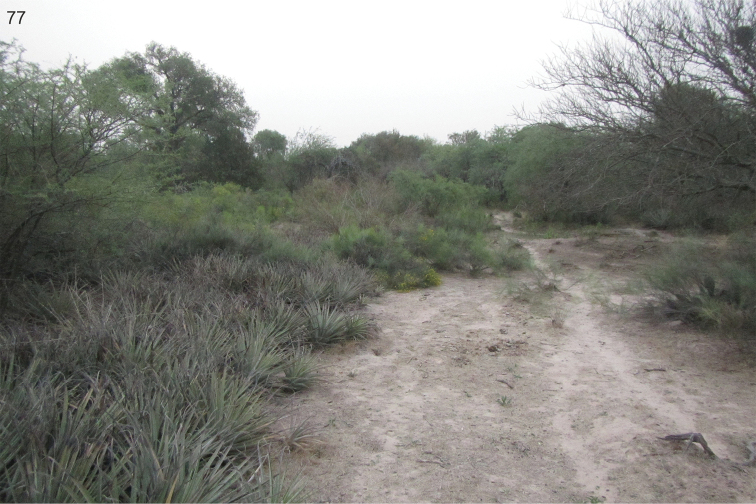
Collecting site of *Rhamphuradepressa* Meyrick, *R.pozohondaensis* sp. nov., *R.subdimota* sp. nov., *R.curvisociella* sp. nov., *Scythrisdirectiphallella* sp. nov., *S.angustivalvella* sp. nov., *Landryiaankylosauroides* sp. nov. Argentina, Pozo Honda vill. S (259 m), 19 Sep. 2017.

**Figure 78.**
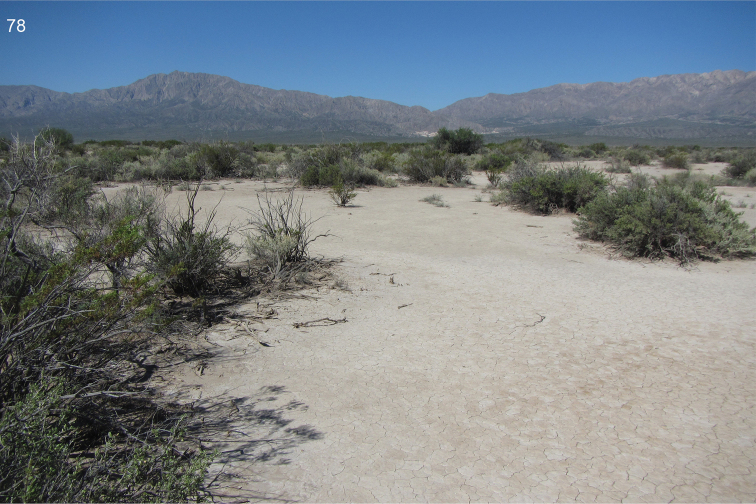
Collecting site of *Scythrisbicoloristrigella* sp. nov. and *Rhamphuraspiniuncus* sp. nov.: Argentina, Andes Mts. (1620 m), salt lake by Cordillera del Tigre, 26 Jan. 2017.

**Figure 79.**
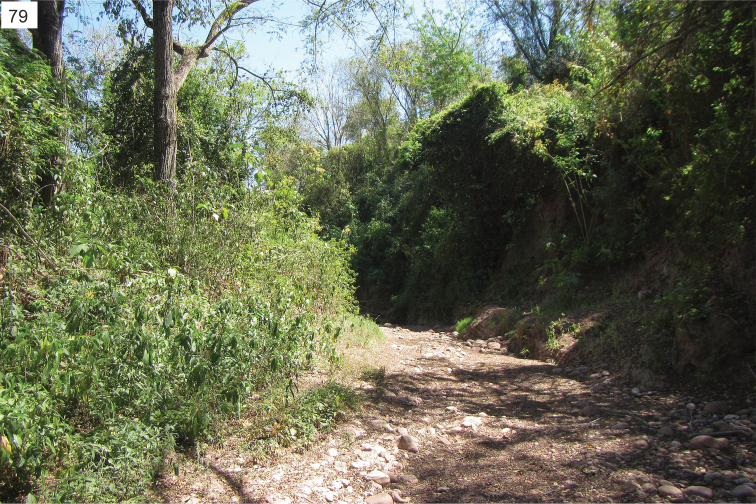
Collecting site of *Rhamphuraangulisociella* sp. nov., *Scythriscaimancitoensis* sp. nov. and *S.sanfranciscoi* sp. nov.: Argentina, Rio San Francisco (397 m), 18 Sep. 2017.

**Figure 80.**
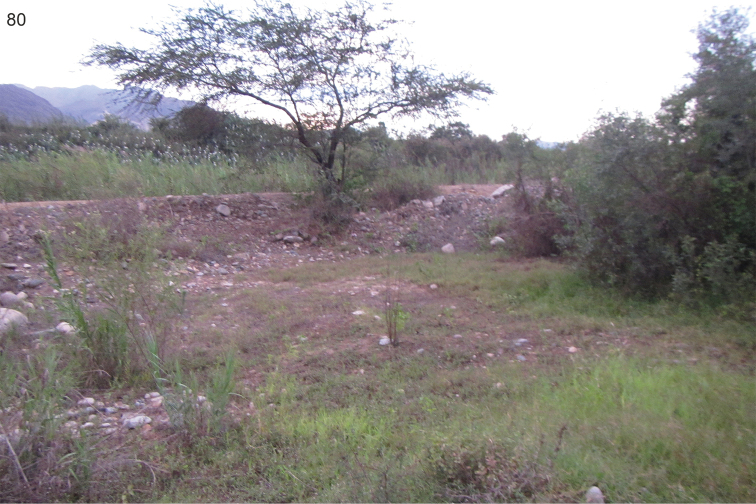
Collecting site of *Scythrislequetepequensis* sp. nov. and *S.medullata* Meyr.: Peru, Lequetepeque River shore (200 m), 1 Feb. 2019.

**Figure 81.**
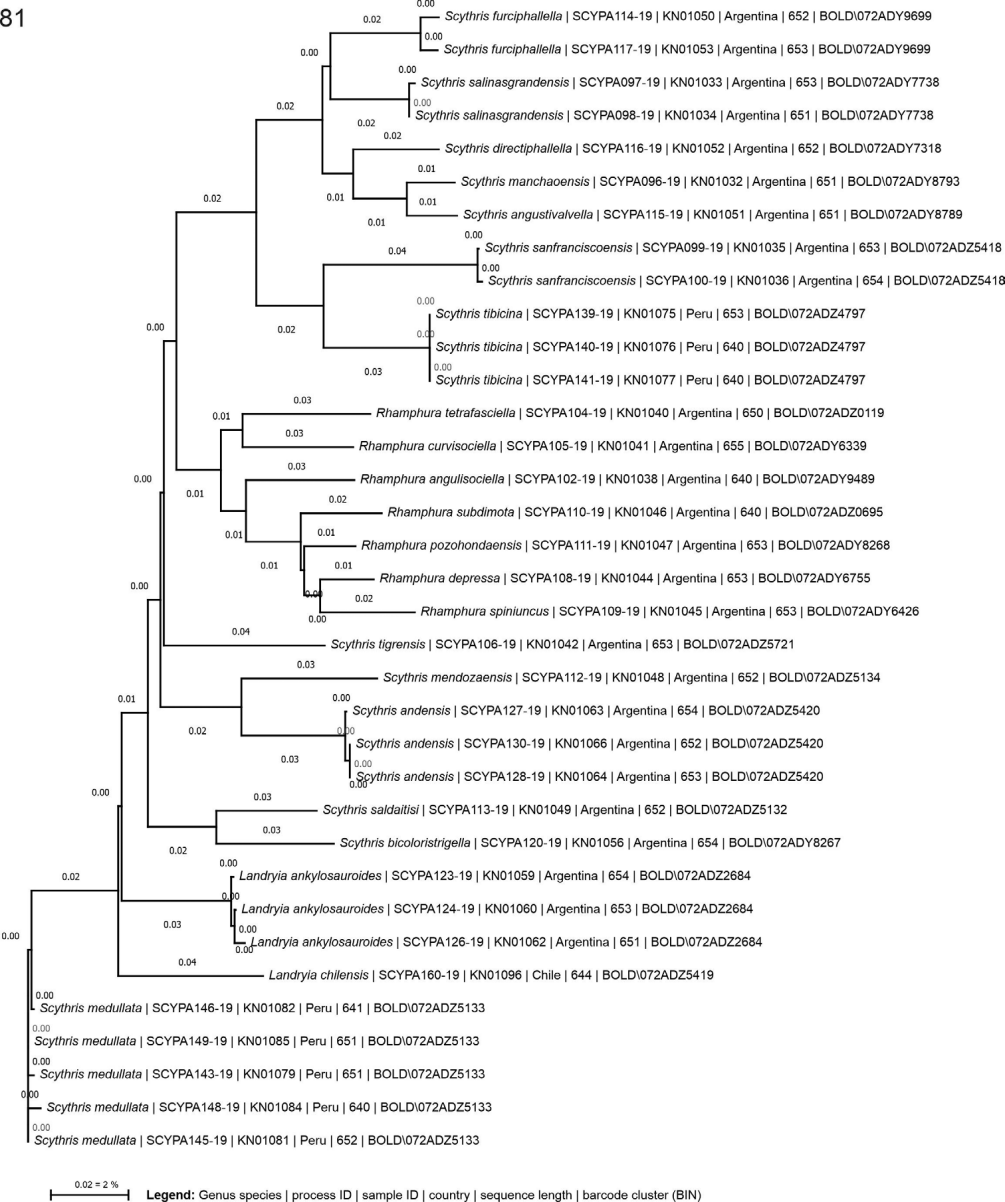
Neighbor-joining tree of 35 barcoded specimens generated from BOLD (Sujeevan and Hebert 2007, https://v4.boldsystems.org/ For each specimen, data are presented as shown at bottom of the tree. BOLD analysis parameters: taxon ID tree, Kimura 2-parameter model, BOLD aligner, contaminants excluded, records with stop codon excluded, records flagged as misidentifications or errors excluded, pairwise deletion, codon positions included: 1^st^, 2^nd^, 3^rd^.

####### Etymology.

Latinised adjective in the nominative singular. The name of the species refers to its geographical origin, the Andes Mountains.

####### Distribution.

NW Argentina.

####### Habitat.

The species was collected in a dry sandy river bed at medium altitude of the Andes Mts., surrounded by dry and xerothermic rocky slopes with low vegetation and sparse shrubs (Fig. 75).

####### Genetic data.

BIN: BOLD:ADZ5420 (*n* = 3 from Argentina). Genetically slightly heterogenous, maximum variation 0.16%. Nearest neighbour: *Scythrismendozaensis* Nupponen sp. nov. from Argentina (Scythrididae, BIN: BOLD:ADZ5134, 5.78%).

####### Remarks.

*Scythrisandensis* and *S.mendozaensis* are morphologically similar. In COI maximum likelihood phylogeny these taxa associate next to taxa, which are classified in *Scythris* or without genus combination on BOLD (Suppl. material 2). Structurally these taxa are not easy to combine to any North American Scythrididae genus (Landry 1991)). For these reasons we tentatively classify *andensis* and *mendozaensis* in *Scythris* (incertae sedis), highlighting the need for more research.

###### ﻿ Scythris mendozaensis

Taxon classificationAnimaliaLepidopteraScythrididae

Nupponen, sp. nov., genus combination incertae sedis

A9C7BC92-2D0A-5EBF-8A54-516A03C6BB6C

http://zoobank.org/158CC3B9-9F3A-40FF-828B-1F515DC38497

Figs 31 , 72

####### Type material.

***Holotype*.** Argentina • ♀; prov. Mendoza, Andes Mts., Cordillera del Tigre, Mendoza River valley near Uspallata village; 32°35.9'S, 69°22.9'W; 1900 m a.s.l.; 25 Jan. 2017; K. Nupponen & R. Haverinen leg.; [BOLD sample ID] KN01048; [genitalia slide] K. Nupponen prep. no. 2/14 Dec. 2019; coll. NUPP (MZH).

####### Diagnosis.

Externally resembles to some extent *S.notorrhoa* and some colour forms of *L.ankylosauroides*. White streak on forewing continues to tornus in *mendozaensis* (to termen in *notorrhoa* and *ankylosauroides*), the streak is narrow and dorsally without interrupted line (streak is broader in *notorrhoa*, and dorsally with interrupted line in *ankylosauroides*). In the female genitalia of *S.mendozaensis*, a large pentagonal sterigma is diagnostic. *Scythrismendozaensis* is known from 1900 metres altitude in the Anders, whereas *S.notorrhoa* is known from the Amazonian lowland rain forest.

####### Description.

Wingspan 13.5 mm. Head, collar, tegula, and thorax beige with scattered white. Few white scales exist around eye. Neck tuft and haustellum white. Scape dorsally beige, ventrally dirty white; pecten longer than diameter of scape. Flagellum dark brown, 0.7 × length of forewing. Labial palp white, except lower surface of palpomeres II and III brown. Legs white, tarsus and tibia mixed with beige. Abdomen dorsally beige, ventrally white. Forewing beige, fold widely white from base to tornus; indistinct brown blotches at dorsal margin of fold at 0.2 and 0.45; dark brown spot at cell end. Hindwing pale fuscous.

***Female genitalia*.** Sterigma large, twice as long as wide, pentagonal; anterior margin concave, posteriorly tapered and pointed. Ostium small, situated at posterior tip of sterigma. Sternum VII rectangular, undifferentiated. Apophyses anteriores 0.35 × length of apophyses posteriores.

####### Etymology.

Latinised adjective in the nominative singular. The species is named after the type locality, valley of the River Mendoza.

####### Distribution.

NW Argentina.

####### Habitat.

The collecting site at the type locality is a dry and xerothermic valley of the River Mendoza at medium altitude of the Andes, surrounded by rocky slopes with sparse and low vegetation.

####### Genetic data.

BIN: BOLD:ADZ5134 (*n* = 1 from Argentina). Nearest neighbour: *Scythrisandensis* Nupponen, sp. nov. (BIN: BOLD:ADZ5420, 5.78%).

####### Remarks.

Male unknown. *Scythrisandensis* and *S.mendozaensis* are morphologically similar. In COI maximum likelihood phylogeny these taxa associate next to taxa, which are classified in *Scythris* or without genus combination on BOLD (Suppl. material 2). Structurally these taxa are not easy to combine to any North American Scythrididae genus (Landry 1991)). For these reasons we tentatively classify *andensis* and *mendozaensis* in *Scythris* (incertae sedis), highlighting the need for more research.

##### The *dividua* species group

Phallus short and thick, basally more sclerotised. Valvae short and broad, potentially almost immobile. Male sternum VIII large asymmetrical plate, with stout apical pegs. Species included: *dividua*, *medullata*, *notorrhoa*.

*Scythrisdividua*, *S.notorrhoa*, and *S.medullata* are morphologically similar. The DNA barcode is available for *S.medullata* only, and in COI maximum likelihood phylogeny it associates next to taxa, which are classified in *Landryia* or without genus combination on BOLD (Suppl. material 2). However, structurally these taxa do not have the diagnostic characters of *Landryia* (treated as *Asymmetrura* in Landry 1991), such as the greatly enlarged bulbus ejaculatorius in the male genitalia, or the pincer-like projections on caudal margin of sternum VII on the female abdomen, and these three species are not easy to combine to any North American Scythrididae genus. For these reasons we tentatively classify *Scythrisdividua*, *S.notorrhoa*, and *S.medullata* in *Scythris* (incertae sedis). Relationship to *Neoscythris* is also possible, see Genetic data under *S.medullata*.

###### ﻿ Scythris dividua

Taxon classificationAnimaliaLepidopteraScythrididae

(Meyrick, 1916), genus combination incertae sedis

5E08D8D6-BAAC-5797-A211-79205AB58B51

Figs 32 , 60

Scythris dividua Meyrick, 1916. Exotic Microlepidoptera, vol. 2 (part 1): 12.

####### Material examined.

***Lectotype*.** Peru • ♂: Oroya; [11°31'S, 75°53'W]; 12200 feet a.s.l.; 7.14.; Parish leg.; [genitalia slide] JFGC No. 8054; NHMUK ID 010922357; NHMUK slide ID 010316666; coll. NHMUK.

***Paralectotypes*.** Peru • 11 exx.; same data as for lectotype; coll. NHMUK.

####### Diagnosis.

*Scythrisdividua*, *S.medullata*, and *S.notorrhoa* are similar externally. Reliable determination can be achieved by genitalia examination (DNA barcodes not available for all these three taxa yet). Uncus pentagonal, heavily sclerotised in *dividua*; rectangular, small, less sclerotised in *medullata*; oval and heavily sclerotised in *notorrhoa*. Valvae narrow basally, inner margin without sclerotisations in *dividua*; broad basally, inner margin with minute sclerotisation in *medullata*; asymmetrical, inner margin with large sclerotisations in *notorrhoa*. Segment VIII distinct in each three species, see illustrations.

####### Description.

The original description is quoted: “Wingspan 12­–15 mm ♂, ♀. Head, palpi and thorax dark bronzy-grey, somewhat sprinkled with whitish. Antennal ciliations of ♂ 1. Abdomen dark grey, in ♂ sprinkled with whitish beneath, in ♀ suffused with ochreous-whitish beneath and towards apex above. Forewings lanceolate; dark bronzy-grey, irregularly strewn with whitish scales, especially posteriorly; a cloudy white median streak from base to near termen, and a slenderer one close beneath it to beyond middle; an undefined subdorsal streak of obscure whitish irroration from base to tornus: cilia grey, mixed with white towards base. Hindwings 0.75, 4 and 5 separate; dark grey, thinly scaled anteriorly; cilia grey.”

***Male genitalia*.** Uncus pentagonal, heavily sclerotised plate. Tegumen trapezoid hood; anteriorly attached to broad transverse sclerotisation having anteriorly a rectangular extension with heavily sclerotised blunt tip. Phallus short and thick, basally more sclerotised (homology interpretation tentative, this structure could also be gnathos base). Valva short, basal rather narrow, distally broad and round. Saccus labiate, longer than valva. Sternum VIII large asymmetrical plate; basal portion rectangular with anterior apodemes, arched sclerotisation medially; posteriorly two large bifurcate processes, outer lobes distally asymmetrically extended, inner lobes with three stout apical spikes. Tergum VIII H-shaped; posterior shanks bent inwards, apices with five stout spiniform setae and bunch of thick setae; tip of anterior shanks foot-shaped.

***Female genitalia*.** Not dissected.

####### Distribution.

Peru.

###### ﻿ Scythris medullata

Taxon classificationAnimaliaLepidopteraScythrididae

(Meyrick, 1916), genus combination incertae sedis

2C1FB03D-9EB6-5073-B04E-50C6E0BC1788

Figs 33 , 61 , 73

Scythris medullata Meyrick, 1916. Exotic Microlepidoptera, vol. 2 (part 1): 13.

####### Material examined.

***Lectotype*.** Peru • ♂; Lima; 500 feet a.s.l.; 8–14.; Parish leg.; [genitalia slide] JFGC No. 8055; NHMUK ID 010922362; NHMUK slide ID 010316667; coll. NHMUK.

***Paralectotypes*.** Colombia, Equador, Peru • Meyrick (1916) described the species based on 80 specimens, but only 13 remain the NHMUK/Meyrick collection, also reported by Clarke (1965).

####### Other material.

Peru • 1 ♂, 3 ♀; prov. La Libertad, Lequetepeque River, near El Huabal village; 7°16.9'S, 79°18.2'W; 200 m a.s.l.; 1 Feb. 2019; K. Nupponen & R. Haverinen leg.; [BOLD sample IDs] KN01079, KN01080, KN01081, KN01084; [genitalia slide] K. Nupponen prep. no. 2/17 Dec. 2019 ♀; coll. NUPP. • 1 ♂, 1 ♀; prov. Cajamarca, Lequetepeque River, near Chilete village; 7°13.0'S, 78°45.3'W; 980 m a.s.l.; 4 Feb. 2019; K. Nupponen & R. Haverinen leg.; [BOLD sample IDs] KN01082, KN01083; [genitalia preparations] 2 in glycerol; coll. NUPP. • 1 ♂; prov. Ancash, Fortaleza River, Raquia village 13 km SW; 10°13.1'S, 77°33.6'W; 1180 m a.s.l.; 31 Jan. 2019; K. Nupponen & R. Haverinen leg.; [BOLD sample ID] KN01085; [genitalia slide] K. Nupponen prep. no. 3/17 Dec.2019; coll. NUPP. Argentina • 1 ♂; prov. Salta, Rio San Francisco, by Algarrobal village; 24°38.0'S, 64°54.5'W; 620 m a.s.l.; 16 Sep. 2017; K. Nupponen & R. Haverinen leg.; [BOLD sample ID] KN01039; [genitalia slide] K. Nupponen prep. no. 2/13 Dec. 2019; coll. NUPP.

####### Diagnosis.

*Scythrisdividua*, *S.medullata*, and *S.notorrhoa* are similar externally. Reliable determination can be achieved by genitalia examination (DNA barcodes not available for all these three taxa yet). Uncus pentagonal, heavily sclerotised in *dividua*; rectangular, small, less sclerotised in *medullata*; oval and heavily sclerotised in *notorrhoa*. Valvae narrow basally, inner margin without sclerotisations in *dividua*; broad basally, inner margin with minute sclerotisation in *medullata*; asymmetrical, inner margin with large sclerotisations in *notorrhoa*. Segment VIII distinct in each three species, see illustrations.

####### Description.

The original description is quoted: “Wingspan 11–12 mm ♂, ♀. Head, palpi and thorax dark violet-bronzy-grey, somewhat touched with whitish. Antennal ciliations of ♂ 0.75. Abdomen dark grey, suffused with ochreous-white beneath with both sexes. Forewings lanceolate; dark violet-bronzy-grey, either irregularly sprinkled with whitish except towards base, or with two closely adjacent whitish longitudinal streaks from base, upper median; reaching to about 0.75, lower reaching to beyond middle, and with every transitional variation between these two forms, the streaks and irroration varying in development but always one or the other present; plical and second discal stigmata more or less perceptible as obscure spots of dark fuscous suffusion, and sometimes one or two other similar spots in disc: cilia fuscous, variably mixed with whitish towards base. Hindwings 0.66, 4 and 5 separate; dark fuscous, thinly scaled anteriorly; cilia dark grey.”

***Male genitalia*.** Uncus rectangular, small. Gnathos base narrow belt; distal arm robust, rectangular with sclerotised tip. Tegumen hood-shaped. Phallus as long as gnathos, slim and shallowly bent, posterior quarter tapered, tip pointed. Valvae short, asymmetrical, broad, as long as gnathos; left valva slightly narrower, inner margin with minute sclerotisation, ventral margin with small sclerotised extension; right valva with semi-circular and heavily sclerotised extension at ventral margin, apical margin dentate. Sternum VIII large asymmetrical plate; basal portion rectangular with anterior apodemes, V-shaped reinforcement at middle; posterior part with two large extensions, left rectangular with horn-shaped lateral extension, right rectangular with rounded corners and posteriorly with seven long pegs. Tergum VIII small asymmetrical plate with bunch of long bristles.

***Female genitalia*.** Sterigma funnel-shaped, broad and rather short. Ostium round. Sternum VII trapezoid, medioposteriorly cleft, anterior margin chitinised. Apophyses anteriores short, one quarter length of apophyses posteriores.

####### Distribution.

Argentina, Colombia, Ecuador, Peru.

####### Habitat.

The moth inhabits moist riverside meadows (Fig. 80).

####### Genetic data.

BIN: BOLD:ADZ5133 (*n* = 6 from Costa Rica and Peru). Genetically slightly heterogenous, maximum variation 0.49%. Nearest neighbor: North American *Neoscythris* sp. (Scythrididae, BIN: BOLD:ABA1135, 0.29%).

####### Remarks.

New to Argentina. Originally the type series comprise 80 specimens, but only 13 exx. remain in the Meyrick collection (Colombia, Cali, 500 feet; Caldas 4400 feet; La Crumbre 6600 feet, in May. Ecuador, Huigra 4500 feet, in June; Peru, Lima 500 feet, in June; Chosica 2800 feet, in July and August (Parish). In the original description *S.medullata* is mentioned as an externally very variable species, and the variation being to some extent localised, the specimens from one locality being mostly externally similar.

###### ﻿ Scythris notorrhoa

Taxon classificationAnimaliaLepidopteraScythrididae

(Meyrick, 1921), genus combination incertae sedis

9C9D8E3E-C687-5D60-8AD9-38B0DE793769

Figs 34 , 62

Scythris notorrhoa Meyrick, 1921. Exotic Microlepidoptera, vol. 2 (part 14): 441.

####### Material examined.

***Lectotype*.** Brazil • ♂; Amazonas, Manaos; 11.19.; Parish leg.; [genitalia slide] JFGC No. 8065; NHMUK ID 010922363; NHMUK slide ID 010316671; coll. NHMUK.

***Paralectotype*.** Brazil • 17 exx.; same data as for lectotype; coll. NHMUK.

####### Diagnosis.

*Scythrisdividua*, *S.medullata*, and *S.notorrhoa* are similar externally. Reliable determination can be achieved by genitalia examination (DNA barcodes not available for all these three taxa yet). Uncus pentagonal, heavily sclerotised in *dividua*; rectangular, small, less sclerotised in *medullata*; oval and heavily sclerotised in *notorrhoa*. Valvae narrow basally, inner margin without sclerotisations in *dividua*; broad basally, inner margin with minute sclerotisation in *medullata*; asymmetrical, inner margin with large sclerotisations in *notorrhoa*. Segment VIII distinct in each three species, see illustrations.

####### Description.

The original description is quoted: “♂ ♀. 10–12 mm. Head bronzy-fuscous, sides ochreous-whitish, or in ♂ wholly suffused ochreous-whitish. Palpi fuscous more or less suffused ochreous-whitish. Thorax dark bronzy-fuscous, an ochreous-whitish, on inner side of patagia, in ♂ more or less suffused ochreous-whitish. Abdomen dark grey, beneath suffused ochreous-whitish. Forewings ♂ fuscous, ♀ dark bronzy-fuscous: a broad ochreous-whitish median stripe from base to termen, sometimes with slight apical projection above, dorsal area below this stripe in ♂ wholly suffused ochreous-whitish, plical stigma sometimes marked on lower margin of stripe: cilia grey, in ♂ paler and more or less suffused ochreous-whitish on termen. Hindwings 0.6, 4 and 5 separate; dark grey; cilia grey.”

***Male genitalia*.** Uncus heavily sclerotised, oval, slightly pointed on apex, surface granulate. Tegumen hood-shaped. Valvae asymmetrical, margins reinforced at basal 1/3: left valva longer, rather narrow, subapically with elongated and heavily sclerotised ventral extension, apex with long setae; right valva beyond middle with two large and complex heavily sclerotised extensions, apex with long setae. Phallus not detected on the lectotype slide (JFGC No. 8065). Sternum VIII large asymmetrical plate; basal portion rectangular; anteriorly with deep quadrangular concavity; posteriorly with two extensions: one short, the other triangularly extended with three stout apical pegs. Tergum VIII pentagonal, posteriorly extended, apically with six stout pegs; anteriorly with deep U-shaped concavity.

***Female genitalia*.** Not dissected.

####### Distribution.

Brazil.

####### Remarks.

Originally the type series comprised 80 specimens, but only 20 specimens remain in the Meyrick collection (Clarke 1965).

##### Taxon excluded from Scythrididae

###### ﻿ Syntetrernis neocompsa

Taxon classificationAnimaliaLepidopteraScythrididae

Meyrick, 1933, Cosmopterigidae incertae sedis

33A783EB-5C14-5F47-A2EB-B7CD79B801F0

Fig. 74

Syntetrernis neocompsa Meyrick, 1933. Exotic Microlepidoptera, vol. 4 (part 14): 428.
Scythris neocompsa (Meyrick, 1933). Transferred from Cosmopterigidae to Scythrididae: Scythris, but without evidence to support the transfer (Hodges 1997).

####### Material examined.

***Holotype*.** Argentina • ♀; Alta Grazia; CB. .32; C. Bruch leg.; [genitalia slide] JFGC No. 6153; NHMUK ID 010922354; NHMUK slide ID 010316673; coll. NHMUK.

####### Diagnosis.

Unmistakeable both externally (wings, thorax and head brownish-grey with white streaks) and by a characteristic ring-shaped sterigma in the female genitalia.

####### Description.

The original description is quoted: “Wingspan 17 mm ♂. Head light brownish-grey, white lateral streaks. Palpi white, second joint with grey subapical ring, terminal joint with grey lateral line. Thorax light brownish-grey, five light lines. Forewings narrow-lanceolate; grey-brownish; markings white; a short very oblique streak from base of costa; a fine line on dorsal edge towards base; an oblique streak from costa at 0.2 to fold; an oblique streak from costa at 0.4 in an even curve through middle of disc to costa at 0.8; a line running from fold at 0.4 to dorsum in middle of wing, thence to disc at 0.66, and returning to termen beyond tornus; a streak from disc at 0.75 to apex: cilia light brownish-grey, a white bar at apex and finer one above tornus. Hindwings and cilia grey.”

***Female genitalia*.** The genitalia are partly destroyed by museum pests. Sterigma thick ring-shaped. Ostium round, situated approximately at middle of sterigma, lateral margins reinforced by semi-circular and arched sclerotisations. Apophyses anteriores 0.8 × length of apophyses posteriores.

####### Distribution.

Argentina.

####### Remarks.

Male unknown. *Syntetrernisneocompsa* Meyrick, 1933 is transferred from Scythrididae (Hodges 1997) to Cosmopterigidae incertae sedis, following a consultation with Jean-François Landry (pers. comm.): “An examination of the type, shown in Fig. 74, strongly suggests that it is not a Scythrididae. The long, sickle-shaped labial palps are not found in scythridids; some spatulate scales are discernible on the head (vertex), and such scales are not found in scythridids but in other gelechioid families such as Cosmopterigidae, Momphidae, and Gelechiidae. The forewing pattern is atypical of scythridids (not trustworthy by itself but significant combined with the other characters). The female genitalia (Fig. 74B) shows a sterigma that is reminiscent of some Cosmopterigidae (e.g.*Triclonella* Busck, 1901, *Hyposmocoma* Butler, 1881, *Asymphorodes* Meyrick, 1929). These features suggest that this is likely a Cosmopterigidae. The original genus *Syntetrernis* was transferred to Parametriotinae (now in Elachistidae) by Hodges (1997), however, the taxon *neocompsa* doubtfully matches that subfamily.” The male is unknown.

## ﻿Discussion

The results of our three expeditions show that the Scythrididae fauna of the study area is mostly unknown. This study brings the total of described species of the family Scythrididae from continental South America to 35. It is difficult to estimate the actual species richness in South America, except to note that it is estimated that the undescribed Scythrididae species outnumber described ones by a factor of ten in the more extensively explored North America (Hodges et al. 1983; Landry 1991). In North America 44 species are included in the family (Pohl et al. 2016), but the actual number of species is possibly between 400 and 500 species (Landry 1991). Bengtsson (2014) reported 307 species from Africa, but Agassiz (2014) speculated the fauna of Africa to be perhaps several times higher. Of the 25 species collected by the first author for the current paper, 22 (91%) were species new to science. With 60% of recorded species represented by a single specimen only, more extensive collecting is obviously needed to document the actual species richness and abundance. This also effects the identification key and the authors expect that the key to males will be out of date as soon as more South American material is examined.

The area with the highest species richness appears to be the eastern slopes of the Andes at medium and low elevations (~ 180–2100 meters). All Scythrididae in the study area were attracted to light, and despite considerable efforts, not a single specimen was found during the day.

Recently, taxonomic revisions that rely on DNA barcodes and photographs of external features alone have started to gain ground. One example of such minimalist approach is the revision of Costa Rican braconid wasps (Sharkey et al. 2021). Such studies are tempting and pragmatic if the fauna is unexplored, because arguably the identities are less subjective, it takes less taxonomists’ time to prepare, and those are easier to update. Such approaches have been criticised, particularly because in the long run they do not speed up description of the biodiversity, but rather introduce “superficial taxonomic impediment” for future generations of taxonomists (Meier et al. 2021). We justify our classical approach by the historical Meyrick material, which we included in the study, because with regard to this material we had to rely on morphology alone. Meyrick’s type specimens have not been DNA barcoded yet. Our approach was very time consuming, but by treating the new material in a similar manner, we aimed to make all South American Scythrididae material better accessible for future studies. We managed to provide DNA barcodes for 19 of the 22 new species described in the current paper, and also for three of the 13 species described by Meyrick, the latter being based on fresh material.

On several occasions, it was difficult to combine South American Scythrididae into the existing classification (Landry 1991). Often some morphological similarity is evident, particularly in the genitalia structures, but repeatedly it was doubtful whether the existing genus definitions should be broadened to encompass the observed variation, or should new genera be described to highlight the differences. *Rhamphura* is a case in point: the morphology of the taxa *subdimota*, *depressa*, *pozohondaensis*, and *spiniuncus* agrees well with the morphological definition of *Rhamphura* as in Landry (1991), while the taxa *angulisociella*, *tetrafasciella*, and *curvisociella* are more heterogenous and possess varying degrees of similarities to the North American *Rhamphura*. This finding was also supported by the COI maximum likelihood phylogeny (Suppl. material 2). For practical reasons, we adopted the view used in Landry (1991), and instead of describing new genera we classified such obscure taxa in existing genera with an incertae sedis note, highlighting the need for further research.

Molecular phylogenies focusing on Scythrididae are not yet available, and thus far Scythrididae have been represented in molecular phylogenies by few genera only out of at least 25 genera considered valid globally (Passerin and Roggero 2007). The molecular studies, which have included Scythrididae, have focused on resolving the family-level relationships in Gelechioidea (e.g., Kaila et al. 2011; Heikkilä et al. 2014; Wang and Li 2020). The most detailed phylogenetic analysis focused on supraspecific taxa in the North American fauna, and it is based on morphology (Landry 1991). Even though our COI maximum likelihood phylogeny (Suppl. material 2) is limited in terms of molecular data, and cannot considered but a first pass phylogeny, it agrees with Landry’s (1991) hypothesis in several points. This supports the view that DNA barcode sequences can form a highly valuable source of complementary information to supplement morphological data, and could help resolve controversial taxonomic issues not only at the species level, but also at the genus level (Breitling 2019a, b). The genus *Scythris* is the most heterogenic in terms of genital characters in our study, agreeing with similar notion of Landry (1991). This suggests it may not be monophyletic. In our COI phylogeny *Scythris* was recovered as a large monophyletic lineage, but with several genetically distant lineages. This heterogeneity may be better communicated in a classification that includes species groups within *Scythris*, or where separate genera are recognised.

Against this background it is obvious that a global phylogenetic framework for Scythrididae is needed, preferably using an integrative approach to include both molecular and morphological data. Only this will bring stability to the classification and will provide a basis to define homological structures in a group, which is morphologically among the most diverse in Lepidoptera.

We hope our paper could act as a starting point to increase future interest to study the species richness and systematics of Scythrididae in South America, and eventually also life histories.

## Supplementary Material

XML Treatment for Rhamphura depressa

XML Treatment for Rhamphura dimota

XML Treatment for Rhamphura subdimota

XML Treatment for Rhamphura immunis

XML Treatment for Rhamphura pozohondaensis

XML Treatment for Rhamphura spiniuncus

XML Treatment for Rhamphura angulisociella

XML Treatment for Rhamphura curvisociella

XML Treatment for Rhamphura tetrafasciella

XML Treatment for Landryia

XML Treatment for Landryia ankylosauroides

XML Treatment for Landryia chilensis

XML Treatment for Scythris directiphallella

XML Treatment for Scythris furciphallella

XML Treatment for Scythris manchaoensis

XML Treatment for Scythris salinasgrandensis

XML Treatment for Scythris angustivalvella

XML Treatment for Scythris zeugmatica

XML Treatment for Scythris caimancitoensis

XML Treatment for Scythris ejiciens

XML Treatment for Scythris fluvialis

XML Treatment for Scythris inanima

XML Treatment for Scythris lequetepequensis

XML Treatment for Scythris plocogastra

XML Treatment for Scythris tibicina

XML Treatment for Scythris sanfranciscoensis

XML Treatment for Scythris tigrensis

XML Treatment for Scythris bicoloristrigella

XML Treatment for Scythris saldaitisi

XML Treatment for Scythris wikstromi

XML Treatment for Scythris andensis

XML Treatment for Scythris mendozaensis

XML Treatment for Scythris dividua

XML Treatment for Scythris medullata

XML Treatment for Scythris notorrhoa

XML Treatment for Syntetrernis neocompsa

## Supplementary materials

Supplementary material 1 Supplementary file 1 Data type GenBank accession numbers (xslx. file) Explanation note DNA barcodes developed for this article (35 specimens), the associated GenBank accession numbers and other metadata. https://binary.pensoft.net/file/649354This dataset is made available under the Open Database License (http://opendatacommons.org/licenses/odbl/1.0/). The Open Database License (ODbL) is a license agreement intended to allow users to freely share, modify, and use this Dataset while maintaining this same freedom for others, provided that the original source and author(s) are credited.Kari Nupponen, Pasi Sihvonen

Supplementary material 2 Supplementary file 2 Data type Phylogenetic hypothesis (pdf. file) Explanation note Maximum likelihood phylogeny based on 728 COI barcode sequences (including 3 outgroups). Parameters: minimum 500 bp per each sample, W-IQ-TREE (Trifinopoulos et al. 2016), ultrafast bootstrap (1000 replicates), GTR+F+I+G4 model. Data includes all public COI samples available on BOLD in September 2001 with taxon name ”Scythrididae” from North, Central and South America. New COI barcodes published in this paper are highlighted with red. Right margin includes tentative genus classification for the included samples. https://binary.pensoft.net/file/649355This dataset is made available under the Open Database License (http://opendatacommons.org/licenses/odbl/1.0/). The Open Database License (ODbL) is a license agreement intended to allow users to freely share, modify, and use this Dataset while maintaining this same freedom for others, provided that the original source and author(s) are credited.Kari Nupponen, Pasi Sihvonen
