# Supplementary material for: ﻿Revision of Neotropical Scythrididae moths and descriptions of 22 new species from Argentina, Chile, and Peru (Lepidoptera, Gelechioidea)
Source: Zookeys. 2022 Feb 22;1087:19–104. doi: 10.3897/zookeys.1087.64382 (PMC8888540; doi:10.3897/zookeys.1087.64382)

**Supplement file 2.** Maximum likelihood phylogeny based on 728 COI barcode sequences (including 3 outgroups). Parameters: minimum 500 bp per each sample, W-IQ-TREE (Trifinopoulos et al. 2016), ultrafast bootstrap (1000 replicates), GTR+F+I+G4 model. Data includes all public COI samples available on BOLD with taxon name ”Scythrididae” from North, Central and South America. New COI barcodes published in this paper are highlighted with red. Right margin includes tentative genus classification for the included samples.

Nupponen, K. Sihvonen, P. Revision of Neotropical Scythrididae fauna and descriptions of 22 new species from Argentina, Chile and Peru (Lepidoptera: Scythrididae).

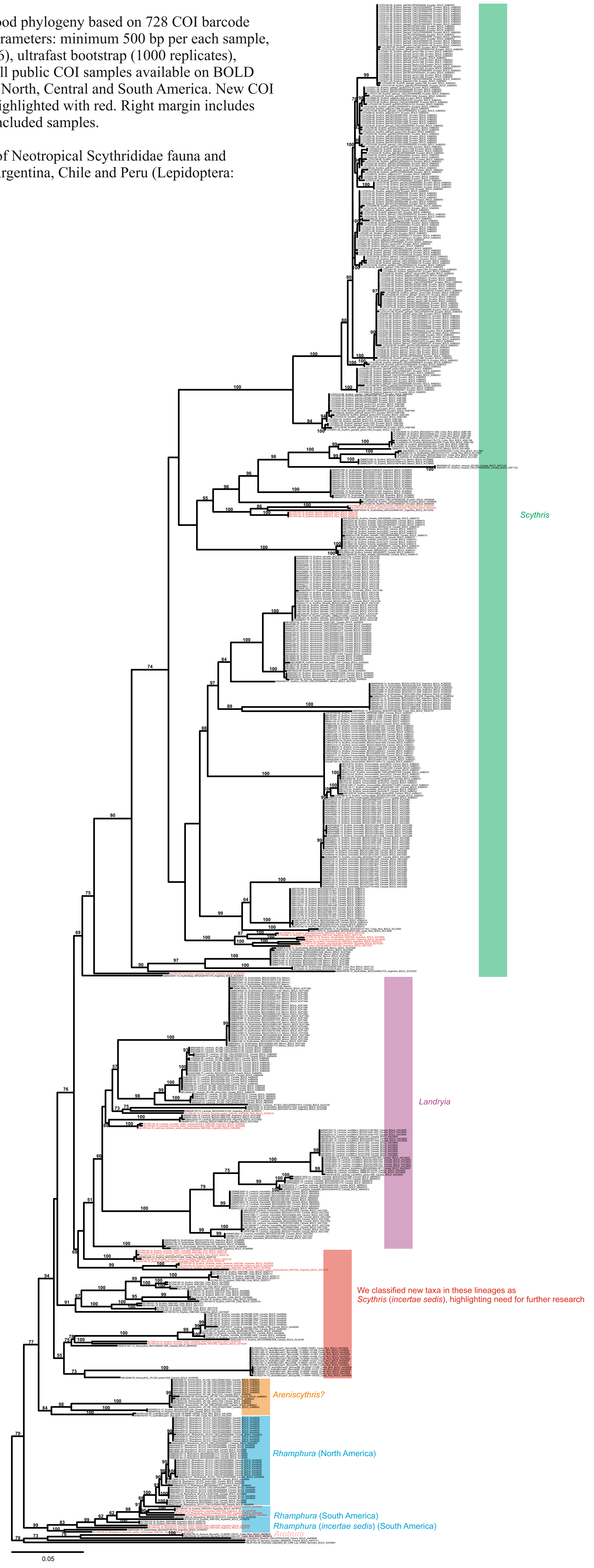

Supplement: Supplementary material 2 — Supplementary file 2 [file zookeys-1087-019-s002.pdf]
